# Climate and COVID-19 transmission: a cross-sectional study in Africa

**DOI:** 10.1038/s41598-023-46007-0

**Published:** 2023-10-31

**Authors:** Ousmane Koanda, Roland Yonaba, Fowé Tazen, Héla Karoui, Mohamed Lamine Sidibé, Babacar Lèye, Mamadou Diop, Harinaivo Anderson Andrianisa, Harouna Karambiri

**Affiliations:** 1 Laboratoire Eaux, Hydro-Systèmes et Agriculture (LEHSA), Institut International d’Ingénierie de l’Eau et de l’Environnement (2iE), Ouagadougou, Burkina Faso; 2 Laboratoire Eco-Matériaux et Habitat Durable (LEMHaD), Institut International d’Ingénierie de l’Eau et de l’Environnement (2iE), Ouagadougou, Burkina Faso

**Keywords:** Environmental impact, Sustainability

## Abstract

The role of climate in the Coronavirus disease 2019 (COVID-19) transmission appears to be controversial, as reported in earlier studies. In Africa, the subject is poorly documented. In this study, over the period from January 1st, 2020 to September 31, 2022, the daily variations in cumulative confirmed cases of COVID-19 for each African country (54 countries) are modelled through time-series-based approaches and using meteorological factors as covariates. It is suggested from the findings that climate plays a role in COVID-19 transmission since at least one meteorological factor is found to be significant in 32 countries. In decreasing order, the most often occurring meteorological factors are dewpoint temperature, relative and absolute humidity, average temperature and solar radiation. Most of these factors show a lagged effect with confirmed cases (between 0 and 28 days). Also, some meteorological factors exhibit contrasting effects on COVID-19 transmission, resulting in both positive and negative association with cumulative cases, therefore highlighting the complex nature of the interplay between climate and COVID-19 transmission.

## Introduction

In December 2019, a transmissible disease emerged in the city of Wuhan (in Hubei province, China). The disease was found to be caused by a new coronavirus, the *Sars-CoV-2*^1^. Initially called *2019-nCOV* acute respiratory disease, later renamed *COVID-19* disease, it was declared a pandemic by the World Health Organization (WHO) on March 11, 2020, and further acknowledged as a Public Health Emergency of International Concern (PHEIC)^2^. The disease quickly spread throughout the world, starting in January 2020. Despite the wide array of restrictive measures largely promoted by governments worldwide^3,4^, the outbreak of the pandemic has been largely unprecedented: a year later, on December 31, 2020, a total of 83,638,090 cumulative confirmed cases and 1,880,710 deaths were reported globally, hence a case-fatality rate of 2.25%^5^.

Even though at the global scale, the outbreak of the COVID-19 disease has been exponential, the African continent emerges as the least affected in the World, according to the numbers of confirmed cases and deaths reported. The first confirmed case in Africa was reported in Egypt on February 14, 2020. On December 31, 2020, a total of 2,760,450 cumulative confirmed cases and 65,458 deaths (respectively 3.30% and 3.48% of the global count) were reported. As of September 30, 2022, the total cumulative confirmed cases in Africa rose to 12,355,709 (2.0% of the global total), while the number of deaths reached 256,764 (3.9% of the global total)^5^. These estimates seem low compared to those from America, Europe and Asia, which reported respectively 44.6%, 28.6% and 24.8% of the global count of confirmed cases^5,6^. Yet, the case-fatality rate of the disease appears to be almost twice as high in Africa (2.08% as of September 30, 2022) as compared to the global level (1.08%), indicating the higher lethality of the disease in Africa^7^.

A typical trait regarding the spatial patterns of the spread of the pandemic is that, at the scale of the African continent, large discrepancies are observed among countries. As of September 30, 2022, for example, South Africa alone reported 32.5% and 39.8% of the total confirmed cases and deaths (respectively) in Africa. Meanwhile, the top five hard-hit countries (*in decreasing order of severity*: South Africa, Morocco, Tunisia, Egypt and Libya) reported 60.3% and 69.7% of the total confirmed cases and deaths (respectively)^5^. These figures are thought to be due to specific traits of the African context, including the low rate of urbanization, the limited transport network hindering the mobility of people, and the young age of the population^8,9^. Also, the lower case fatality rate in Africa of non-communicable diseases such as cancer, cardiovascular accidents and diabetes (already known as comorbidities in this pandemic) has been often put forward^7,10^.

Among the factors affecting the transmissibility of COVID-19, the role of climate conditions has been early investigated and is still a trending and active topic^11^. Meteorological conditions can influence how the virus survives and persists within a given environment, which in turn might affect the likelihood of transmission of the disease^12^. Overall, most of the available studies tend to report lower transmission rates at warmer temperatures but higher transmission in colder and dry environments^9,13^, along with exacerbating effects of humidity, higher air concentration in fine particles^14^, lower wind speeds^15^ or lower solar radiation^9^. For example, in South Asia, Hossain et al.^12^ identified a positive association between COVID-19 confirmed cases and minimum, maximum and average temperatures, rainfall, relative air humidity, wind speed and surface pressure. In the US, Bashir et al.^16^ reported a positive association between COVID-19 cases and temperatures, but a negative association with rainfall, relative air humidity, wind speed and surface pressure. Zhuang et al.^17^ reported similar findings based on evidence from 216 cities in China. Temperature and relative humidity are, on average, the factors the most frequently associated with COVID-19 cases^18^. Wang et al.^19^ reported for instance that a 1 °C rise in average temperature might be responsible for a 3.1% decrease in the daily new cases of COVID-19 infections and a 1.2% decrease in related deaths. Other meteorological parameters, such as wind speed and air pollutants are also reported as likely influential on COVID-19 transmission rates^12,20^. Solar radiation and geographical latitude (representing the distance to the equator) have also been identified as significant in explaining COVID-19 deaths^9,13^. Moreover, the relationship between climate and COVID-19 transmission is thought to be complex because weather patterns in a year affect human behaviour, therefore affecting the observation of lifting of containment measures. For example, Ganslmeier et al.^21^ reported that weather substantially affects the likelihood of social gatherings, therefore increasing the spread of the disease. Also, previous research indicates that including human behaviour and climate is critical in modelling the spread of a disease outbreak^22^.

Previous modelling studies attempted to analyse the spread of COVID-19 in different contexts, mainly to forecast the pandemic prevalence or assess the potential effect of meteorological conditions (as covariates) on COVID-19^23^. The modelling methods often used range from generalized additive models^24^, generalized linear models^24^, compartmental epidemiologic approaches based on *Susceptible, Infectious or Recovered* (SIR) models^25–27^, and artificial neural networks^28,29^. Among these methods, time series-based modelling methods such as *Autoregressive Integrated Moving Average* (ARIMA) have been largely used for COVID-19 modelling and to explore the potential effect of external regressors such as meteorological variables on COVID-19 transmission^30–32^.

However, despite the huge body of available literature, still, few studies addressed the case of Africa as a whole, especially regarding the specific role of meteorological conditions on COVID-19 transmission. There is still a need to assess which and to what extent climate factors influence the transmission of COVID-19^33–36^. The question is of importance to African countries, especially in building effective, well-informed and adapted coping strategies for the ongoing pandemic^37^.

In this study, we aimed to provide a continental scale assessment of the potential isolated effect of meteorological conditions on COVID-19 prevalence in the specific context of Africa. Our objectives are twofold: (i) identify the climatic factors which significantly affect COVID-19 transmission in African countries; (ii) assess the isolated level of association of these climatic factors to COVID-19 and their relative importance across African countries. In this scope, the study uses publicly available data on COVID-19 prevalence accumulated over 33 months, from January 1, 2020, to September 30, 2022, which is deemed appropriate to investigate the potential long-term effects of meteorological conditions on COVID-19 transmission.

## Results

### Figures of the COVID-19 pandemic in Africa

#### Cumulative cases and waves

According to official reports^5,6^, the first confirmed case of COVID-19 was reported in Egypt (EGY) on February 14, 2020; it was shortly followed by countries mostly from Northern Africa (NAf) region, especially Algeria (DZA), Tunisia (TUN) and Morocco (MAR)^9^. Over time, East Africa (EAf) and Southern Africa (SAf) emerged as the regions where the incidence of the pandemic is the largest. These regions witnessed three waves of increasing magnitudes: (i) in January 2021, 500–750 daily new confirmed cases per million; (ii) in May–July 2021, 1500–1700 daily new confirmed cases per million; and (iii) in January 2022, 2000–3000 new daily confirmed cases per million). West Africa (WAf) and Northern Africa (NAf) are the second largest regions affected, with waves occurring at the same periods, although roughly similar in magnitude, with 700–900 new daily confirmed cases per million. Central Africa (CAf) is the least affected region in the continent (further details are provided as Supplementary Material, see Figs. S1, S2 and Table S1).

#### Spatial patterns of spread of the pandemic in Africa

**Figure 1:**
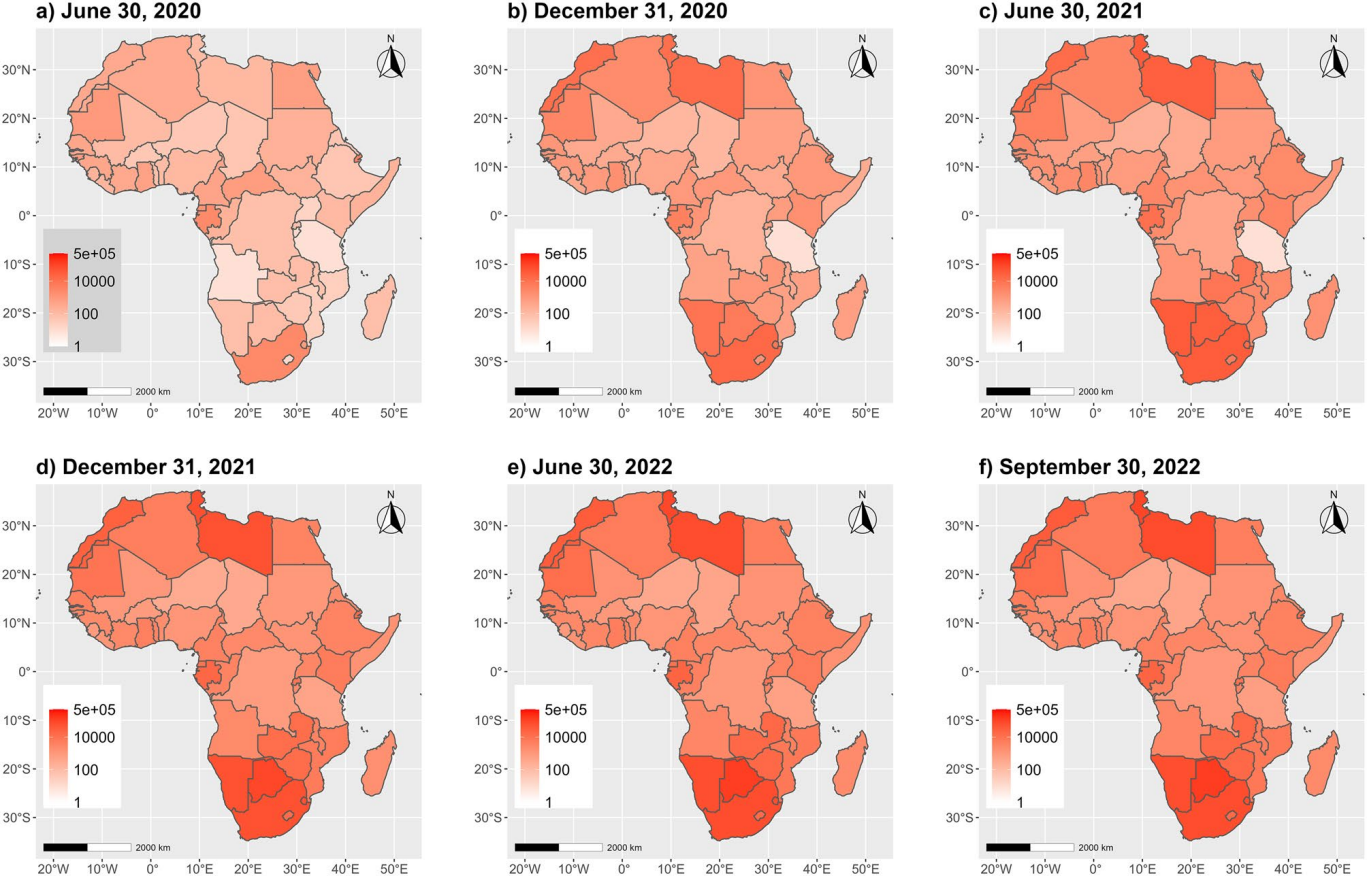
Spatial and temporal evolution of COVID-19 cumulative cases (per million) in Africa. The maps were produced using the *ggmap*^40^ and *ggplot2*^41^ visualisation libraries under the R programming language^42^.

The spatial patterns of evolution of the COVID-19 are presented in choropleths maps in Fig. 1. It appears that at the onset of the pandemic (January 1st to December 31, 2020), the countries located at the northern and southern poles of the continent were the most affected. This is likely attributable to the fact that such countries feature the highest rates of international trade and tourism^9^ and are gateways to the African continent^9,38,39^. However, over this period and the following year, there is still no clear emerging spatial clustering or organization of the COVID-19 pandemic at the global continental level. Towards September 30, 2022, countries around the equator emerge as the least affected, while those at the northernmost and the southernmost poles of the continent appear as the highly affected ones.

The assessment of the global spatial auto-correlation in COVID-19 incidence is presented in Table 1, which shows that until June 30, 2022, there is no significant global spatial autocorrelation at the continental level (Moran’s *I* p-values > $\alpha$  = 5%). From December 31, 2021 until September 30, 2022, a significant global spatial auto-correlation is emerging (Moran’s *I* p-values < $\alpha$  = 5%), likely because countries in the low-low cluster (countries with lower incidence surrounded by similar neighbouring countries) appear spatially located near the equator (Western, Central and East Africa regions), while highly affected countries remain altogether located at the continent poles.

**Table 1:** Global Moran's *I* for spatial auto-correlation in COVID-19 incidence.

Dates	Observed *I*	Expected ${I}_{0}$	Standard deviation	p-value
6/30/2020	− 0.0123	− 0.0189	0.0224	0.7684
12/31/2020	0.0235	− 0.0189	0.0233	0.0693
6/30/2021	− 0.0062	− 0.0189	0.0165	0.4437
12/31/2021	0.0189	− 0.0189	0.0186	0.0425*
6/30/2022	0.0174	− 0.0189	0.0176	0.0394*
09/30/2022	0.0177	− 0.0189	0.0177	0.0393*

*I* is the computed Global Moran. ${\mathrm{I}}_{0}$ is the expected value of $\mathrm{I}$ under the null hypothesis; P-values appended with ‘*’ are significant at $\mathrm{\alpha }$ = 5% level.

### Analysis of meteorological conditions

#### Summary description and spatial patterns of meteorological conditions

**Figure 2:**
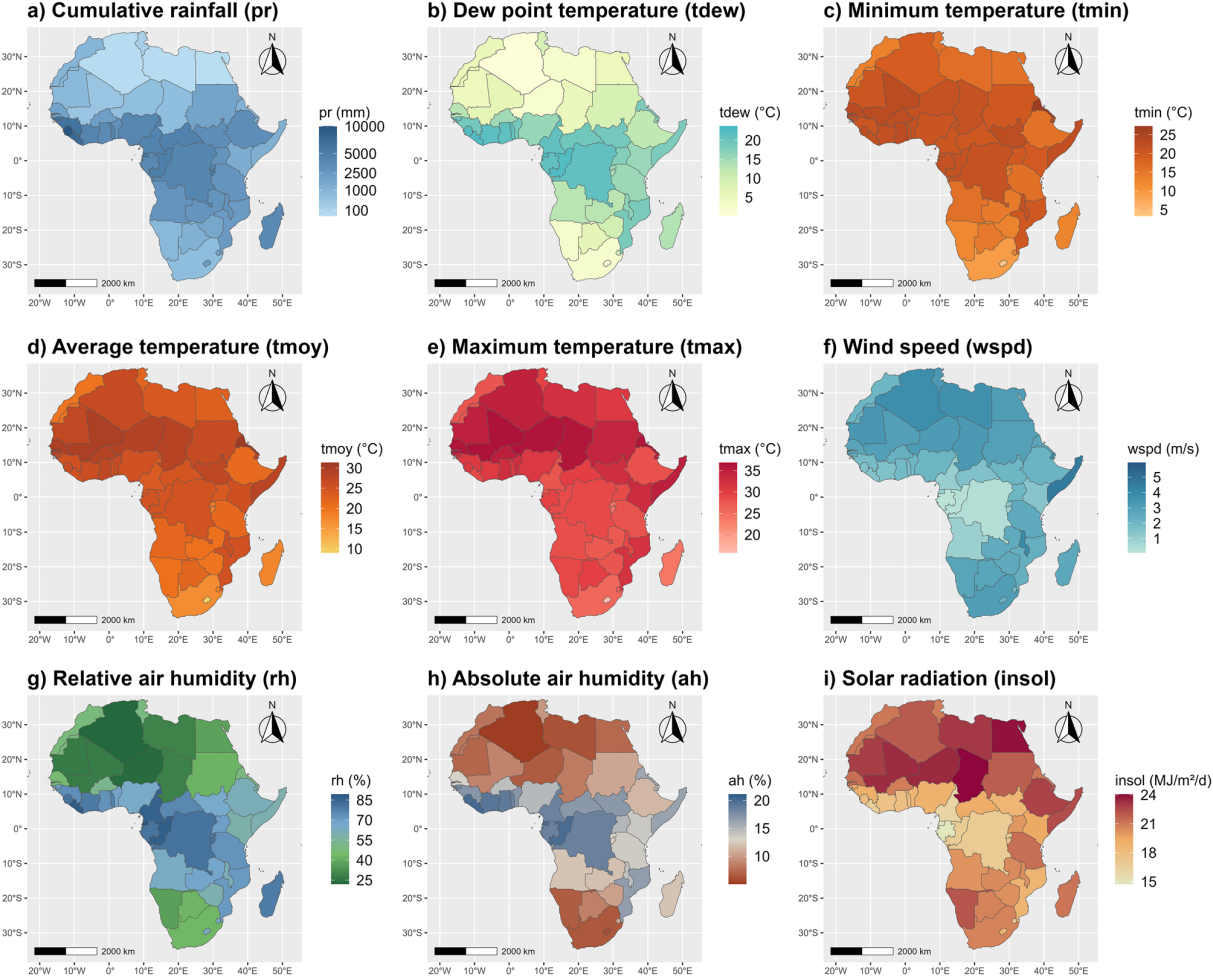
Spatial repartition of meteorological variables in Africa. The maps were produced using the *ggmap*^40^ and *ggplot2*^41^ visualisation libraries under the R programming language^42^.

The spatial patterns of meteorological conditions in Africa are presented in Fig. 2. The driest median cumulative rainfall (*pr*) is observed for the NAf region (202.3 mm), whereas it increases to 2580.7–4564.7 mm in other regions. NAf region also shows the lowest median dew point temperature (*tdew* = 5.1 °C), which increases to 12.1 °C (SAf region) and 21.0 °C (CAf region). The highest minimum (*tmin*), average (*tmoy*) and maximum (*tmax*) median temperatures are observed in the WAf region (22.2 °C, 26.6 °C and 32.8 °C respectively), whereas the lowest values are reported in the SAf region (14.2 °C, 20.6 °C and 27.2 °C respectively). The NAf region also shows the lowest relative (*rh*) and absolute (*ah*) median humidity (38.7°% and 7.3% respectively), but the highest median wind speed (3.2 m/s). In contrast, the median relative (*rh*) and absolute (*ah*) humidity in the other regions range between 63.2% (EAf region)-81.3% (CAf region) and 11.6% (SAf region)-18.4% (CAf region). Wind speed (*wspd*) is also generally lower in WAf (median of 2.2 m/s), EAf (median of 2.1 m/s), SAf (median of 2.7 m/s) and CAf (median of 1.0 m/s). Finally, solar radiation (*insol*) shows the highest values in the NAf region (median of 22.0 MJ/m^2^/day) and the lowest values in the CAf region (median of 16.9 MJ/m^2^/day). The correspondence between countries and regions is given as Supplementary Material in Table S2, and further details on the statistical description of each climate variable according to each region in the continent are given as Supplementary Material in Table S3.

#### Inter-correlations between climate variables

**Figure 3:**
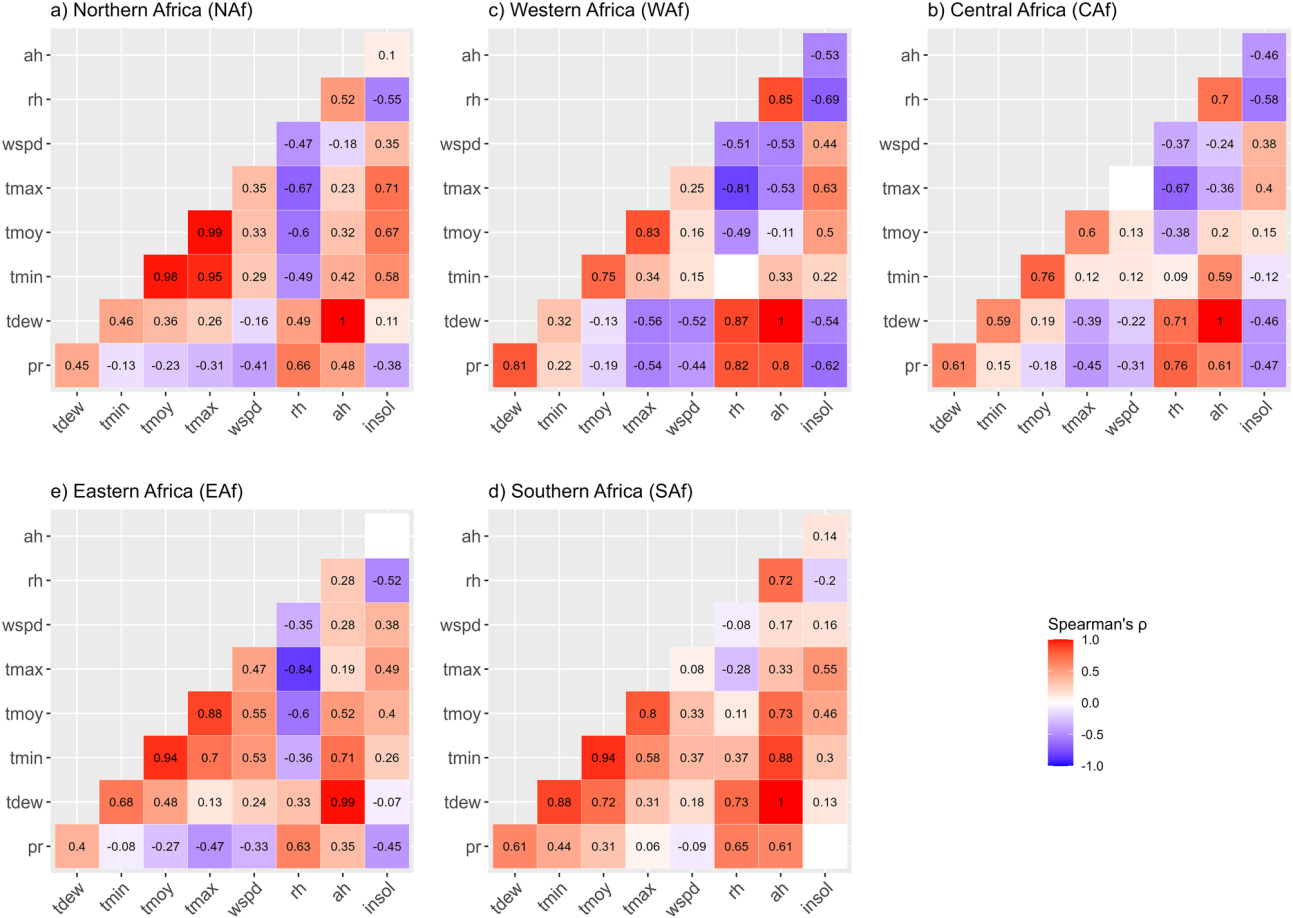
Correlations between climate variables according to regions. Correlations are evaluated using the non-parametric Spearman’s $\uprho$ correlation coefficient. Blank cells correspond to non-significant values (at $\mathrm{\alpha }$ = 5% significance level).

The correlation between climate variables is presented in Fig. 3, region-wise. Dew point temperature (*tdew*)*, tmax, tmin* and *tmoy* variables are positively associated in NAf, EAf and SAf regions, whereas *tdew* appears to be negatively associated with *tmoy* and *tmax* in the WAf region and only with *tmax* in the CAf region. Also, *wspd* is always negatively correlated with *tdew* and *pr* in NAf, WAf and CAf regions, negatively correlated with *pr* in EAf and SAf, but positively associated with *tdew* in EAf and SAf regions. *ah* appears to be positively associated with all climate variables in EAf and SAf regions, but shows negative association with *wspd* (in NAf region), *wspd, tmax* and *tmoy* (in WAf region), *wspd* and *tmax* (in CAf region). In all regions, however, *rh* and *ah* variables are positively associated with *tdew* and *pr*.

**Figure 4:**
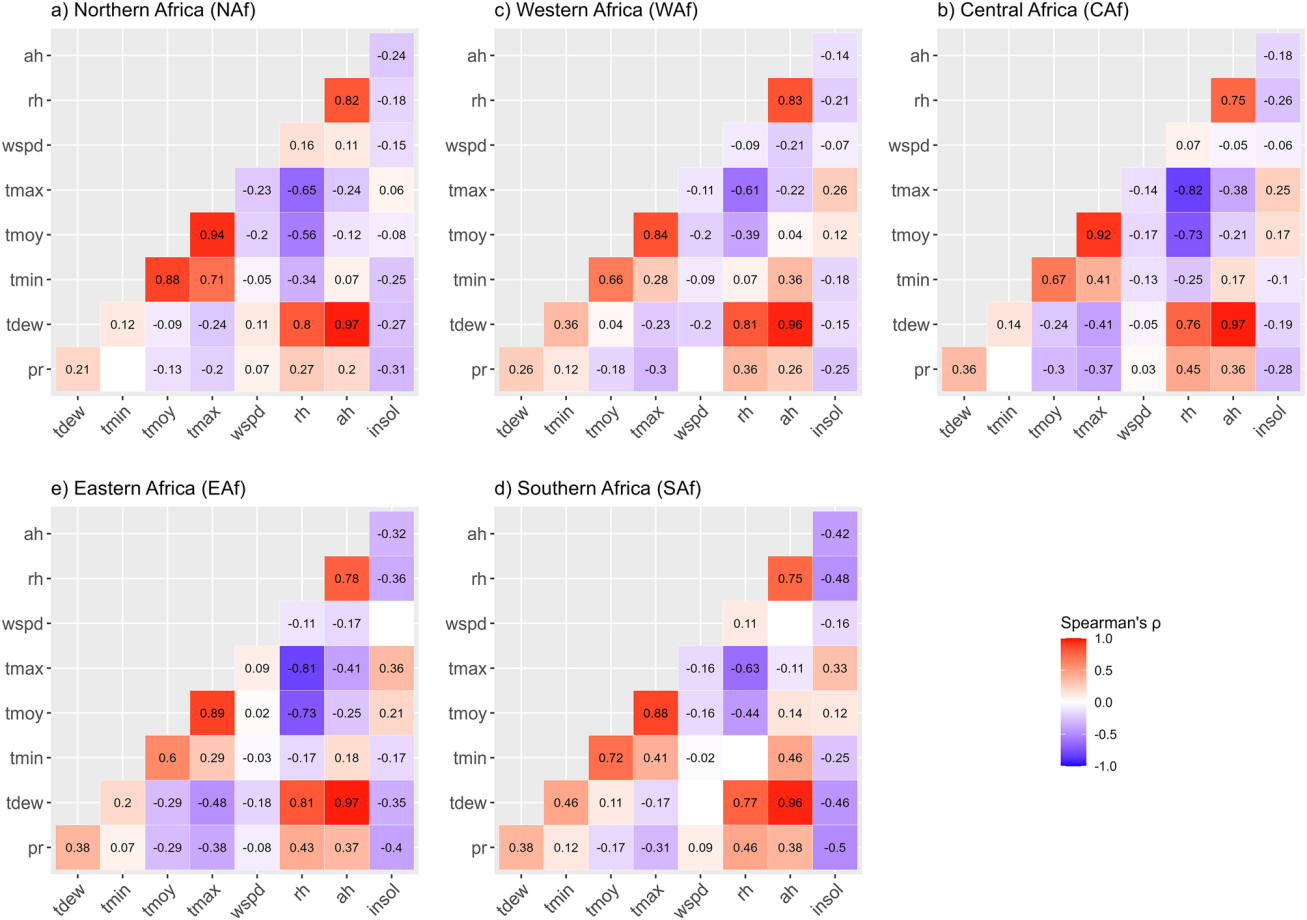
Correlations between climate anomalies (2020–2022) according to regions. Anomalies are estimated from climate normals over the period 2000–2019 (20 years). Correlations are evaluated using the non-parametric Spearman’s $\uprho$ correlation coefficient. Blank cells correspond to non-significant values (at $\mathrm{\alpha }$ = 5% significance level).

These inter-correlations shed light that climate variables show different forms of associations depending on the region. However, these correlations are influenced by seasonal variations (forced by the Earth’s rotation around the Sun), and therefore, do not consider the unique properties of intra-annual variability of the considered climate variable over the period 2020–2022. In this regard, we complement the previous analysis with the assessment of correlations on anomalies of climate variables, estimated from climate normals (over the period 2000–2019, i.e. 20 years) and presented in Fig. 4.

The analysis of correlations between anomalies reveals similar patterns across all regions. The highest positively correlated sets are *tmoy*, *tmin* and *tmax* (especially in NAf, WAf and SAf regions), and also *rh*, *ah* and *tdew* (at similar levels in all regions). A moderately positive association is observed between *insol*, *tmoy* and *tmax* in all regions except NAf. A moderately negative association is reported between *tmax*-*tmoy* and *rh*-*ah* pairs in almost all regions, and also between *rh*-*ah* pair and *insol*. These observations reveal consistent patterns of relationship between the climate variables across the different regions in Africa, despite the seasonal patterns which can be specific to each region^43^.

### Effect of meteorological factors on COVID-19 cumulative cases

#### Significant meteorological factors affecting COVID-19 transmission

The fitting of a *RegARIMA* model with meteorological factors as covariates on the COVID-19 time series of cumulative cases results in 156 candidate models in which the climate variable included is significant (Wald *t*-test on coefficients—p-value < 5%) and the residuals are similar to white noise (Ljung-Box Q test, p-value < 5%). However, within this total, some meteorological factors often show multiple significant lags for the same country. Most of the time, these multiple significant lags are not consecutive, but separated by lags without significance. This could be further explained by the recurrence time scale, as shown by the plots of the Autocorrelation Function (ACF) provided as a Supplementary Material (pages 10–63). There is also no apparent direct relationship between the number of significant lags and the decorrelation time scale.

To further filter the potential ‘*true*’ models, the ‘*duplicate*’ models are filtered to retain the model showing the highest association (i.e. the absolute largest Spearman’s $\rho$ correlation coefficient) with COVID-19. Therefore, a definitive total of 59 models are finally considered for further analysis. The complete list of these models is presented as a Supplementary Material (Table S4).

Overall, it appears that in 32 African countries (out of 54), at least one of the 9 meteorological factors considered in this study appears to be significant in COVID-19 cumulative cases. Interestingly, some countries were found to have the largest numbers of climate variables significantly related to COVID-19 cumulative cases. These countries include Côte d’Ivoire (CIV: *ah tdew, tmoy, wspd*) and Seychelles (SYC: *tdew, tmax, tmin, tmoy*), closely followed by Gabon (GAB), Guinea-Bissau (GNB), Liberia (LBR), South Sudan (SSD) and Togo (TGO).

The meteorological factors most often occurring are *tdew* (in 10 countries), followed by *rh* and *ah* (both in 9 countries). They are followed by *insol* and *tmoy* (both in 8 countries). Variables such as *pr, tmin* and *tmax* are both reported as significant in 4 countries and *wspd* in 3 countries.

**Figure 5:**
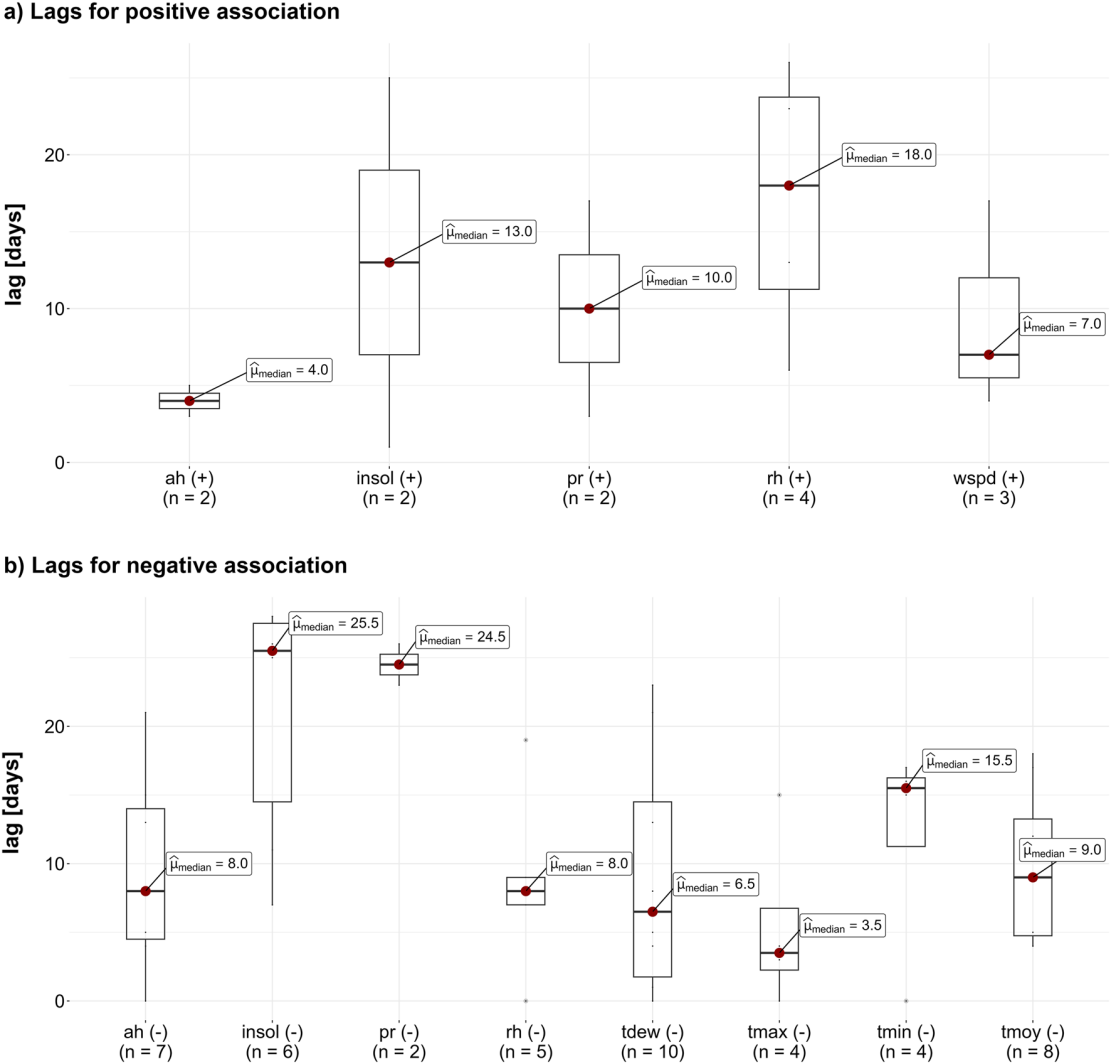
Distribution of lags according to positive and negative associations between meteorological variables and COVID-19 cumulative cases. (**a**) Distribution of lags for positive association. (**b**) Distribution of lags for negative association. The red dots show the median value over the distribution box plots, indicated in days. The number of values in each box plot is shown in parenthesis under the axis label.

Figure 5 provides a visual comparison of the distribution of the significant lags between climate variables and the COVID-19 cumulative cases across the 59 *RegARIMA* models retained while separating positive and negative association.

Some variables show a positive delayed association with COVID-19 (Fig. 5a), meaning that an increase in such variables is associated with an increase in COVID-19 cases. Such variables include *rh* and *insol* (median lags of 18.0 and 13.0 days respectively), followed by *pr* (median lag of 10.0 days), *wspd* (median lag of 7.0 days) and finally *ah* (median lag of 4.0 days).

The majority of the climate variables considered in this study show a negative delayed association with COVID-19 (Fig. 5b), meaning that an increase in these variables is likely related to a decrease in COVID-19 cases. *insol* and *pr* have the highest negative association lags (medians of 25.5 and 24.5 days respectively), followed by *tmin* (median lags of 15.5 days), then *tmoy*, *ah*, *rh* and *tdew* at a similar level (median lags of 9.0, 8.0, 8.0 and 6.5 days respectively). *tmax* shows the shorter lag in terms of negative association (median of 3.5 days).

It should be noted that some variables such as *ah*, *insol*, *pr* and *rh* show both positive and negative associations, but in different countries. However, the negative associations are less dominant, in comparison with the positive ones.

#### Spatial distribution of association of climate variables to COVID-19

**Figure 6:**
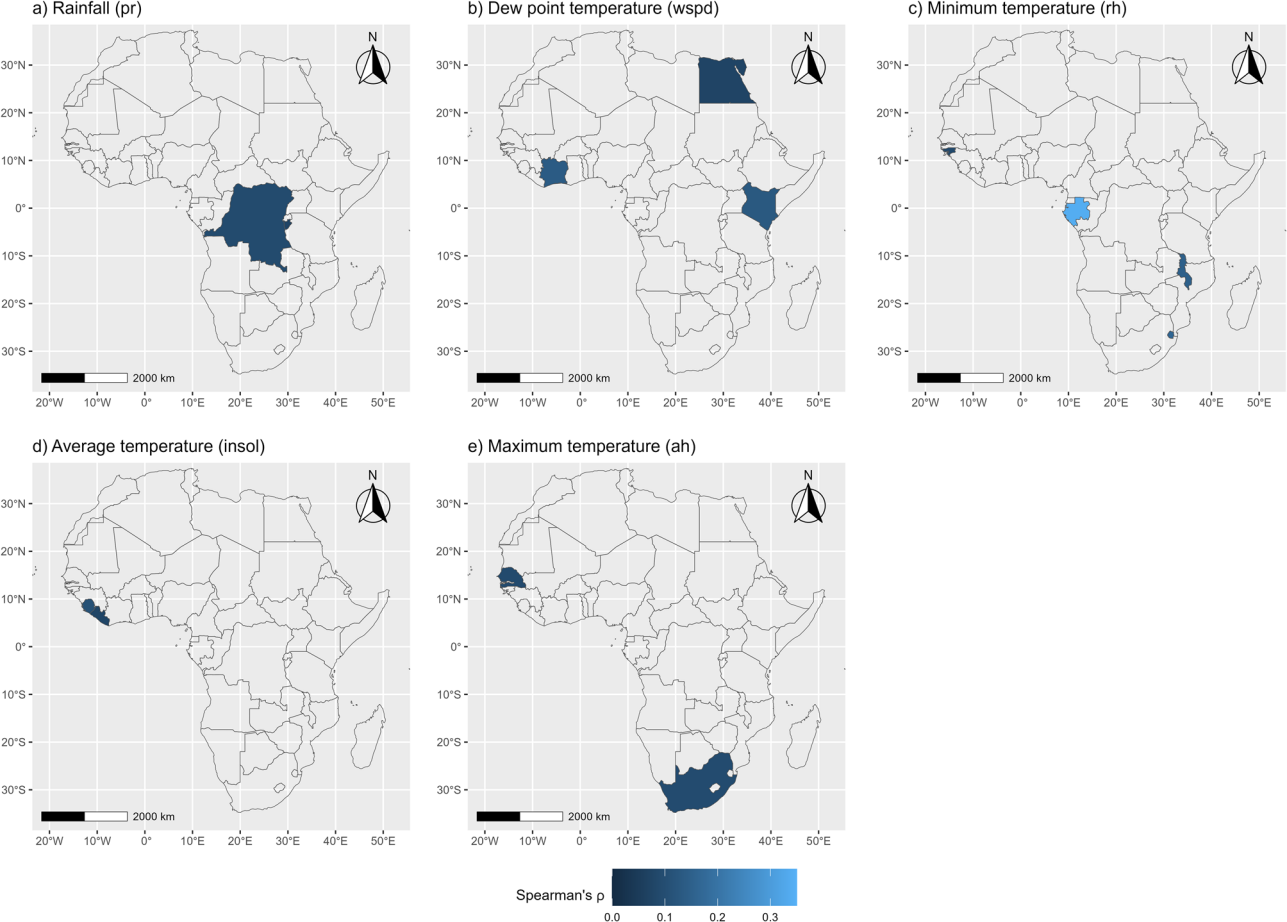
Countries showing a positive association between meteorological factors and COVID-19 cumulative cases. Correlation is estimated using the non-parametric Spearman’s $\uprho$ coefficient. Only significant values (at $\mathrm{\alpha }$ = 5% significance level) are presented. The maps produced using the *ggmap*^40^ and *ggplot2*^41^ visualisation libraries under the R programming language^42^.

The spatial distribution of countries where a positive association between a meteorological variable and COVID-19 is detected is shown in Fig. 6. The analysis shows that rainfall is positively associated with COVID-19 cases in Congo Kinshasa (COD) and Burundi (BDI). Relative humidity (*rh*) is reported significant for Gabon (GAB) in CAf, Guinea-Bissau (GNB) in WAf, but also Eswatini (SWZ) and Malawi (MWI) in SAf. Solar radiation (*insol*) is significant for Sierra Leone (SLE) and Liberia (LBR), while absolute humidity (*ah*) appears significant for Senegal (SEN) and South Africa (ZAF). Wind speed (*wspd*) is reported for Côte d’Ivoire (CIV), Kenya (KEN) and Egypt (EGY), which are mostly coastal countries.

**Figure 7:**
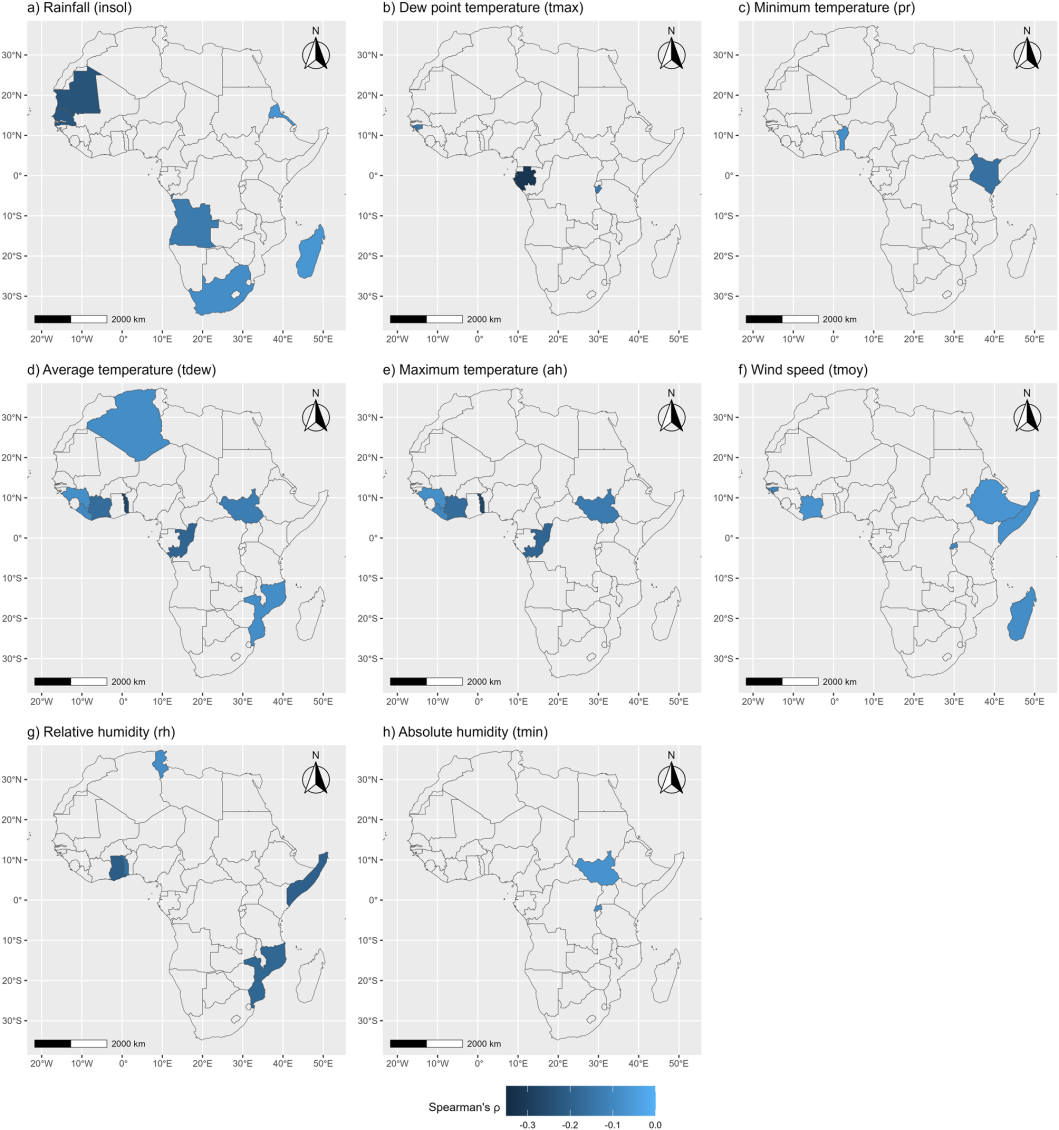
Countries showing a negative association between meteorological factors and COVID-19 cumulative cases. Correlation is estimated using the non-parametric Spearman’s $\uprho$ coefficient. Only significant values (at $\mathrm{\alpha }$ = 5% significance level) are presented. The maps produced using the *ggmap*^40^ and *ggplot2*^41^ visualisation libraries under the R programming language^42^.

The spatial distributions of countries where a negative association between meteorological variables and COVID-19 is observed are presented in Fig. 7. In this case, dew point temperature (*tdew*) is reported significant for countries mostly in WAf, EAf and SAf (to a lesser extent). Average temperature (*tmoy*) is also negatively associated with COVID-19 in mostly EAf countries and also the WAf region. Absolute (*ah*) and relative (*rh*) humidity are found significant mostly in WAf and EAf, while maximum (*tmax*) and minimum (*tmin*) temperature are reported significant mostly in the EAf region.

## Discussion

In this study, the potential effect of meteorological factors on COVID-19 transmission is evaluated in the context of African countries. The global pandemic figures show that Africa as a whole remains largely less affected by COVID-19 in comparison to Europe, America and Asia. Previous studies have suggested that this apparent resilience from Africa can be explained by the youthfulness of its population (median age of 18 years^8^). Also, some other factors include the low rate of urbanization, the reduced mobility rate due to the limited means of swift transportation and the low volume of international transit with other continents^9,26^. More recent studies even suggested the possibility of immune formation endemic to developing countries^44^, where interestingly the previous occurrence of diseases such as malaria, and tuberculosis have been associated with a lower prevalence of COVID-19^9,45,46^.

The role of meteorological conditions of COVID-19 has been investigated in previous research^9,13,18,19,24,47,48^. In this study, the findings regarding the specific role of each meteorological factor on COVID-19 transmission are not necessarily consistent across all the countries considered, in line with previous research^12^. Yet, some key signatures are emerging. First, a significant association with at least one climate variable has been detected in 32 countries (out of 54). The prominent factor appears to be dewpoint temperature (in 10 countries), for which negative associations have been observed in this study but also reported in the literature^49,50^. Relative and absolute humidity closely follow, (both occurring in 9 countries), in line with earlier studies^12,19,51,52^. To a similar extent, solar radiation and average temperature are both reported in 8 countries in this study but also in previous research, for both positive^53,54^ and negative^52^ associations. Finally, to a lesser extent, variables such as rainfall, minimum and maximum temperature appear as the less often occurring factors (in 4 countries)^55^, and wind speed (in 3 countries), as already observed in previous research^3,56^.

Some previous studies also highlighted the major role of average temperature and relative humidity on COVID-19 transmission, which was primarily considered an airborne disease whose transmission might be amplified in humid environments, with low temperatures, less solar radiation and higher wind speed^57,58^. Yet, an interesting finding highlighted in this study is the fact that some of the meteorological factors show significant positive and negative associations with COVID-19, in line with the findings of previous studies^12,59^. In this study, such variables include rainfall, relative and absolute humidity and solar radiation. In the case of solar radiation, previous research suggests that increased solar radiation reduces the likelihood of airborne persistence of the virus in outdoor environments, decreasing its transmission^13^; Yet, the positive association of high solar radiation could be explained by the fact that high temperature increases human mobility, resulting in people gathering outside, breaking lockdown measures, therefore increasing the likelihood of transmission^60^. As for rainfall, relative and humidity, regarding the negative association highlighted in this study, previous research suggests that it can be partly explained by the fact that most people stay home on rainy days, hence decreasing the likelihood of the spread of the disease^61^. Also, rainfall causes the washout of aerosols, which is known to decrease the residence time of the virus in the atmosphere^12^. Other studies however explain that at specific ranges of humidity, the virus can remain active for up to 4–5 days on outdoor surfaces, increasing the likelihood of transmission and resulting in positive association to COVID-19^62,63^. Overall, the findings in this study shed light on the complex and contrasting effect of meteorological factors on COVID-19 prevalence in Africa, but also that the climate sensitivity of the disease varies greatly in space and is not consistent across all countries. Also, it should be acknowledged that other major factors, such as socio-economic features and immunity of the populations might have a stronger influence on the spread of the pandemic^9,62^.

This study highlighted major findings regarding the pandemic spread in Africa: first, the relationship between meteorological conditions and COVID-19 and their time-dependence structure have been explored. The substantial geographical differences in the prevalence have also been highlighted, especially towards the end of the study period, which resulted in a clear clustering in the severity of the disease. However, there are also some limitations which should be acknowledged. First, we should be well aware that all models are as perfect as the data used. Especially in the case of African countries, COVID-19 daily reports tend to be fraught with inaccuracies, especially when testing policies are different or not heavily promoted. Second, the analysis framework used in this study relies on time-series-based modelling, in which case time-dependency structural behaviour can sometimes result in spurious correlations, which should often be treated with caution. It should also be noted that some specific factors were not included in our analysis such as air quality due to the lack of accurate data in the case of Africa. Air quality, especially the concentration of fine particulate matter (PM2.5) has been previously highly associated with COVID-19 in previous large-scale studies^9,58^. We should also indicate that this study focused on the individual effect of climate variables on COVID-19, yet the synergistic effect of all climate variables altogether was not considered, leaving out interaction effects^55,64^. Finally, some other shortcomings are the non-consideration of socio-economic factors, healthcare system and other development country-level indicators, and population immunity, which could explain the variation in specific sensitivities of countries to COVID-19^9^. However, in our understanding, such factors are less likely to explain day-to-day variations, which is the target of this study. In this regard, our findings appear to be of importance as they shed light on the contribution of climate to the spread of the pandemic. This, in turn, might help refine further modelling and forecasting attempts, sustain the preparedness of healthcare systems and foster informed management policies.

## Material and methods

### Ethical statement

The data used in this study, regarding both COVID-19, stringency index and meteorological factors, are all available from public databases. Therefore, an ethical review was not required.

### Sources of data used in this study

In this study, COVID-19 cumulative confirmed cases data were gathered for the study period covering January 1, 2020, to September 30, 2022. The data were automatically collected at the daily timestep and for each African country (54 countries in total), using the R package *COVID-19*^6^. The time series were treated for inconsistencies, such as decreasing values in cumulative confirmed cases from 1 day to the next (occurring for example for Benin—BEN).

The climate variables considered in this study for potential significant association with COVID-19 prevalence are cumulative rainfall (*pr*), dewpoint temperature (*tdew*), minimum (*tmin*), mean (*tmoy*) and maximum (*tmax*) temperatures, wind speed (*wspd*), relative humidity (*rh*), absolute humidity (*ah*) and solar radiation (*insol*), both at the daily timescale. The data for these variables were collected from the so-called “*Modern-Era Retrospective Analysis for Research and Applications*” (MERRA-2^65^), which is a global climate reanalysis (at the spatial resolution of 0.625° × 0.5°), accessible through the NASA POWER Data Access Viewer (https://power.larc.nasa.gov/data-access-viewer/). The data were automatically collected using the *nasapower* R package^66^. The time series were also treated for missing values using spline interpolation. The daily values of absolute humidity (*ah*) were calculated from the Clausius Clapeyron approximation, as shown in Eq. (1)^9^:

1 $$ah=\left(6.112\times \frac{17.67\times tmoy}{\mathit{exp}(tmoy+243.5)}\times rh\times 2.1674\right)/(tmoy+273.15)$$

where $ah$ is the absolute humidity (%), $tmoy$ is the average temperature (°C), $rh$ is the air relative humidity (%) and $\mathit{exp}$ is the natural base logarithm.

Over the study period, various and different policy responses to COVID-19 have been adopted by countries to contain and mitigate the spread of the pandemic. In this study, as the analysis of the potential effect of climate variables is carried out on a cross-sectional basis, it is critical to isolate the potential effect of such different policy responses from that of the climate variables considered. Therefore, a stringency index (*si*), defined as a composite mean score of nine pandemic response measures (school closures, workplace closures, cancellation of public events, restrictions on public gatherings, closures of public transport, stay-at-home requirements, public information campaigns, restrictions on internal movements and international travel controls) is systematically considered as a potential covariate for each country’s COVID-19 confirmed cases time series. The *si* index takes a value between 0 and 100, with a higher score indicating a stricter mitigation response^67^. The *si* time series data were collected from Ritchie et al.^68^.

### Inter-relations and spatial patterns in climate variables and COVID-19 prevalence

Climate variables often exhibit medium to long-term associations depending on the local climate system. In this study, the potential relationships between the 9 climate variables (*pr, tdew, tmin, tmoy, tmax, wspd, rh, ah* and *insol*) are assessed through the non-parametric Spearman’s rank correlation test (at $\alpha$ = 5% significance level). To account for regional contrasts, the correlations were evaluated depending on regions, i.e. North Africa (NAf), West Africa (WAf), Central Africa (CAf), East Africa (EAf) and Southern Africa (SAf). Also, the average spatial distribution of these variables is presented through choropleths maps.

In addition, because of seasonal cycles, intra-annual weather variability can be typically masked, blurring the inter-relationships analysed. To address this issue, we complement the above correlation analysis with the examination of correlations between the weather variables anomalies, still using the non-parametric Spearman’s rank correlation (at $\alpha$ = % significance level). The anomalies are evaluated by subtracting the weather variables values from the day-by-day country climatological mean (or daily climate normal) evaluated over the period 2000–2019 (i.e., 20 years). The daily climate normals are calculated by averaging the daily time series for each meteorological variable for each day of the year (1, 2, …, 365). For leap years, the date of February 29 (i.e., the 60th day of the year) was skipped to maintain the climate normals time series to a fixed length of 365 values, for ease of computation. The data were collected from MERRA-2 climate reanalysis through the use of the R *nasapower* package^66^.

Furthermore, to evaluate the spatial clustering of the pandemic spread at the level of the continent, the spatial autocorrelation in cumulative cases (per million people) at different time points selected within the study period is assessed through Global Moran’s *I*^69^. Moran’s *I* statistic is a common indicator of global spatial autocorrelation, defined as a cross-product statistic between a variable and its spatial lag, with the variable expressed in deviations from its mean value, as in Eq. (2):

2 $$I=\frac{n}{{S}_{0}}\frac{{\sum }_{i}{\sum }_{j}{\omega }_{ij}({x}_{i}-\overline{x })({x}_{j}-\overline{x })}{{\sum }_{i}{\left({x}_{i}-\overline{x }\right)}^{2}}$$

where ${x}_{i}$ and ${x}_{j}$ is a pair of distinct observations at locations $i,j$ (respectively), $\overline{x }$ is the mean of the variable $x$, ${\omega }_{ij}$ are the elements of spatial weights matrix, ${S}_{0}={\sum }_{i}{\sum }_{j}{\omega }_{ij}$ is the sum of all the weights and $n$ is the number of observations. The expected value of *I* under the null hypothesis (no spatial autocorrelation) is given by ${I}_{0}=-1/(n-1)$^70^. The evaluation of Moran’s *I* was carried out in this study through the *ape* R package^71^ using the inverse Euclidean distance on all pairs of countries’ centroid coordinates to compute the spatial matrix weights.

### Potential association of climate variables to COVID-19 transmission

**Figure 8:**
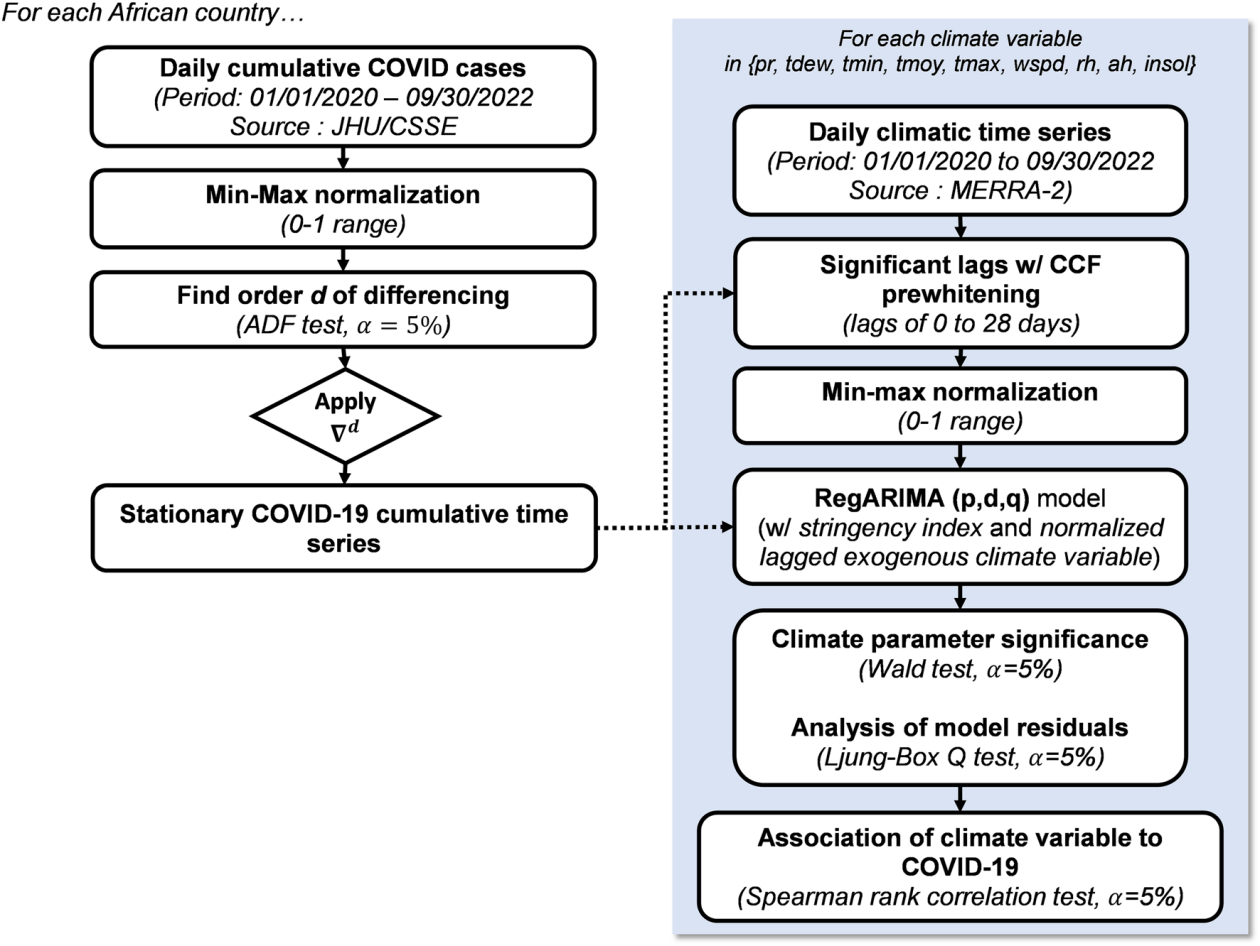
Methodology flowchart of the study.

The general workflow of the methodology used to detect the potential association between meteorological variables and COVID-19 is presented in Fig. 8. The following sections describe in detail the steps of the analysis.

#### Pre-processing COVID-19 cumulative cases time series

The time series of COVID-19 cumulative confirmed cases for each country are normalized through the *min–max* method, in the 0–1 range (Eq. 3). This operation ensures cross-comparison of the results (between countries), especially when estimating the magnitude of the effect of the climate variables analysed.

3 $$x=\frac{X-\mathrm{min}(X)}{\mathrm{max}\left(X\right)-\mathrm{min}(X)}$$

where $x$ is the normalized time series, $X$ is the original time series, $\mathrm{min}(X$) and $\mathrm{max}(X)$ are respectively the minimum and maximum values in the $X$ time series.

#### Time series-based modelling of COVID-19 and effect of climate variables

The modelling framework used in this study is based on the *Autoregressive Integrated Moving Average* (ARIMA) model, which is a common approach used in time series modelling, resulting in the backshift notation presented in Eq. (4)^72^:

4 $${\left(1-B\right)}^{d}{Y}_{t}=\mu +\frac{\theta (B)}{\phi (B)}{\mathrm{\alpha }}_{\mathrm{t}}$$

where $t$ indexes time (in days), $\mu$ is the mean term, $B$ is the backshift operator (that is $B{Y}_{t}={Y}_{t-1}$), $\phi (B)$ is the autoregressive operator represented as a polynomial in the backshift operator ($\phi \left(B\right)=1-{\phi }_{1}B-\dots -{\phi }_{p}{B}^{p}$), $\theta (B)$ is the moving average operator represented as a polynomial in the backshift operator ($\theta \left(B\right)=1-{\theta }_{1}B-\dots -{\theta }_{q}{B}^{q}$) and ${\alpha }_{t}$ is the independent random error term. The ARIMA model is defined through three non-negative parameters *p, d q*, respectively defined as the number of time lags, the degree of differencing and the order of the moving average model. The order of differencing *d* is obtained through successive applications of the Augmented Dickey-Fuller (ADF) test (at $\alpha$ = 5% significance level) to increasing levels of differencing applied to the original time series. However, in this study, a minimum value of *d* = *1* is fixed to ensure that the modelled time series are typically daily new cases, instead of the raw cumulative values. The time series obtained after differencing is considered stationary. The procedure was carried out using the *ndiffs* command from the R forecast package^72^.

The potential effect of climate variables on COVID-19 confirmed cases is assessed through a variation of the *ARIMA (p,d,q)* model, which includes exogenous explanatory variables. Previous studies indicate that the effect of climate is not necessarily immediate on COVID-19 transmission, but can typically be lagged by up to 28 days^73^. Therefore, in this study, for each of the 9 climate variables considered (*pr, tdew, tmin, tmoy, tmax, wspd, rh, ah* and *insol*), the time series data collected over the study period is initially screened to filter significant lags (between 0 to 28 days) through pre-whitening. Pre-whitening helps in evaluating at which specific lag a covariate (independent variable) affects a dependent variable^74^. The steps in the pre-whitening procedure include:

i. fitting an ordinary *ARIMA (p,d,q)* model to the dependent variable time series and saving the residuals (say $re{s}_{Y}$) ;
ii. fitting the same model to the covariate time series and saving the residuals (say $re{s}_{X}$);
iii. analyzing the cross-correlation function between the residuals $re{s}_{X}$ and $re{s}_{Y}$ and identify significant lags (using nonparametric Spearman’s rank correlation at $\alpha$ = 5% significance level). These lags are defined as the lags for which cross-correlation exceeds the 95% confidence interval limits under the assumption that the null hypothesis (H_0_: no correlation) is true. Here, to account for the length of the time series, which could deflate the reported p-value^75^ and to provide better estimates (and confidence interval around these estimates^76^), the correlation was assessed through screening of the distribution of 1000 sieve random bootstrap simulations, carried out using the ‘*ccf_boot’* command in the R *funtimes* package^77^.

Another major advantage in working with the residuals (from the pre-whitening procedure) is that such residuals are assumed to be independent, while the original meteorological variables are unlikely, since weather systems could persist for many days, therefore reducing the number of degrees of freedom of the time series. This effect is further amplified by seasonal fluctuations, which should be thoroughly addressed when assessing for statistical significance to avoid the detection of spurious correlations^78,79^.

Also, in this study, only strictly negative lags were retained, i.e. climate leads to COVID-19 cases, but not the other way around. The significant lags identified are applied by shifting the climate variable time series considered, which is further normalized in the 0–1 range through the *min–max* method (see Eq. 3). This ensures cross-comparison of the results, especially considering that climate variable values are defined on different ranges.

Further, a regression model with *ARIMA (p,d,q)* errors is developed using simultaneously the stringency *si* index time series (also *min–max* normalized in the 0–1 range) and the considered climate covariate. The regression model obtained, hereafter referred to as *RegARIMA (p,d,q)*, is defined as in the backshift notation given in Eq. (5):

5 $${y}_{t}=\beta {x}_{t}+\frac{\theta (B)}{{\left(1-B\right)}^{d}\phi (B)}{z}_{\mathrm{t}}$$

where ${y}_{t}$ is the response variable (confirmed cases), $\beta$ is a coefficient matrix, ${x}_{t}$ is the covariate matrix formed by the stringency index and the climate variable considered and ${z}_{t}$ is a white noise process. The *RegARIMA (p,d,q)* model parameters *p* and *q* are obtained through maximum likelihood fitting using the *auto.arima* function provided with the *forecast* R package^72^. The Bayesian Information Criterion (BIC) is used to select the optimal model, defined as the one minimizing the BIC^12,72^. The definitive optimal and meaningful models are retained based on the following conditions:

i. *the model residuals are typically similar to white noise*: this condition further ensures that the model has captured the maximum variance in the dependent variable (i.e., the COVID-19 prevalence data). This is assessed through the application of the Ljung-Box Q test (at $\alpha$ = 5% significance level)^72,80^.
ii. *the climate variable coefficient is significant in the model*: this condition is assessed through the Wald t-test of coefficients at $\alpha$ = 5% significance level. This statistical test is applied with the *lmtest* R package^81^.
iii. *the climate variable with its actual lag is significantly correlated to COVID-19 cumulative cases*: this condition is assessed using the non-parametric Spearman rank correlation test at $\alpha$ = 5% significance level. The correlation coefficient $\rho$ is an indication of the effect size of the relationship, and assesses the association both in terms of magnitude and direction (positive or negative). A positive (negative) association implies that as the climate variable increases, the number of reported cases increases (decreases).

### Supplementary Information

Supplementary Information.

## Data Availability

All the data used in this study are publicly available from various sources included in the manuscript.

## Code availability

The code used to analyse the data and prepare the figures is available at Github (https://github.com/Yonaba/covid19_climate_ts_modelling).

## References

[1] Holmes EC (2021). The origins of SARS-CoV-2: A critical review. Cell.

[2] Green MS (2020). Did the hesitancy in declaring COVID-19 a pandemic reflect a need to redefine the term?. Lancet.

[3] d’Albis H, Coulibaly D, Roumagnac A, de Carvalho Filho E, Bertrand R (2021). Quantification of the effects of climatic conditions on French hospital admissions and deaths induced by SARS-CoV-2. Sci. Rep..

[4] Miller RK, Hui I (2022). Impact of short school closures (1–5 days) on overall academic performance of schools in California. Sci. Rep..

[5] Dong E, Du H, Gardner L (2020). An interactive web-based dashboard to track COVID-19 in real time. Lancet Infect. Dis..

[6] Guidotti E, Ardia D (2020). COVID-19 data hub. JOSS.

[7] Lawal Y (2021). Africa’s low COVID-19 mortality rate: A paradox?. Int. J. Infect. Dis..

[8] Lulbadda KT, Kobbekaduwa D, Guruge ML (2021). The impact of temperature, population size and median age on COVID-19 (SARS-CoV-2) outbreak. Clin. Epidemiol. Glob. Health.

[9] Sidibé ML (2022). Understanding the COVID-19 pandemic prevalence in Africa through optimal feature selection and clustering: Evidence from a statistical perspective. Environ. Dev. Sustain..

[10] Randazzo W, Cuevas-Ferrando E, Sanjuán R, Domingo-Calap P, Sánchez G (2020). Metropolitan wastewater analysis for COVID-19 epidemiological surveillance. Int. J. Hyg. Environ. Health.

[11] Iqbal A, Haq W, Mahmood T, Raza SH (2022). Effect of meteorological factors on the COVID-19 cases: A case study related to three major cities of the Kingdom of Saudi Arabia. Environ. Sci. Pollut. Res..

[12] Hossain MdS, Ahmed S, Uddin MdJ (2021). Impact of weather on COVID-19 transmission in south Asian countries: An application of the ARIMAX model. Sci. Total Environ..

[13] Chen S (2021). Climate and the spread of COVID-19. Sci. Rep..

[14] Sahoo MM (2021). Significance between air pollutants, meteorological factors, and COVID-19 infections: Probable evidences in India. Environ. Sci. Pollut. Res..

[15] Jefferson, T., Spencer, E., Plüddemann, A., Roberts, N. & Heneghan, C. Analysis of the transmission dynamics of COVID-19: an Open Evidence Review. https://www.cebm.net/evidence-synthesis/transmission-dynamics-of-covid-19 (2021).

[16] Bashir MF (2020). Correlation between climate indicators and COVID-19 pandemic in New York, USA. Sci. Total Environ..

[17] Zhuang Z (2020). Preliminary estimation of the novel coronavirus disease (COVID-19) cases in Iran: A modelling analysis based on overseas cases and air travel data. Int. J. Infect. Dis..

[18] Kerr GH, Badr HS, Gardner LM, Perez-Saez J, Zaitchik BF (2021). Associations between meteorology and COVID-19 in early studies: Inconsistencies, uncertainties, and recommendations. One Health.

[19] Wang (2021). Impact of temperature and relative humidity on the transmission of COVID-19: A modelling study in China and the United States. BMJ Open.

[20] Jüni P (2020). Impact of climate and public health interventions on the COVID-19 pandemic: A prospective cohort study. CMAJ.

[21] Ganslmeier M, Furceri D, Ostry JD (2021). The impact of weather on COVID-19 pandemic. Sci. Rep..

[22] Rodó X, San-José A, Kirchgatter K, López L (2021). Changing climate and the COVID-19 pandemic: More than just heads or tails. Nat. Med..

[23] Briz-Redón Á, Serrano-Aroca Á (2020). The effect of climate on the spread of the COVID-19 pandemic: A review of findings, and statistical and modelling techniques. Prog. Phys. Geogr. Earth Environ..

[24] Liu J (2020). Impact of meteorological factors on the COVID-19 transmission: A multi-city study in China. Sci. Total Environ..

[25] Mohamadou Y, Halidou A, Kapen PT (2020). A review of mathematical modeling, artificial intelligence and datasets used in the study, prediction and management of COVID-19. Appl. Intell..

[26] Zongo P, Zorom M, Mophou G, Dorville R, Beaumont C (2020). A model of COVID-19 transmission to understand the effectiveness of the containment measures: Application to data from France. Epidemiol. Infect..

[27] Zhang P (2022). Usage of compartmental models in predicting COVID-19 outbreaks. AAPS J..

[28] Torrealba-Rodriguez O, Conde-Gutiérrez RA, Hernández-Javier AL (2020). Modeling and prediction of COVID-19 in Mexico applying mathematical and computational models. Chaos Solitons Fract..

[29] Conde-Gutiérrez RA, Colorado D, Hernández-Bautista SL (2021). Comparison of an artificial neural network and Gompertz model for predicting the dynamics of deaths from COVID-19 in México. Nonlinear Dyn..

[30] Ilie O-D (2020). Forecasting the spreading of COVID-19 across nine countries from Europe, Asia, and the American continents using the ARIMA models. Microorganisms.

[31] Naing, C., Ni, H., Aung, H. H., Chan, E. & Mak, J. W. *The influence of climate factors on COVID-19 transmission in Malaysia: An autoregressive integrated moving average (ARIMA) model*. 10.1101/2020.08.14.20175372 (2020).

[32] Sahai AK, Rath N, Sood V, Singh MP (2020). ARIMA modelling & forecasting of COVID-19 in top five affected countries. Diabetes Metab. Syndr. Clin. Res. Rev..

[33] Adekunle IA, Tella SA, Oyesiku KO, Oseni IO (2020). Spatio-temporal analysis of meteorological factors in abating the spread of COVID-19 in Africa. Heliyon.

[34] Martinez-Alvarez M (2020). COVID-19 pandemic in west Africa. Lancet Glob. Health.

[35] Ssentongo, P. *et al. Tracking and predicting the African COVID-19 pandemic*. 10.1101/2020.11.13.20231241 (2020).

[36] Ai H, Nie R, Wang X (2022). Evaluation of the effects of meteorological factors on COVID-19 prevalence by the distributed lag nonlinear model. J. Transl. Med..

[37] Gwenzi W, Rzymski P (2021). When silence goes viral, Africa sneezes! A perspective on Africa’s subdued research response to COVID-19 and a call for local scientific evidence. Environ. Res..

[38] ACSS. Africa’s varied COVID landscapes. *Africa Center for Strategic Studies* (2020).

[39] ACSS. Mapping COVID-19 risk factors. *Africa Center for Strategic Studies* (2020).

[40] Kahle, D. & Wickham, H. ggmap: Spatial Visualization with ggplot2. (2016).

[41] Wickham, H. ggplot2: Elegant Graphics for Data Analysis. (2016).

[42] R Core Team. *R: A Language and Environment for Statistical Computing*. (R Foundation for Statistical Computing, 2022).

[43] Maidment RI (2014). The 30 year TAMSAT African rainfall climatology and time series (TARCAT) data set. J. Geophys. Res. Atmos..

[44] Chatterjee, B., Karandikar, R. L. & Mande, S. C. *Paradoxical Case Fatality Rate dichotomy of Covid-19 among rich and poor nations points to the “hygiene hypothesis”*. 10.1101/2020.07.31.20165696 (2020).

[45] Iesa MAM (2020). SARS-CoV-2 and *Plasmodium falciparum* common immunodominant regions may explain low COVID-19 incidence in the malaria-endemic belt. New Microbes New Infect..

[46] Anjorin AA (2021). Comorbidities and the COVID-19 pandemic dynamics in Africa. Trop. Med. Int. Health.

[47] Baker RE, Yang W, Vecchi GA, Metcalf CJE, Grenfell BT (2020). Susceptible supply limits the role of climate in the early SARS-CoV-2 pandemic. Science.

[48] Braiman M (2020). Latitude dependence of the COVID-19 mortality rate—A possible relationship to vitamin D deficiency?. SSRN Electron. J..

[49] Selcuk M, Gormus S, Guven M (2021). Impact of weather parameters and population density on the COVID-19 transmission: Evidence from 81 provinces of Turkey. Earth Syst. Environ..

[50] Neisi A (2023). Association of the corona virus (Covid-19) epidemic with environmental risk factors. Environ. Sci. Pollut. Res..

[51] Ganegoda NC, Wijaya KP, Amadi M, Erandi KKWH, Aldila D (2021). Interrelationship between daily COVID-19 cases and average temperature as well as relative humidity in Germany. Sci. Rep..

[52] Islam MdM, Noor FM (2022). Correlation between COVID-19 and weather variables: A meta-analysis. Heliyon.

[53] Hachim MY (2021). Higher temperatures, higher solar radiation, and less humidity is associated with poor clinical and laboratory outcomes in COVID-19 patients. Front. Public Health.

[54] Liu M (2022). Association between temperature and COVID-19 transmission in 153 countries. Environ. Sci. Pollut. Res..

[55] Moazeni M, Rahimi M, Ebrahimi A (2023). What are the effects of climate variables on COVID-19 pandemic? A systematic review and current update. Adv. Biomed. Res..

[56] Rendana M (2020). Impact of the wind conditions on COVID-19 pandemic: A new insight for direction of the spread of the virus. Urban Clim..

[57] Barcellos DDS, Fernandes GMK, de Souza FT (2021). Data based model for predicting COVID-19 morbidity and mortality in metropolis. Sci. Rep..

[58] Lim YK, Kweon OJ, Kim HR, Kim T-H, Lee M-K (2021). The impact of environmental variables on the spread of COVID-19 in the Republic of Korea. Sci. Rep..

[59] McClymont H, Hu W (2021). Weather variability and COVID-19 transmission: A review of recent research. IJERPH.

[60] Damette O, Mathonnat C, Goutte S (2021). Meteorological factors against COVID-19 and the role of human mobility. PLoS ONE.

[61] Shenoy A (2022). God is in the rain: The impact of rainfall-induced early social distancing on COVID-19 outbreaks. J. Health Econ..

[62] Doğan B, Ben Jebli M, Shahzad K, Farooq TH, Shahzad U (2020). Investigating the effects of meteorological parameters on COVID-19: Case study of New Jersey, United States. Environ. Res..

[63] Lewis D (2020). Mounting evidence suggests coronavirus is airborne—but health advice has not caught up. Nature.

[64] Yonaba R (2023). Trends, sensitivity and estimation of daily reference evapotranspiration ET0 using limited climate data: Regional focus on Burkina Faso in the West African Sahel. Theor. Appl. Climatol..

[65] Gelaro R (2017). The modern-era retrospective analysis for research and applications, version 2 (MERRA-2). J. Clim..

[66] Sparks, A. nasapower: NASA-POWER Data from R. (2021).

[67] Hale T (2021). A global panel database of pandemic policies (Oxford COVID-19 Government Response Tracker). Nat. Hum. Behav..

[68] Ritchie, H. *et al.* Coronavirus Pandemic (COVID-19). *Our World in Data* (2020).

[69] Moran PAP (1948). The interpretation of statistical maps. J. R. Stat. Soc. Ser. B Methodol..

[70] Anselin L, Fischer M, Scholten HJ, Unwin D (2019). The Moran scatterplot as an ESDA tool to assess local instability in spatial association. Spatial Analytical Perspectives on GIS.

[71] Paradis E, Schliep K (2019). ape 5.0: An environment for modern phylogenetics and evolutionary analyses in R. Bioinformatics.

[72] Hyndman RJ, Khandakar Y (2008). Automatic time series forecasting: The forecast package for *R*. J. Stat. Soft..

[73] Bañuelos Gimeno J (2022). Air pollution and meteorological variables’ effects on COVID-19 first and second waves in Spain. Int. J. Environ. Sci. Technol..

[74] Razavi S, Vogel R (2018). Prewhitening of hydroclimatic time series? Implications for inferred change and variability across time scales. J. Hydrol..

[75] Halsey LG, Curran-Everett D, Vowler SL, Drummond GB (2015). The fickle P value generates irreproducible results. Nat. Methods.

[76] Wilcox RR (2011). Introduction to Robust Estimation and Hypothesis Testing.

[77] Lyubchich, V., Gel, Y. R. & Vishwakarma, S. funtimes: Functions for Time Series Analysis. (2023).

[78] Bretherton CS, Widmann M, Dymnikov VP, Wallace JM, Bladé I (1999). The effective number of spatial degrees of freedom of a time-varying field. J. Clim..

[79] Metz W (1991). Optimal relationship of large-scale flow patterns and the barotropic feedback due to high-frequency eddies. J. Atmos. Sci..

[80] Ljung GM, Box GEP (1978). On a measure of lack of fit in time series models. Biometrika.

[81] Zeileis A, Hothorn T (2002). Diagnostic checking in regression relationships. R. News.

