# Supplementary material for: Climate and COVID-19 transmission: a cross-sectional study in Africa
Source: Sci Rep. 2023 Oct 31;13:18702. doi: 10.1038/s41598-023-46007-0 (PMC10618194; doi:10.1038/s41598-023-46007-0)

Supplementary Information

Climate and COVID-19 Transmission: A Cross-Sectional Study in Africa

Ousmane Koanda[1], Roland Yonaba[1,*], Fowe Tazen[1], Héla Karoui[1], Mohamed Lamine Sidibé[1], Babacar Lèye[1], Mamadou Diop[2], Harinaivo Anderson Andrianisa[1], Harouna Karambiri[1]

[1] *Laboratoire Eaux, Hydro-Systèmes et Agriculture (LEHSA), Institut International d'Ingénierie de l'Eau et de l'Environnement (2iE), Ouagadougou, Burkina Faso*

[2] *Laboratoire Eco-Matériaux et Habitat Durable (LEMHaD), Institut International d'Ingénierie de l'Eau et de l'Environnement (2iE), Ouagadougou, Burkina Faso*

* Corresponding author; email: ousmane.yonaba@2ie-edu.org / roland.yonaba@gmail.com

Table S1. Summary statistics of cumulative cases per region in Africa (as of 10/30/2022).

	cases (million people)					cases (per 1000.million people)				
**Regions**	**NAf**	**WAf**	**CAf**	**EAf**	**SAf**	**NAf**	**WAf**	**CAf**	**EAf**	**SAf**
**Min**	0.063	0.008	0.006	0.010	0.008	1.409	0.374	0.448	0.641	2.345
**Q1**	0.167	0.011	0.015	0.023	0.072	5.506	1.396	3.031	2.224	8.938
**Q2**	0.507	0.033	0.025	0.047	0.200	13.155	2.790	4.390	4.096	17.344
**Q3**	0.831	0.075	0.093	0.151	0.279	53.366	4.779	11.733	8.067	65.729
**Max**	1.265	0.265	0.122	0.493	4.003	95.988	111.005	27.892	475.202	207.453

Regions: Northern Africa (NAf), Western Africa (WAf), Central Africa (CAf), Eastern Africa (EAf) and Southern Africa (SAf). Min: minimum value for countries in the region. Max: maximum value for countries in the region. Q1, Q2, Q3: First, second and third quartiles.

Table S2. Correspondence table between African countries and regions

NAf (Northern Africa)		WAf (West Africa)		EAf (West Africa)		CAf (Central Africa)		SAf (Central Africa)	
**ISO Code**	**Name**	**ISO Code**	**Name**	**ISO Code**	**Name**	**ISO Code**	**Name**	**ISO Code**	**Name**
DZA	Algeria	BEN	Benin	BDI	Burundi	AGO	Angola	BWA	Botswana
EGY	Egypt	BFA	Burkina Faso	DJI	Djibouti	CMR	Cameroon	COM	Comoros
LBY	Libya	CPV	Cabo Verde	ERI	Eritrea	CAF	Central African Republic	SWZ	Eswatini
MRT	Mauritania	CIV	Cote d'Ivoire	ETH	Ethiopia	TCD	Chad	LSO	Lesotho
MAR	Morocco	GMB	Gambia	KEN	Kenya	COG	Congo (Brazzaville)	MDG	Madagascar
SDN	Sudan	GHA	Ghana	RWA	Rwanda	COD	Congo (Kinshasa)	MWI	Malawi
TUN	Tunisia	GIN	Guinea	SYC	Seychelles	GNQ	Equatorial Guinea	MUS	Mauritius
		GNB	Guinea-Bissau	SOM	Somalia	GAB	Gabon	MOZ	Mozambique
		LBR	Liberia	SSD	South Sudan	STP	Sao Tome and Principe	NAM	Namibia
		MLI	Mali	TZA	Tanzania			ZAF	South Africa
		NER	Niger	UGA	Uganda			ZMB	Zambia
		NGA	Nigeria					ZWE	Zimbabwe
		SEN	Senegal						
		SLE	Sierra Leone						
		TGO	Togo						
7 countries		***15 countries***		***11 countries***		***9 countries***		***12 countries***	

Table S3. *Statistical description of climate variables in African regions.*

Regions	NAf	WAf	CAf	EAf	SAf	NAf	WAf	CAf	EAf	SAf	NAf	WAf	CAf	EAf	SAf
			**pr (mm)**					**tdew (°C)**					**tmin (°C)**		
**Min**	16.3	423.1	636.5	1,127.3	778.5	-0.1	1.3	4.6	12.2	2.0	13.1	20.2	16.2	14.0	3.9
**Q1**	35.3	2,103.1	3,656.0	1,398.3	1,822.5	3.1	14.6	18.8	15.5	6.4	15.4	21.4	20.7	16.1	12.9
**Q2**	202.3	3,815.5	4,564.7	3,494.1	2,580.7	5.1	18.7	21.0	16.7	12.1	15.6	22.2	21.5	19.9	14.2
**Q3**	909.9	5,488.3	4,714.5	3,826.1	3,498.2	6.9	20.6	22.4	18.2	17.6	19.0	22.5	21.8	22.6	20.8
**Max**	1,865.5	10,044.6	6,599.3	6,329.1	4,767.3	8.5	22.5	23.4	23.7	22.5	20.0	23.1	26.1	27.1	26.4
			**tmoy (°C)**					**tmax (°C)**					**rh (%)**		
**Min**	19.3	23.7	21.7	18.3	9.5	27.2	24.4	26.9	24.0	16.2	22.7	22.3	28.1	51.0	37.2
**Q1**	22.0	25.4	24.6	21.5	18.9	29.5	30.8	28.1	27.8	26.2	28.6	53.3	73.6	59.1	55.6
**Q2**	23.2	26.6	25.0	25.6	20.6	31.4	32.8	28.7	28.7	27.2	38.7	67.9	81.3	63.2	64.6
**Q3**	26.1	28.0	25.2	27.6	24.8	34.0	34.9	29.8	33.6	29.1	44.3	76.9	84.7	76.7	72.5
**Max**	26.7	29.4	27.9	30.9	26.9	34.3	36.6	35.8	36.1	31.7	49.8	87.0	89.0	81.7	79.2
			**ah (%)**					**wspd (m/s)**					**insol (MJ/m²/day)**		
**Min**	4.9	6.4	7.9	11.4	6.2	2.0	1.5	0.1	0.6	1.8	20.5	16.7	14.9	16.8	17.3
**Q1**	6.4	14.0	16.6	13.5	8.4	3.1	1.8	0.3	1.7	2.4	21.2	18.1	16.2	19.2	19.5
**Q2**	7.3	16.7	18.4	14.9	11.6	3.2	2.2	1.0	2.1	2.7	22.0	19.2	16.9	20.6	20.3
**Q3**	8.6	18.4	19.9	16.0	15.3	3.5	2.7	1.7	3.3	3.3	22.7	20.9	19.5	21.6	21.0
**Max**	10.1	20.1	20.8	21.2	19.7	3.8	5.6	3.6	4.6	5.9	23.6	23.3	23.9	22.7	22.4

Regions: Northern Africa (NAf), Western Africa (WAf), Central Africa (CAf), Eastern Africa (EAf) and Southern Africa (SAf). Min: minimum value for countries in the region. Max: maximum value for countries in the region. Q1, Q2, Q3: First, second and third quartiles.

Table S4. Complete list of significant (Wald test – p-value < 5%) *RegARIMA* models fitted to 54 African countries cumulative cases.

**N°**	**Country**	**Climate variable**	**Lag (days)**	**RegARIMA model (p,d,q)**	**NRMSE**	**NMAE**	**Coefficient of climate variable**	**Wald test (p-value)**	**Spearman's ρ**	**Spearman test (p-value)**
1	AGO	insol	11	(1,1,1)	0.0019	0.0005	-0.0008	0.0163	-0.133	<0.0001
2	BDI	pr	3	(1,1,2)	0.0043	0.0014	0.0061	0.0183	0.073	0.0275
3	BDI	tmax	15	(1,1,1)	0.0044	0.0015	-0.0024	0.0076	-0.139	<0.0001
4	BEN	pr	23	(2,1,2)	0.0056	0.0020	-0.0078	0.0005	-0.083	0.0130
5	CIV	ah	15	(2,1,4)	0.0012	0.0006	-0.0016	0.0040	-0.166	<0.0001
6	CIV	tdew	15	(2,1,4)	0.0013	0.0006	-0.0015	0.0133	-0.168	<0.0001
7	CIV	tmoy	9	(2,1,4)	0.0012	0.0006	-0.0010	0.0104	-0.071	0.0304
8	CIV	wspd	17	(2,1,4)	0.0013	0.0006	0.0005	0.0210	0.136	<0.0001
9	COD	pr	17	(2,1,4)	0.0025	0.0010	0.0018	0.0221	0.091	0.0059
10	COG	ah	8	(3,1,2)	0.0034	0.0017	-0.0033	0.0012	-0.189	<0.0001
11	COG	tdew	8	(3,1,2)	0.0034	0.0017	-0.0032	0.0022	-0.188	<0.0001
12	COM	ah	13	(4,1,2)	0.0023	0.0009	-0.0021	0.0232	-0.080	0.0185
13	COM	tdew	13	(4,1,2)	0.0023	0.0009	-0.0021	0.0272	-0.069	0.0422
14	DZA	tdew	1	(3,2,3)	0.0002	0.0001	-0.0001	0.0486	-0.087	0.0071
15	EGY	wspd	4	(5,1,3)	0.0007	0.0002	0.0004	0.0009	0.073	0.0243
16	ERI	insol	28	(2,1,2)	0.0019	0.0009	-0.0009	0.0450	-0.068	0.0404
17	ETH	tmoy	18	(1,1,2)	0.0005	0.0002	-0.0004	0.0138	-0.070	0.0350
18	GAB	rh	13	(2,1,3)	0.0023	0.0011	0.0013	0.0399	0.340	<0.0001
19	GAB	tmax	4	(2,1,3)	0.0023	0.0011	-0.0013	0.0035	-0.319	<0.0001

N°	Country	Climate variable	Lag (days)	RegARIMA model (p,d,q)	NRMSE	NMAE	Coefficient of climate variable	Wald test (p-value)	Spearman's ρ	Spearman test (p-value)
20	GAB	tmoy	4	(2,1,3)	0.0023	0.0011	-0.0017	0.0057	-0.384	<0.0001
21	GHA	rh	7	(1,1,4)	0.0015	0.0009	-0.0012	0.0393	-0.209	<0.0001
22	GIN	ah	0	(2,1,1)	0.0013	0.0008	-0.0016	0.0039	-0.083	0.0112
23	GIN	tdew	0	(2,1,1)	0.0013	0.0008	-0.0014	0.0121	-0.083	0.0117
24	GNB	rh	6	(1,1,2)	0.0023	0.0011	0.0023	0.0374	0.087	0.0085
25	GNB	tmax	0	(1,1,2)	0.0023	0.0011	-0.0026	0.0051	-0.132	<0.0001
26	GNB	tmoy	9	(1,1,2)	0.0023	0.0011	-0.0034	0.0089	-0.107	0.0012
27	KEN	pr	26	(4,1,2)	0.0006	0.0003	-0.0015	<0.0001	-0.157	<0.0001
28	KEN	wspd	7	(4,1,2)	0.0006	0.0003	0.0006	0.0176	0.129	<0.0001
29	LBR	ah	21	(2,1,2)	0.0038	0.0015	-0.0051	0.0079	-0.101	0.0023
30	LBR	insol	1	(2,1,2)	0.0038	0.0013	0.0017	0.0248	0.075	0.0224
31	LBR	tdew	21	(2,1,2)	0.0038	0.0015	-0.0051	0.0114	-0.098	0.0031
32	MDG	insol	28	(3,1,5)	0.0026	0.0011	-0.0015	0.0110	-0.066	0.0491
33	MDG	tmoy	12	(3,1,5)	0.0025	0.0011	-0.0031	0.0087	-0.088	0.0081
34	MOZ	rh	0	(1,1,0)	0.0011	0.0004	-0.0005	0.0313	-0.184	<0.0001
35	MOZ	tdew	0	(1,1,0)	0.0011	0.0004	-0.0006	0.0313	-0.088	0.0077
36	MRT	insol	25	(1,1,0)	0.0006	0.0003	-0.0003	0.0078	-0.234	<0.0001
37	MWI	rh	26	(2,1,4)	0.0009	0.0004	0.0006	0.0065	0.148	<0.0001
38	RWA	tmin	0	(3,1,3)	0.0010	0.0003	-0.0006	0.0110	-0.080	0.0150
39	RWA	tmoy	4	(3,1,4)	0.0010	0.0003	-0.0009	0.0029	-0.103	0.0017
40	SEN	ah	3	(2,1,2)	0.0007	0.0003	0.0006	0.0428	0.090	0.0060

N°	Country	Climate variable	Lag (days)	RegARIMA model (p,d,q)	NRMSE	NMAE	Coefficient of climate variable	Wald test (p-value)	Spearman's ρ	Spearman test (p-value)
41	SEN	insol	26	(2,1,2)	0.0007	0.0004	-0.0003	0.0058	-0.229	<0.0001
42	SLE	insol	25	(1,1,2)	0.0014	0.0006	0.0005	0.0301	0.108	0.0012
43	SOM	rh	19	(2,1,2)	0.0028	0.0013	-0.0027	0.0096	-0.201	<0.0001
44	SOM	tmoy	5	(2,1,2)	0.0028	0.0013	-0.0023	0.0293	-0.079	0.0161
45	SSD	ah	4	(1,1,1)	0.0023	0.0010	-0.0028	0.0078	-0.132	<0.0001
46	SSD	tdew	4	(1,1,1)	0.0023	0.0010	-0.0024	0.0290	-0.126	0.0002
47	SSD	tmin	17	(3,1,1)	0.0023	0.0010	-0.0019	0.0146	-0.073	0.0296
48	STP	tmin	15	(1,1,2)	0.0027	0.0011	-0.0047	<0.0001	-0.071	0.0332
49	SWZ	rh	23	(2,1,5)	0.0011	0.0004	0.0004	0.0377	0.166	<0.0001
50	SYC	tdew	23	(3,1,3)	0.0026	0.0012	-0.0015	0.0497	-0.283	<0.0001
51	SYC	tmax	3	(5,1,4)	0.0025	0.0011	-0.0028	0.0163	-0.247	<0.0001
52	SYC	tmin	16	(3,1,3)	0.0026	0.0012	-0.0030	0.0116	-0.245	<0.0001
53	SYC	tmoy	17	(3,1,3)	0.0026	0.0012	-0.0027	0.0347	-0.245	<0.0001
54	TGO	ah	5	(1,1,2)	0.0015	0.0006	-0.0011	0.0206	-0.271	<0.0001
55	TGO	rh	8	(1,1,2)	0.0015	0.0006	-0.0010	0.0299	-0.164	<0.0001
56	TGO	tdew	5	(1,1,2)	0.0015	0.0006	-0.0012	0.0220	-0.272	<0.0001
57	TUN	rh	9	(2,1,2)	0.0016	0.0007	-0.0008	0.0275	-0.086	0.0088
58	ZAF	ah	5	(2,1,5)	0.0005	0.0002	0.0003	0.0111	0.093	0.0043
59	ZAF	insol	7	(1,1,3)	0.0005	0.0002	-0.0003	0.0028	-0.086	0.0086

NRMSE: Normalized root mean square error – NMAE: normalized mean absolute error.

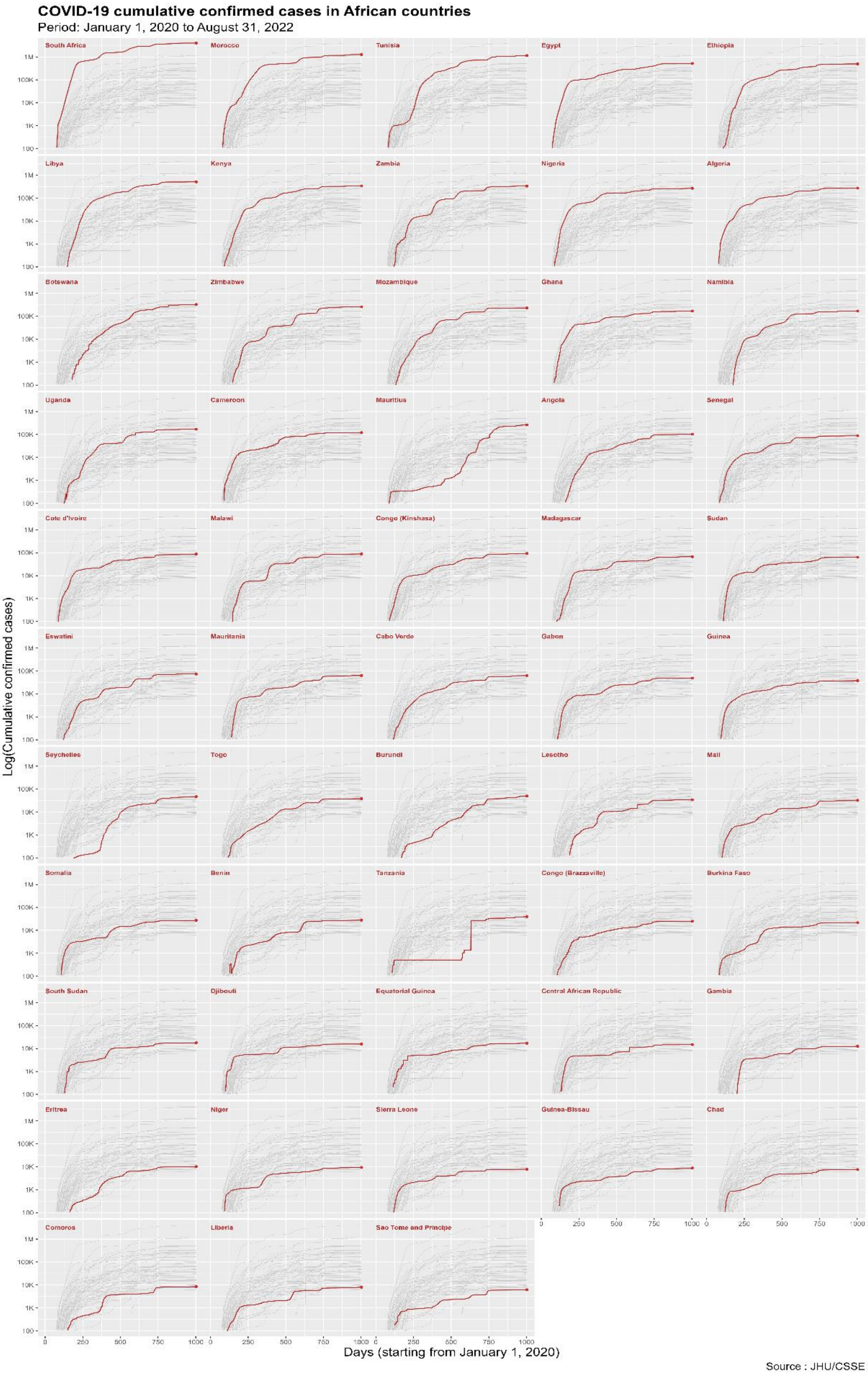

Figure S1. Individual COVID-19 cumulative cases in African countries. Counts of cumulative cases is expressed on log scale (vertical axis). The countries are sorted in decreasing order of incidence. This figure is adapted from Kieran Healy's *COVID-19 small multiple* (see https://kieranhealy.org/blog/archives/2020/03/27/a-covid-small-multiple/)

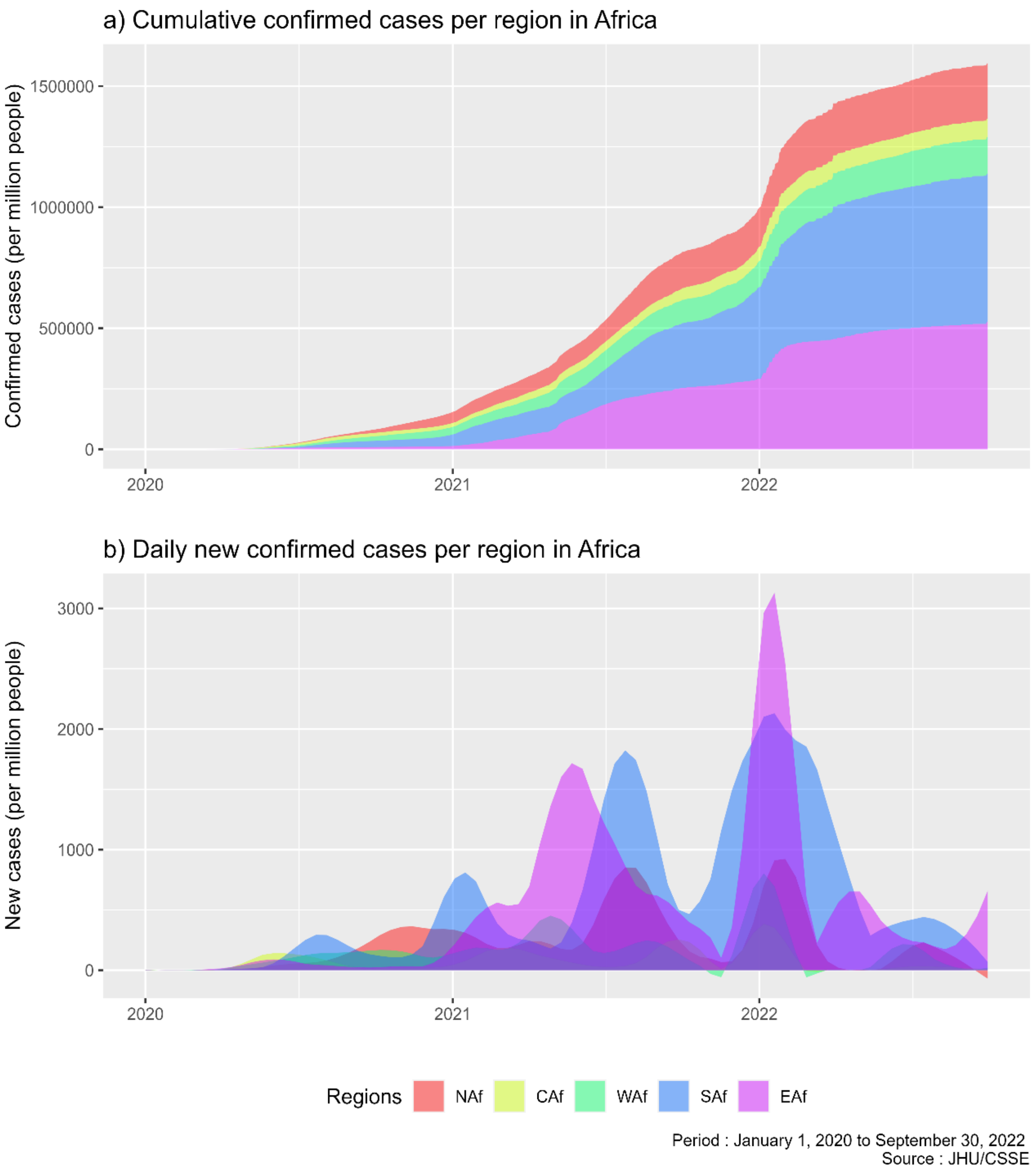

Figure S2. Evolution of the COVID-19 confirmed cases in Africa. (a) Cumulative confirmed cases per million people. (b) New daily confirmed cases per million people.

Plots of Autocorrelation function (ACF) for all countries and climate variables. Plots are sorted by countries names alphabetic order.

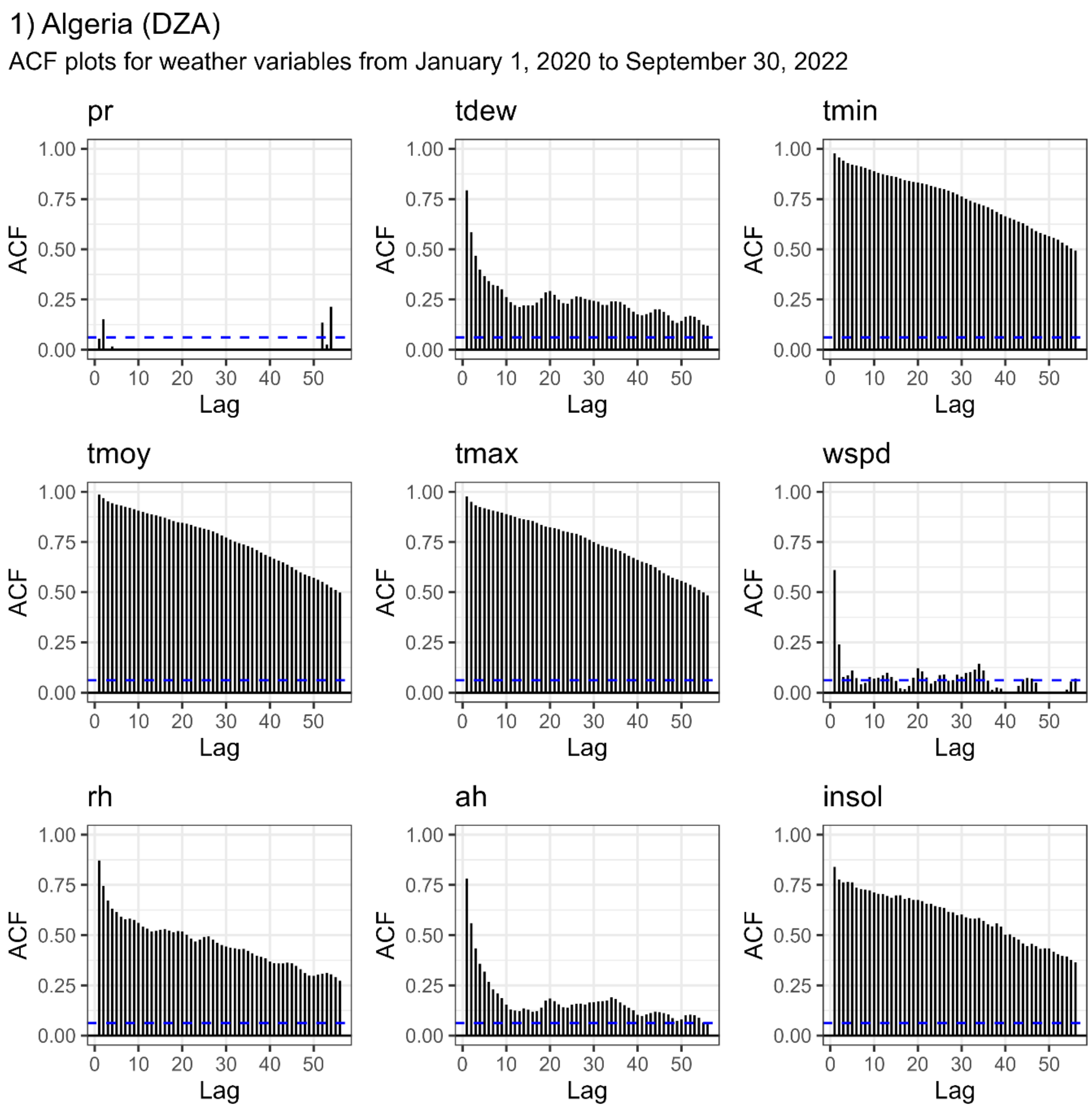

2) Angola (AGO)

ACF plots for weather variables from January 1, 2020 to September 30, 2022

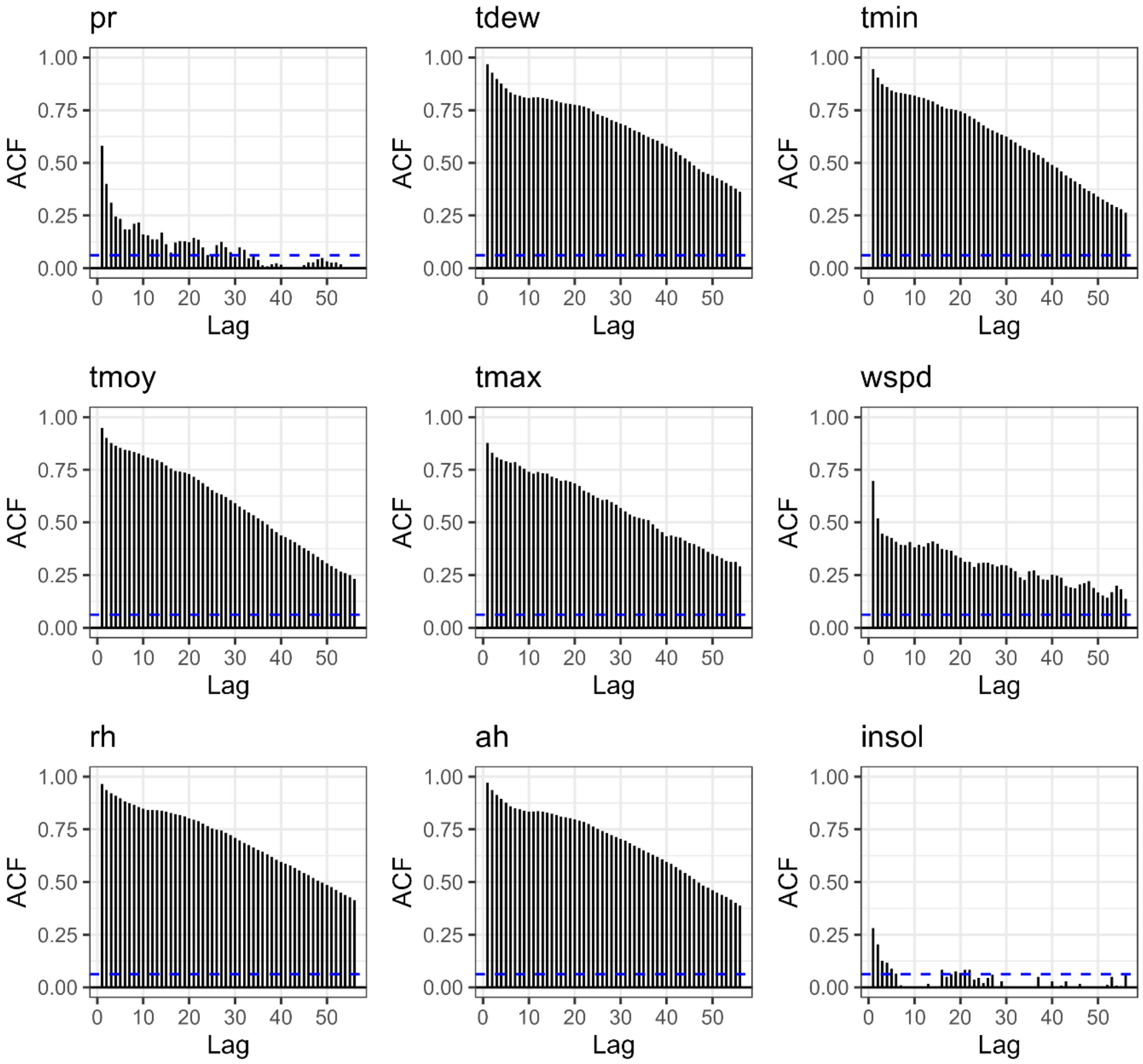

3) Benin (BEN)

ACF plots for weather variables from January 1, 2020 to September 30, 2022

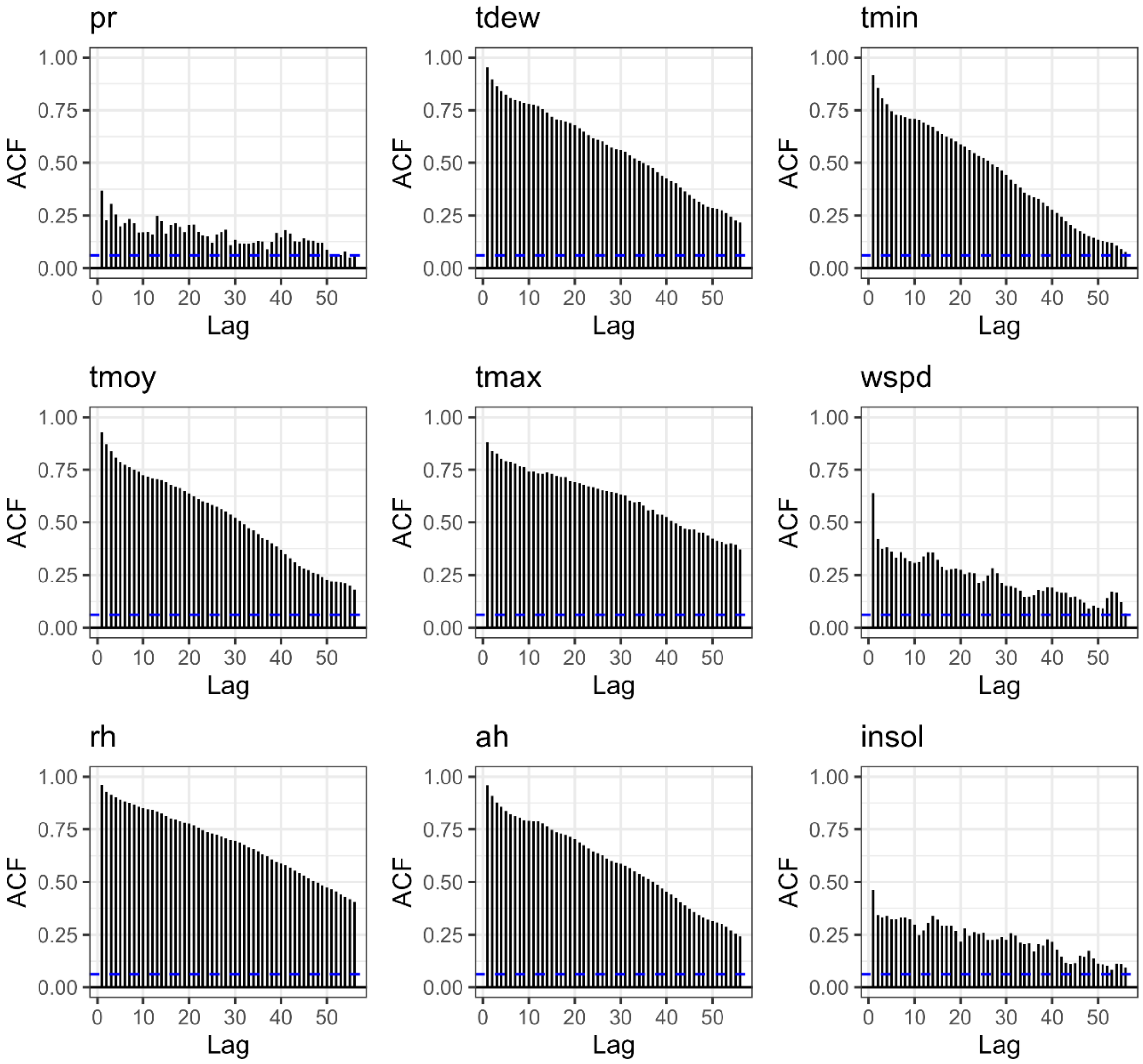

4) Botswana (BWA)

ACF plots for weather variables from January 1, 2020 to September 30, 2022

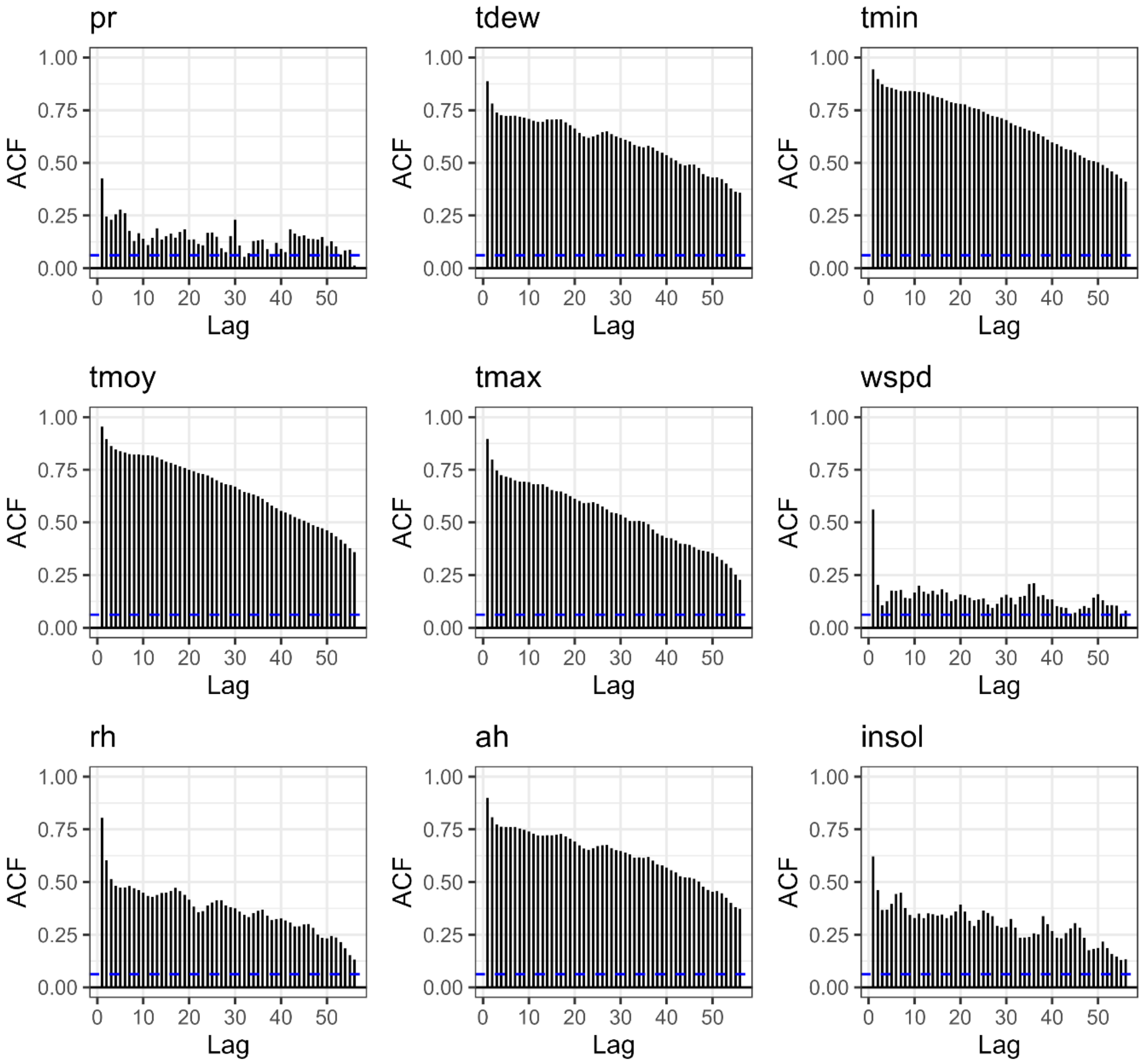

5) Burkina Faso (BFA)

ACF plots for weather variables from January 1, 2020 to September 30, 2022

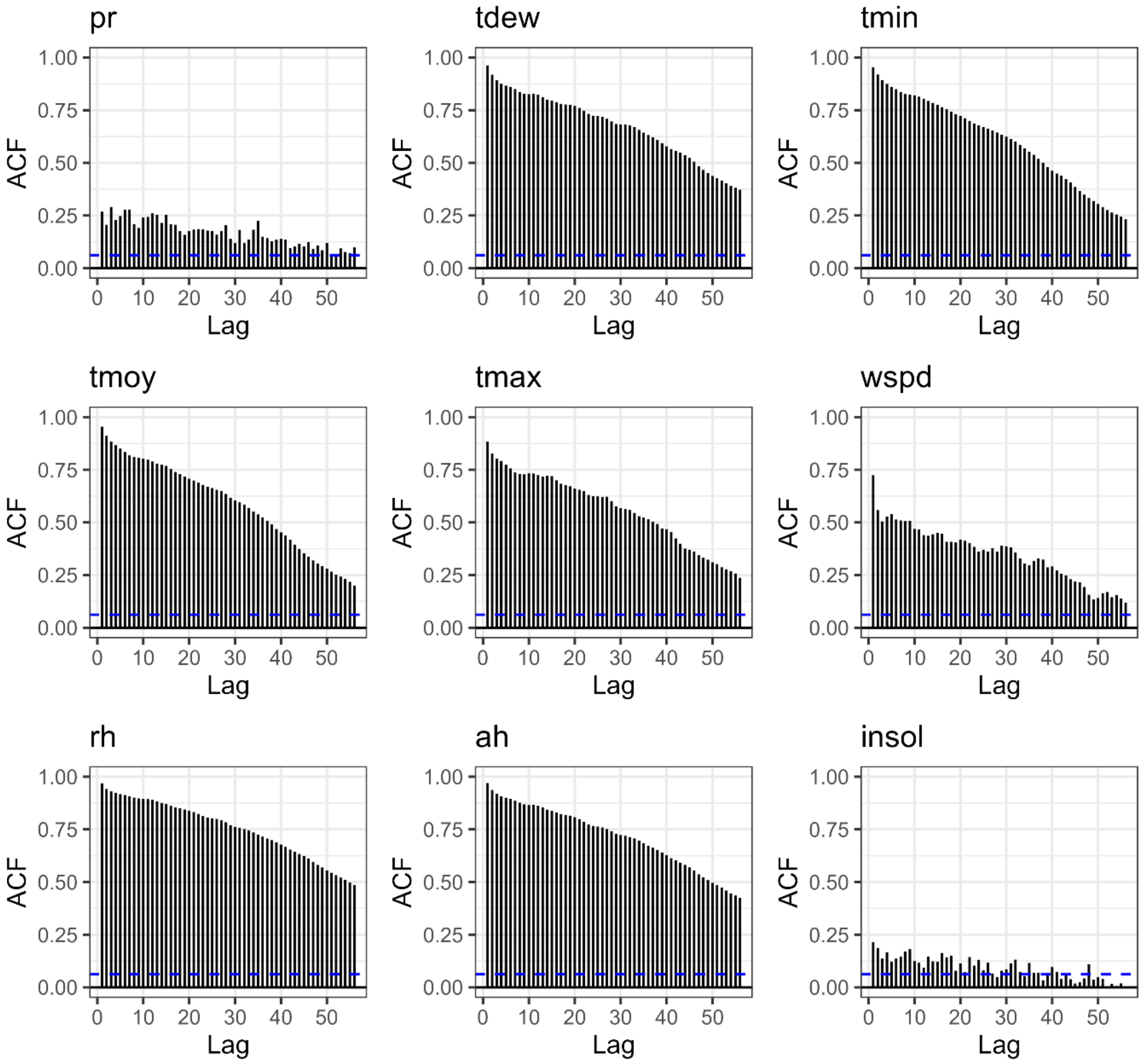

6) Burundi (BDI)

ACF plots for weather variables from January 1, 2020 to September 30, 2022

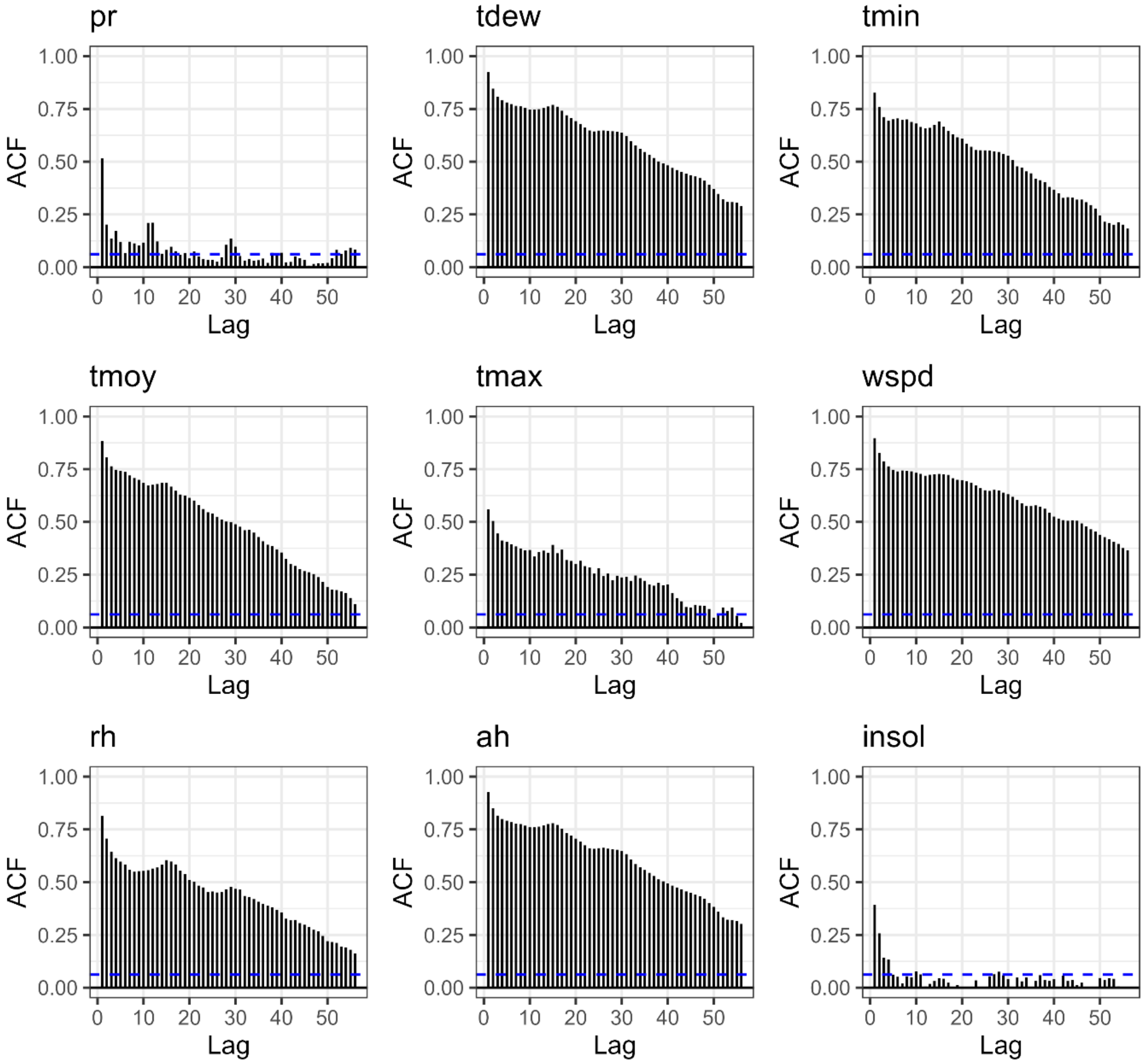

7) Cabo Verde (CPV)

ACF plots for weather variables from January 1, 2020 to September 30, 2022

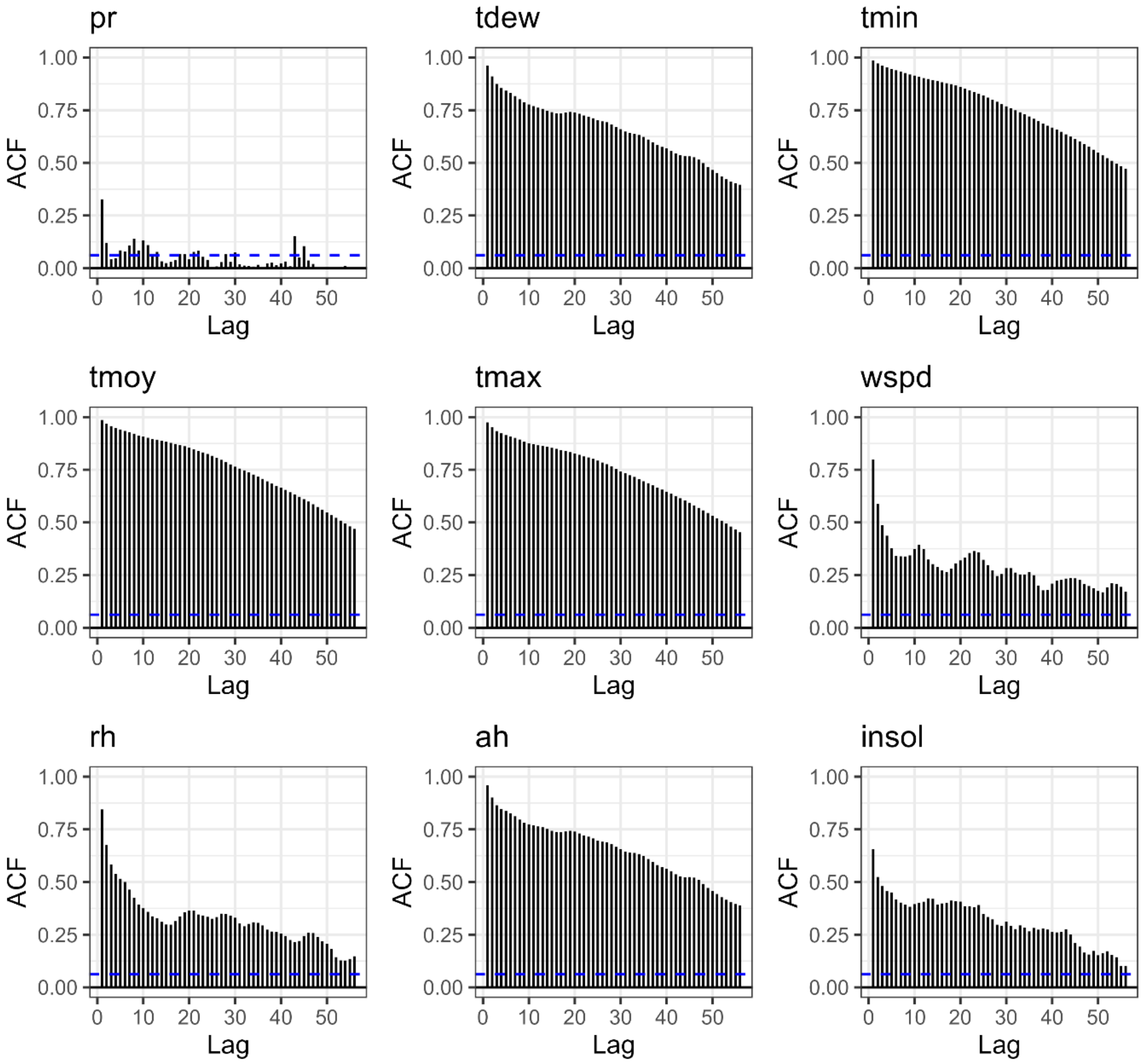

8) Cameroon (CMR)

ACF plots for weather variables from January 1, 2020 to September 30, 2022

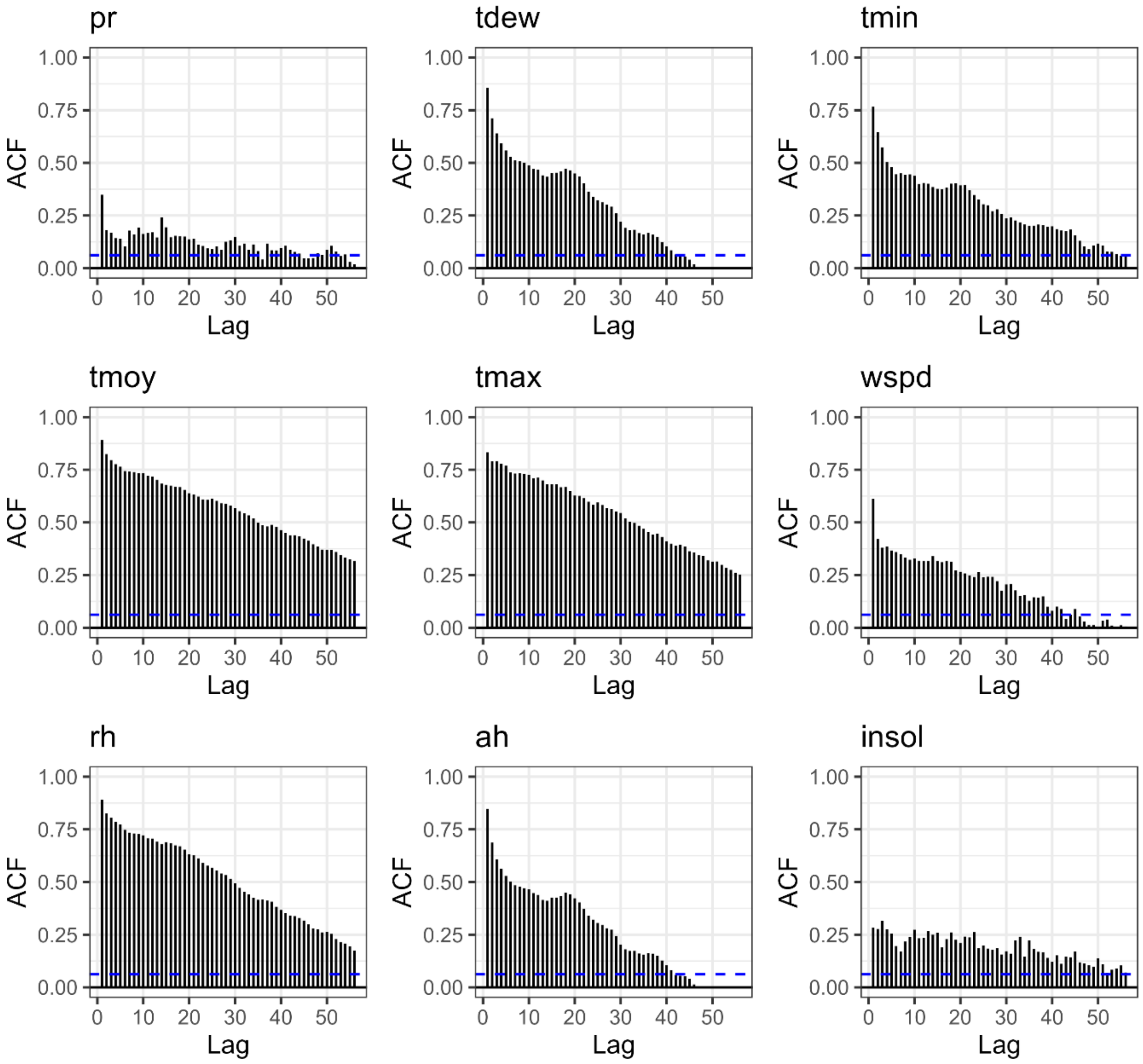

9) Central African Republic (CAF)

ACF plots for weather variables from January 1, 2020 to September 30, 2022

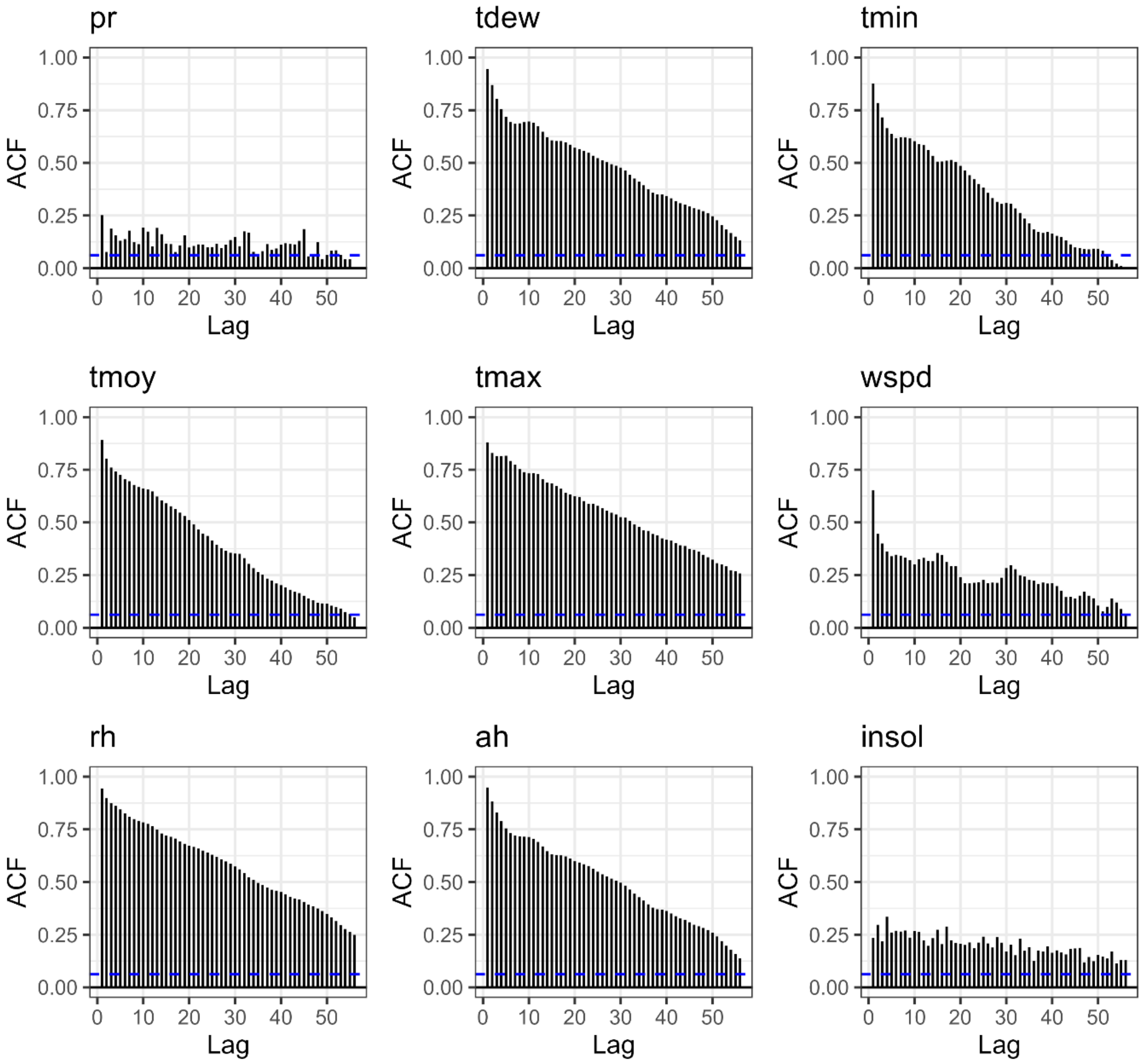

10) Chad (TCD)

ACF plots for weather variables from January 1, 2020 to September 30, 2022

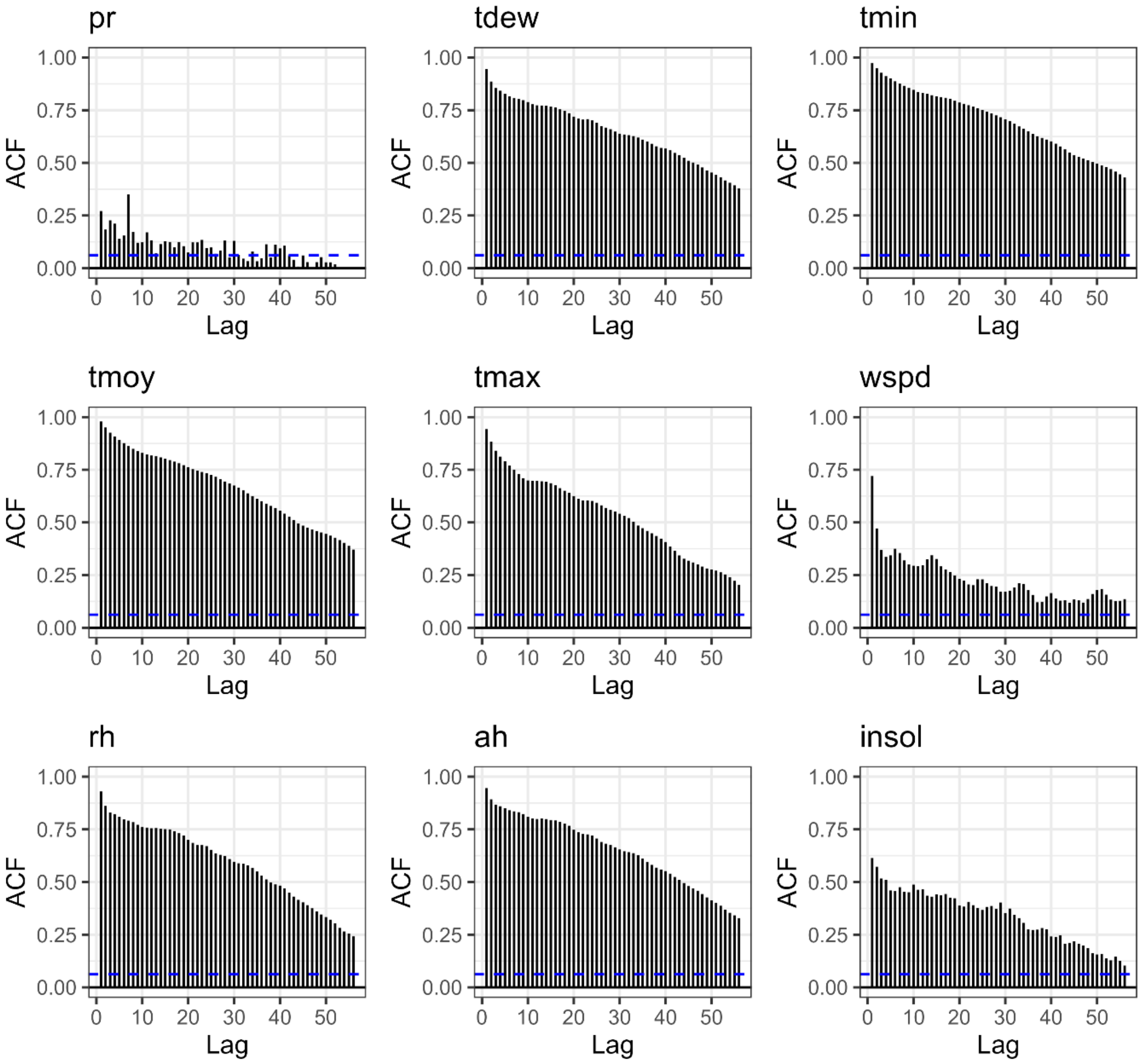

11) Comoros (COM)

ACF plots for weather variables from January 1, 2020 to September 30, 2022

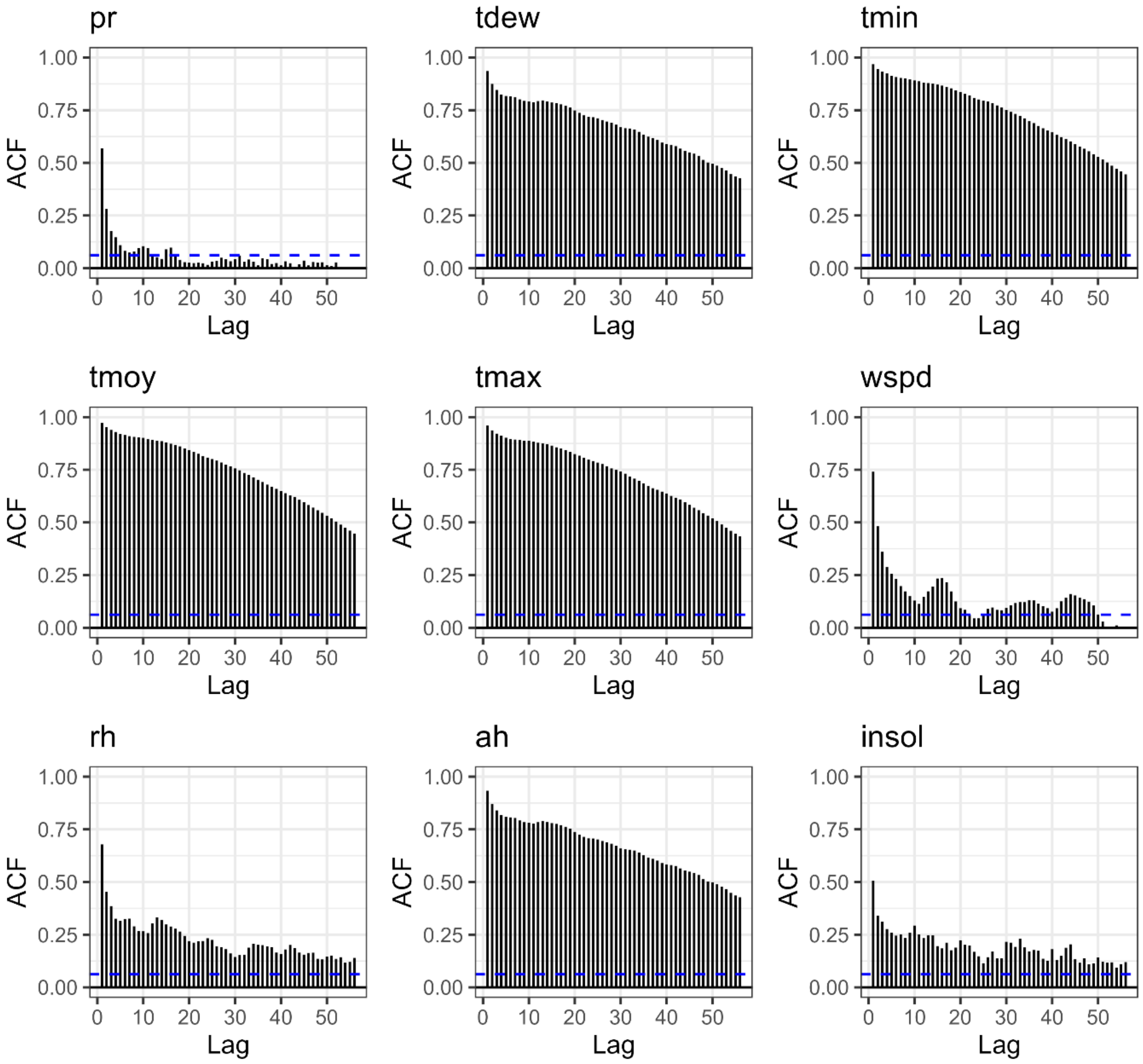

12) Congo (Brazzaville) (COG)

ACF plots for weather variables from January 1, 2020 to September 30, 2022

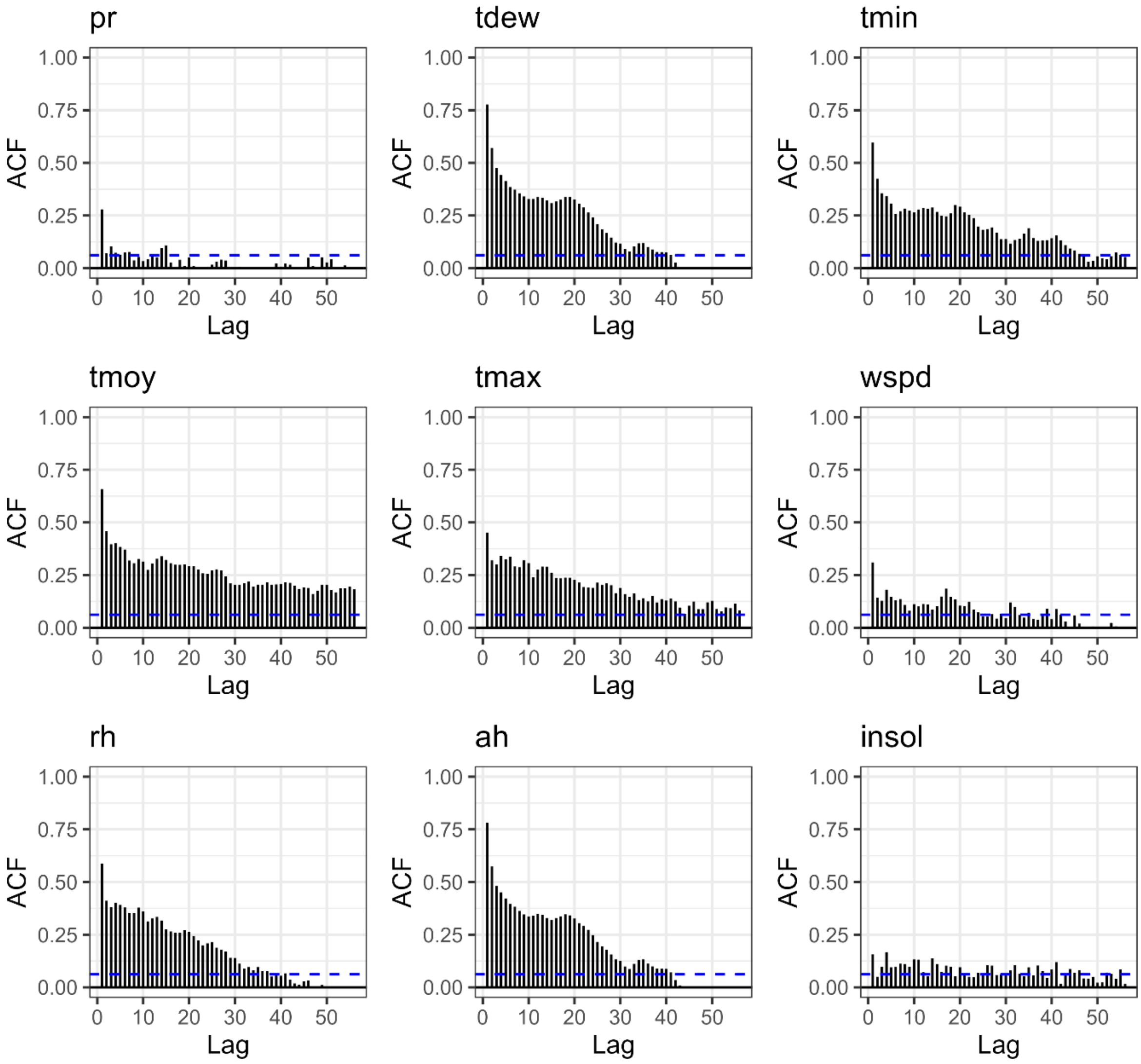

13) Congo (Kinshasa) (COD)

ACF plots for weather variables from January 1, 2020 to September 30, 2022

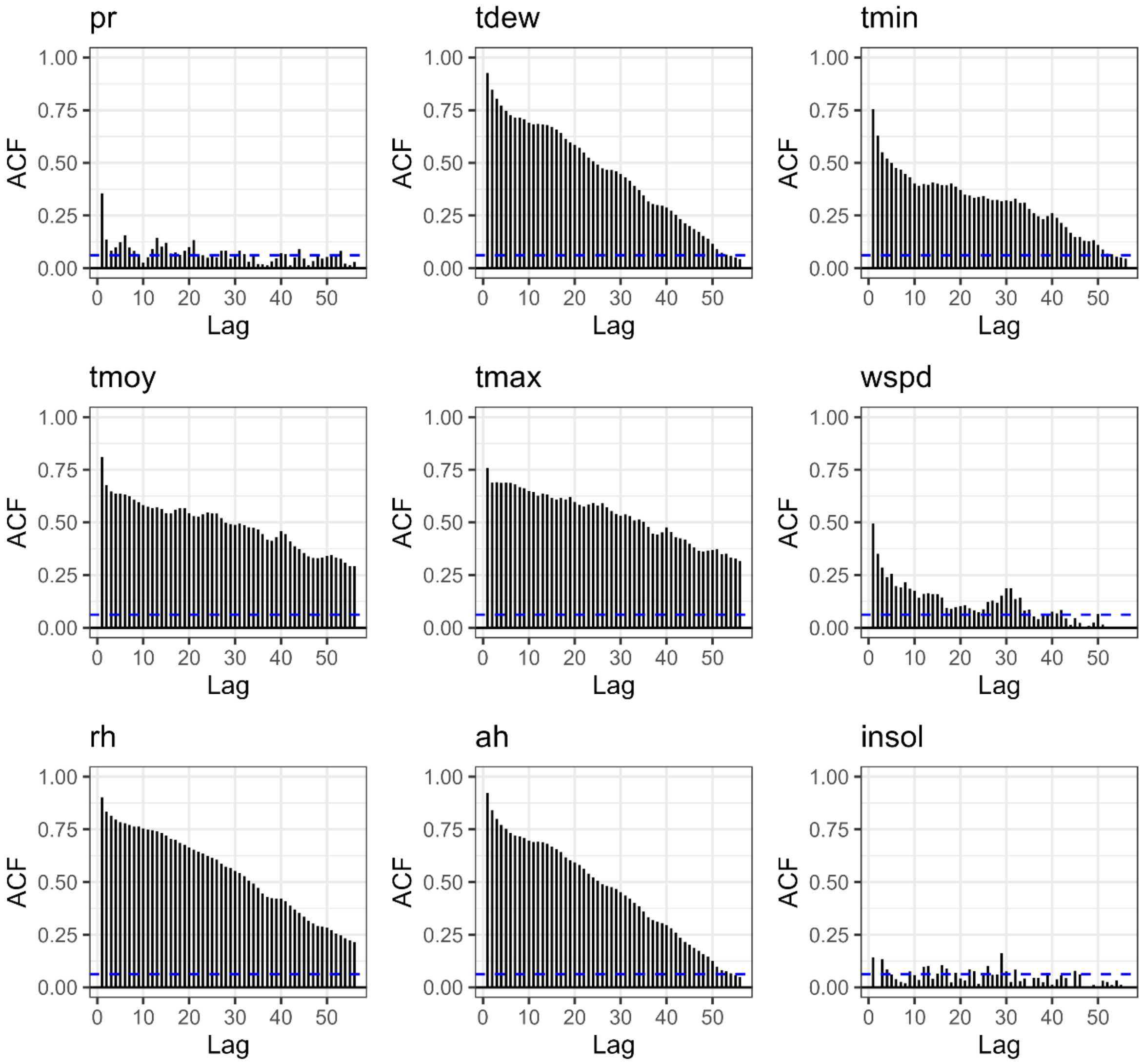

14) Cote d'Ivoire (CIV)

ACF plots for weather variables from January 1, 2020 to September 30, 2022

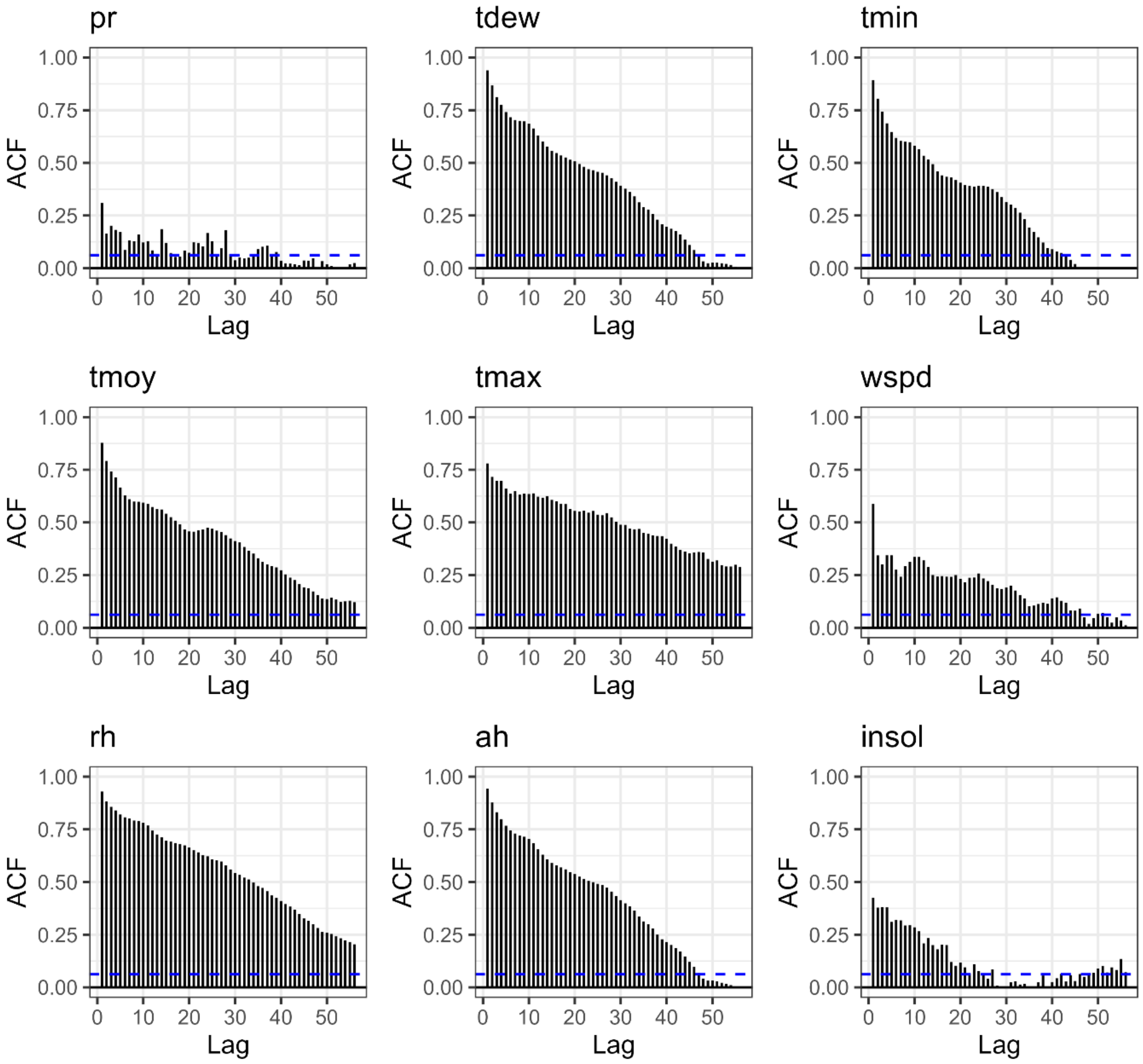

15) Djibouti (DJI)

ACF plots for weather variables from January 1, 2020 to September 30, 2022

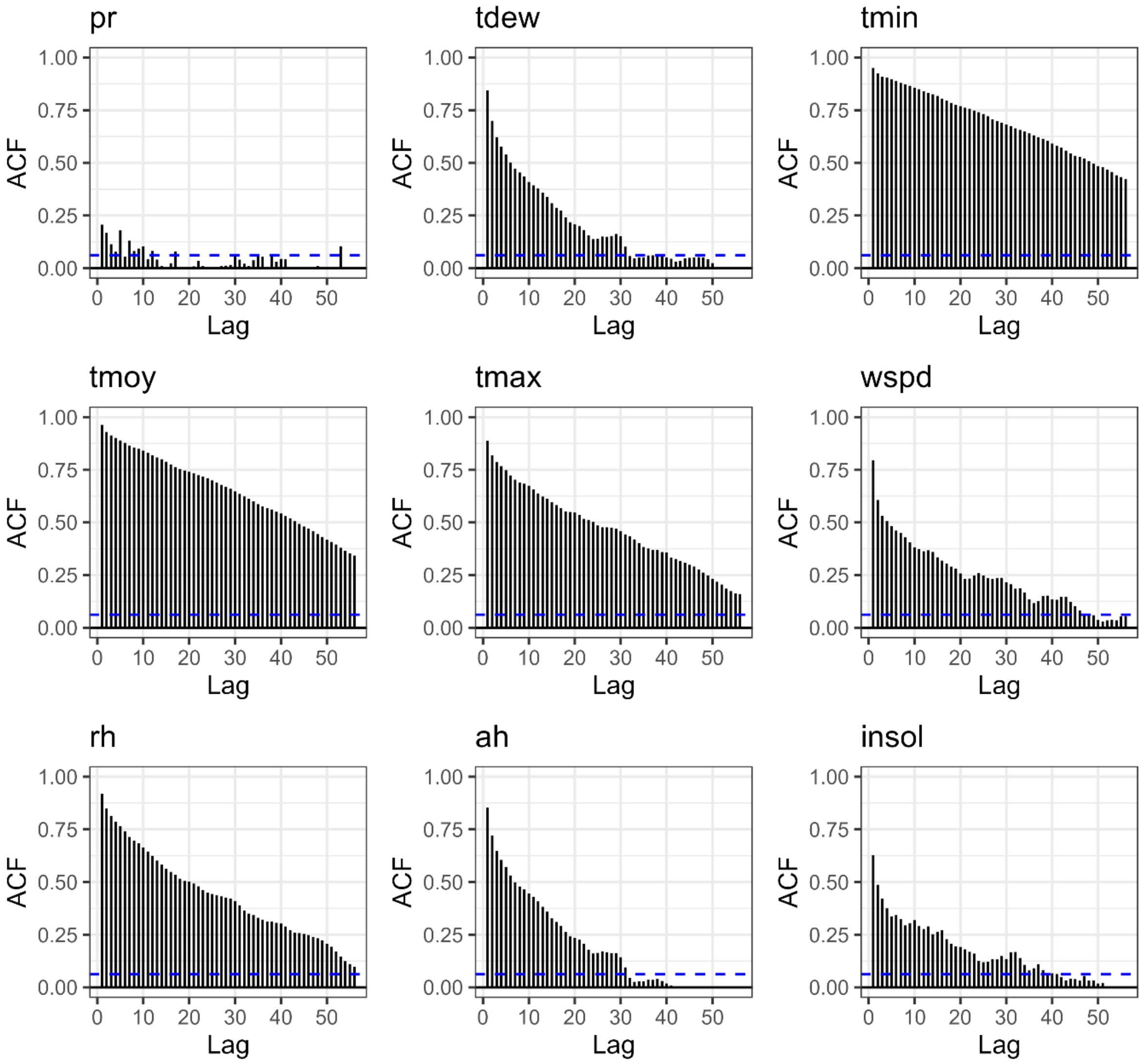

16) Egypt (EGY)

ACF plots for weather variables from January 1, 2020 to September 30, 2022

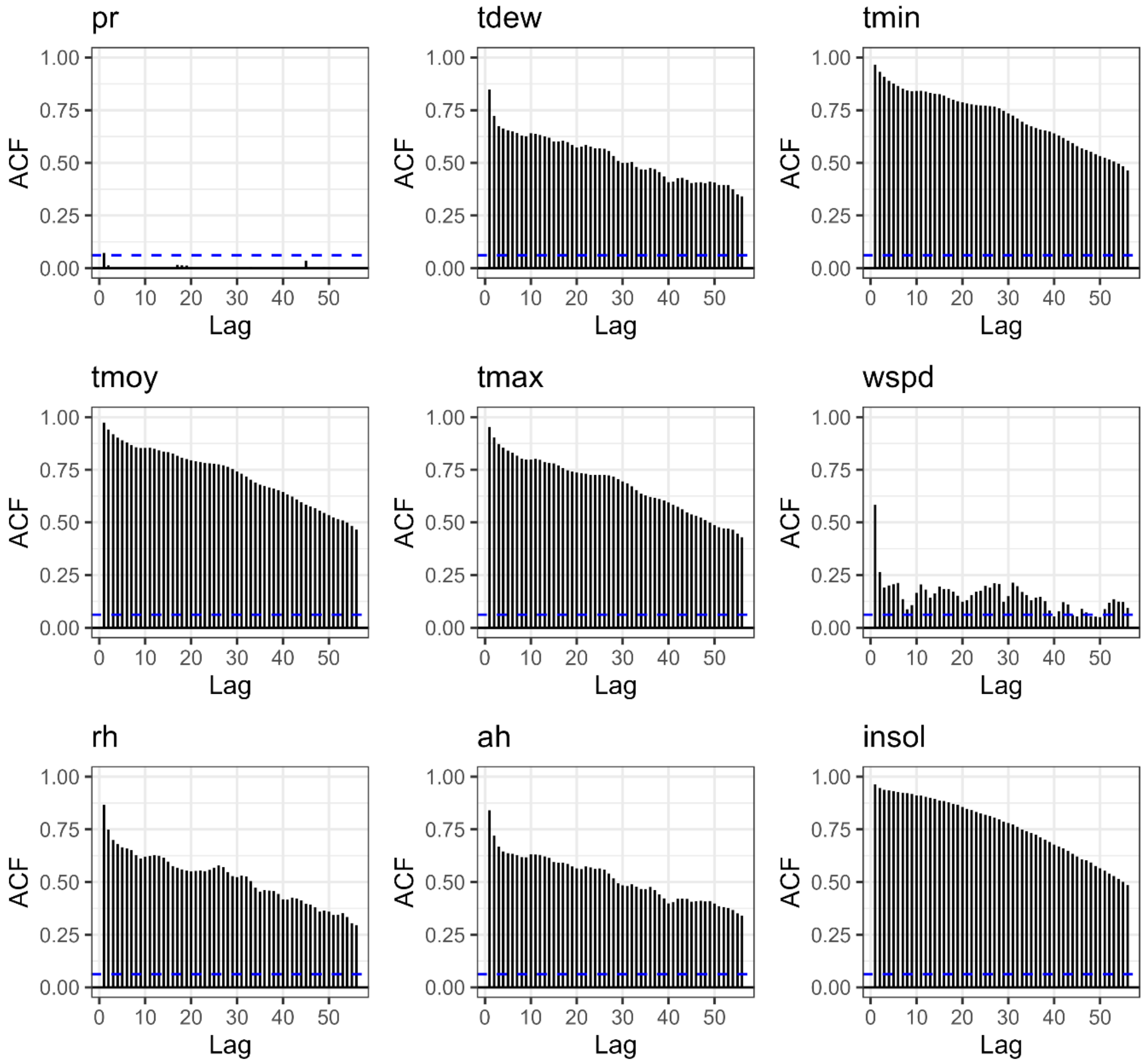

17) Equatorial Guinea (GNQ)

ACF plots for weather variables from January 1, 2020 to September 30, 2022

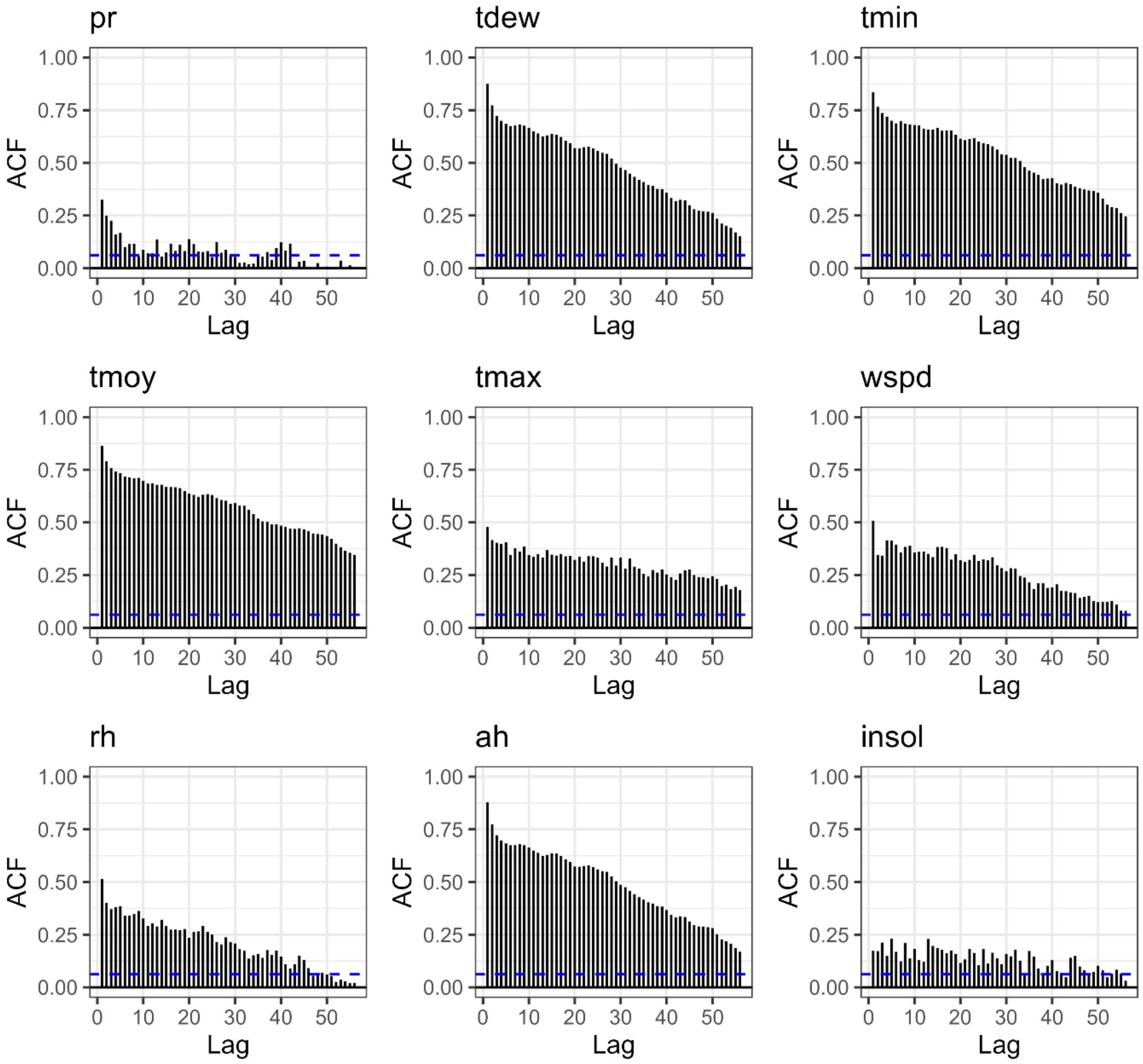

18) Eritrea (ERI)

ACF plots for weather variables from January 1, 2020 to September 30, 2022

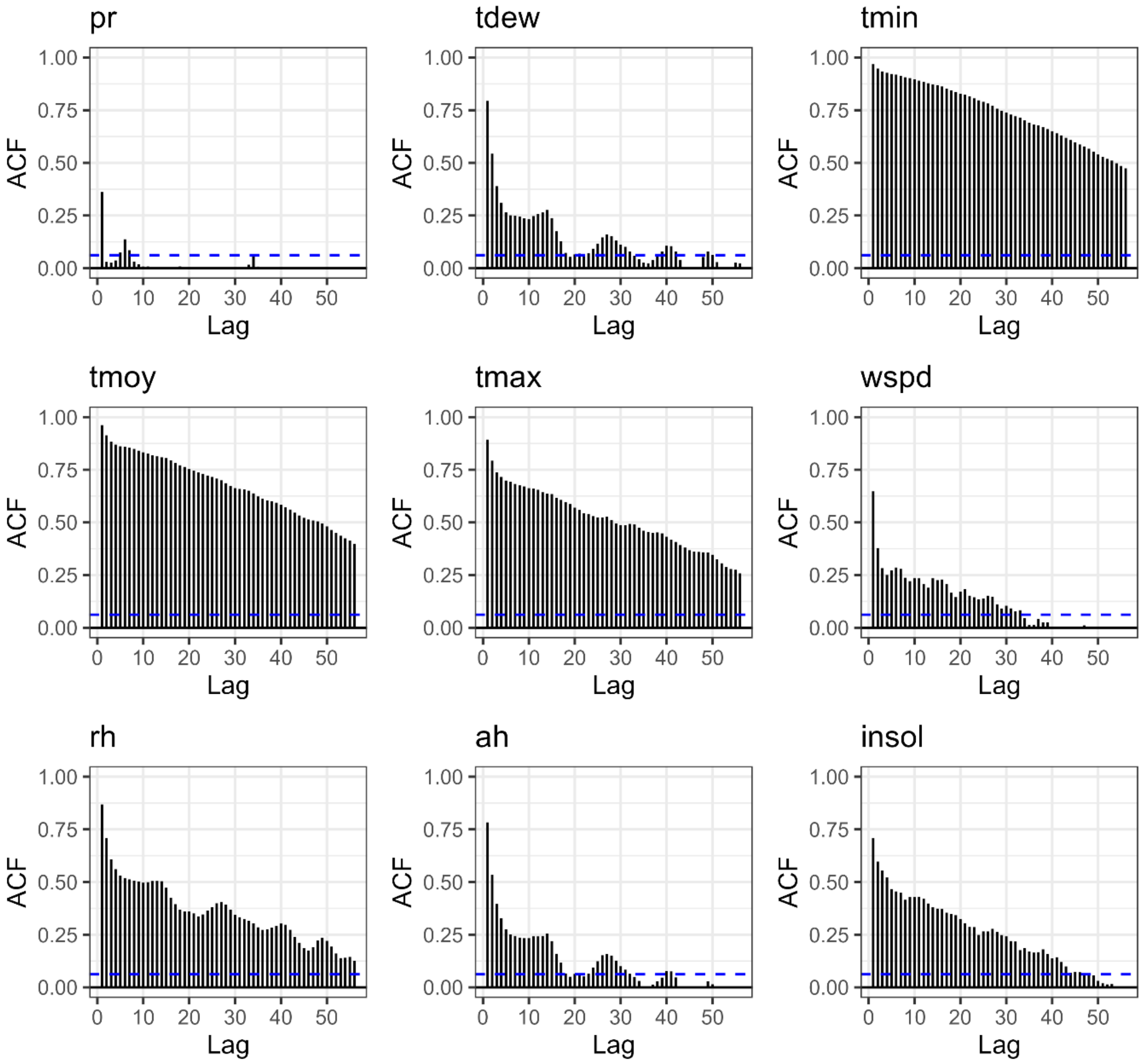

19) Eswatini (SWZ)

ACF plots for weather variables from January 1, 2020 to September 30, 2022

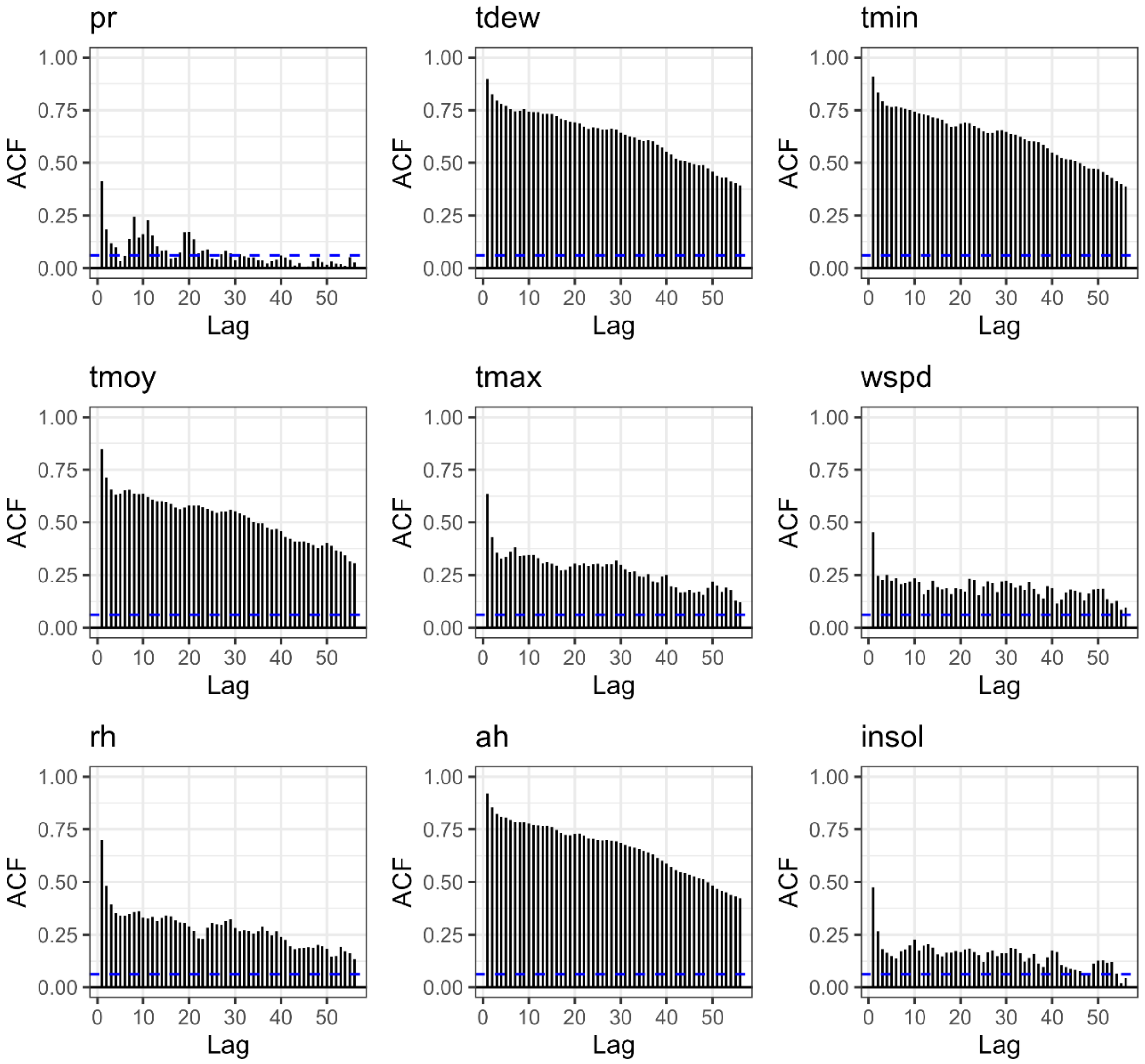

20) Ethiopia (ETH)

ACF plots for weather variables from January 1, 2020 to September 30, 2022

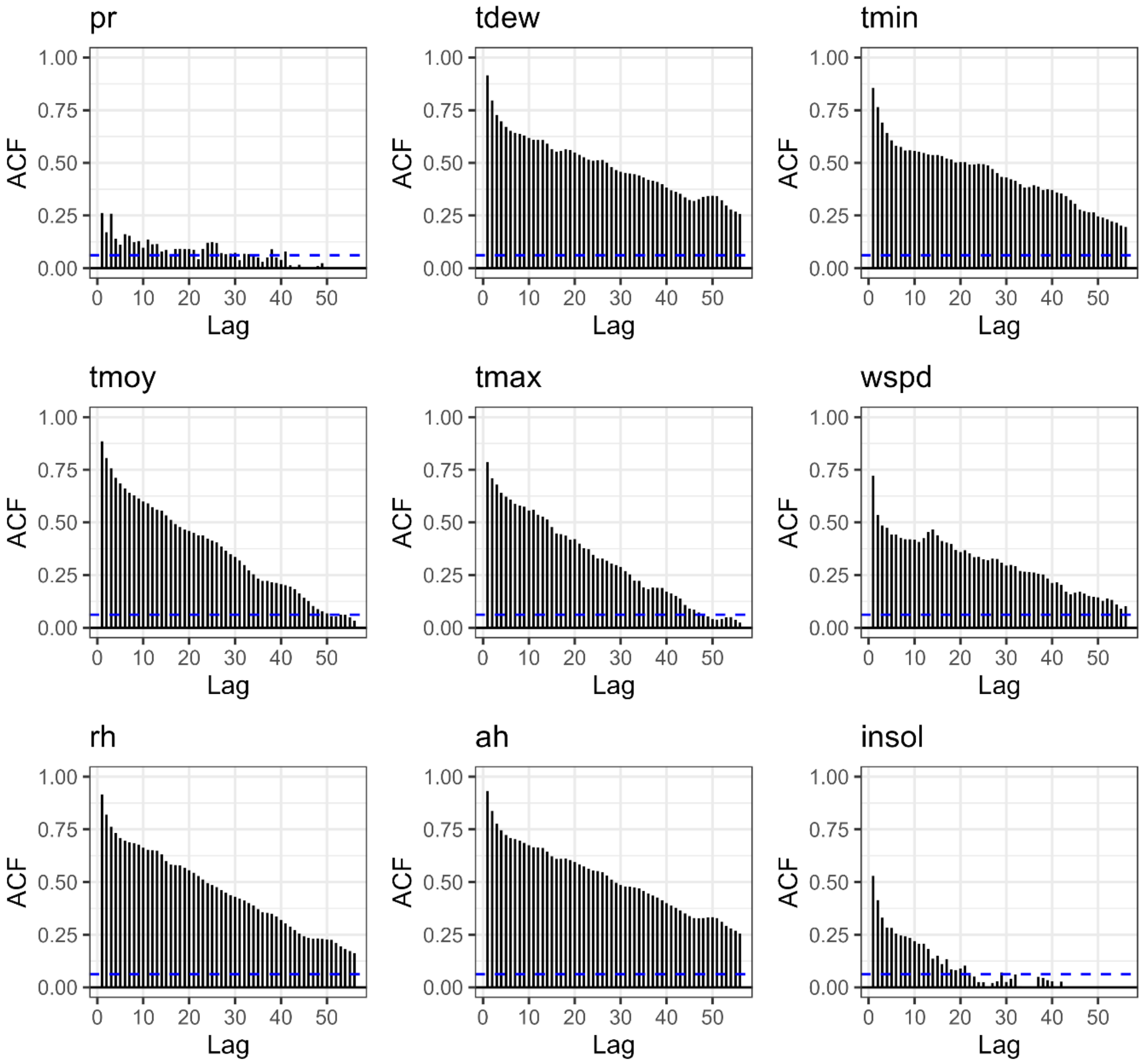

21) Gabon (GAB)

ACF plots for weather variables from January 1, 2020 to September 30, 2022

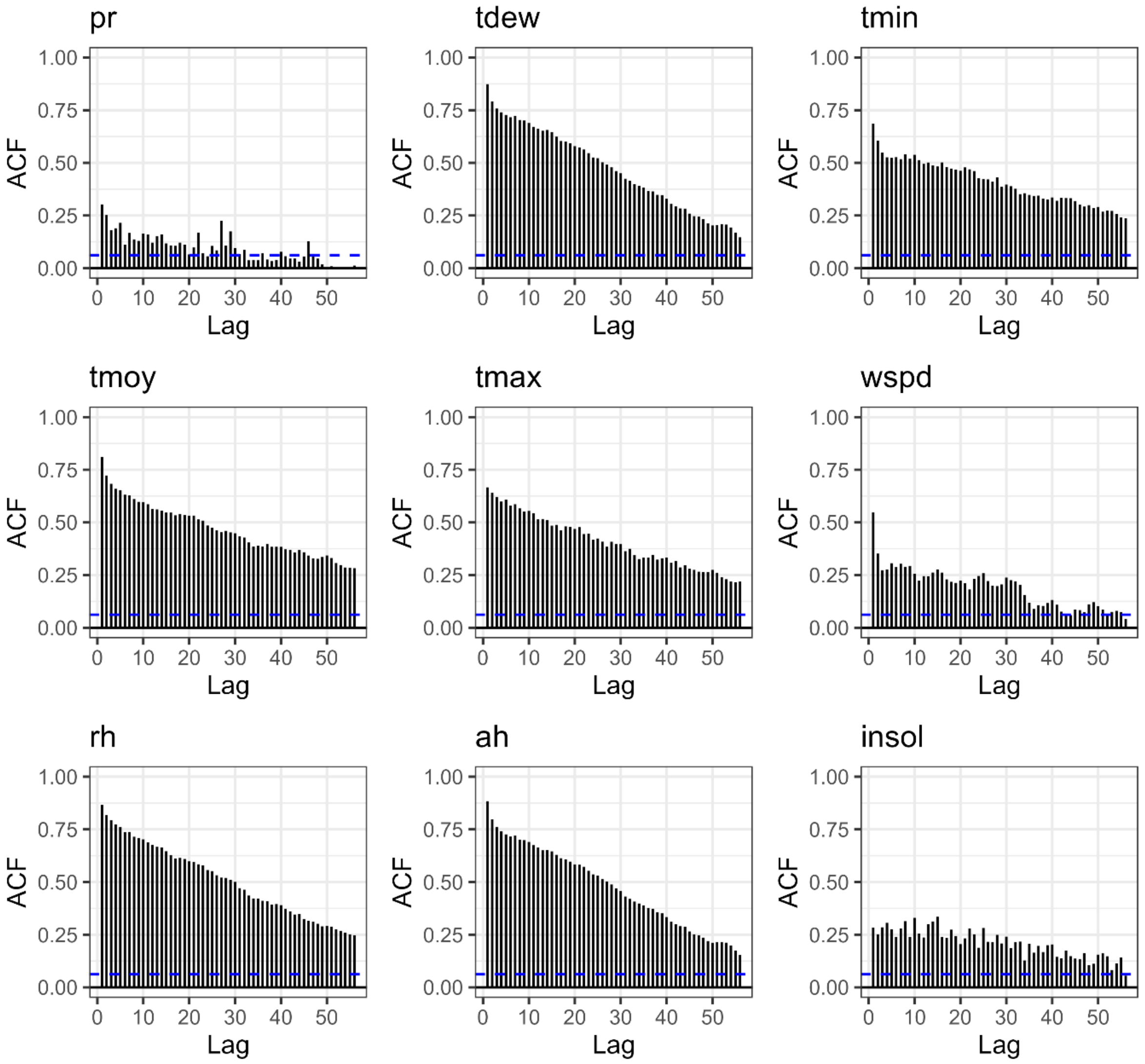

22) Gambia (GMB)

ACF plots for weather variables from January 1, 2020 to September 30, 2022

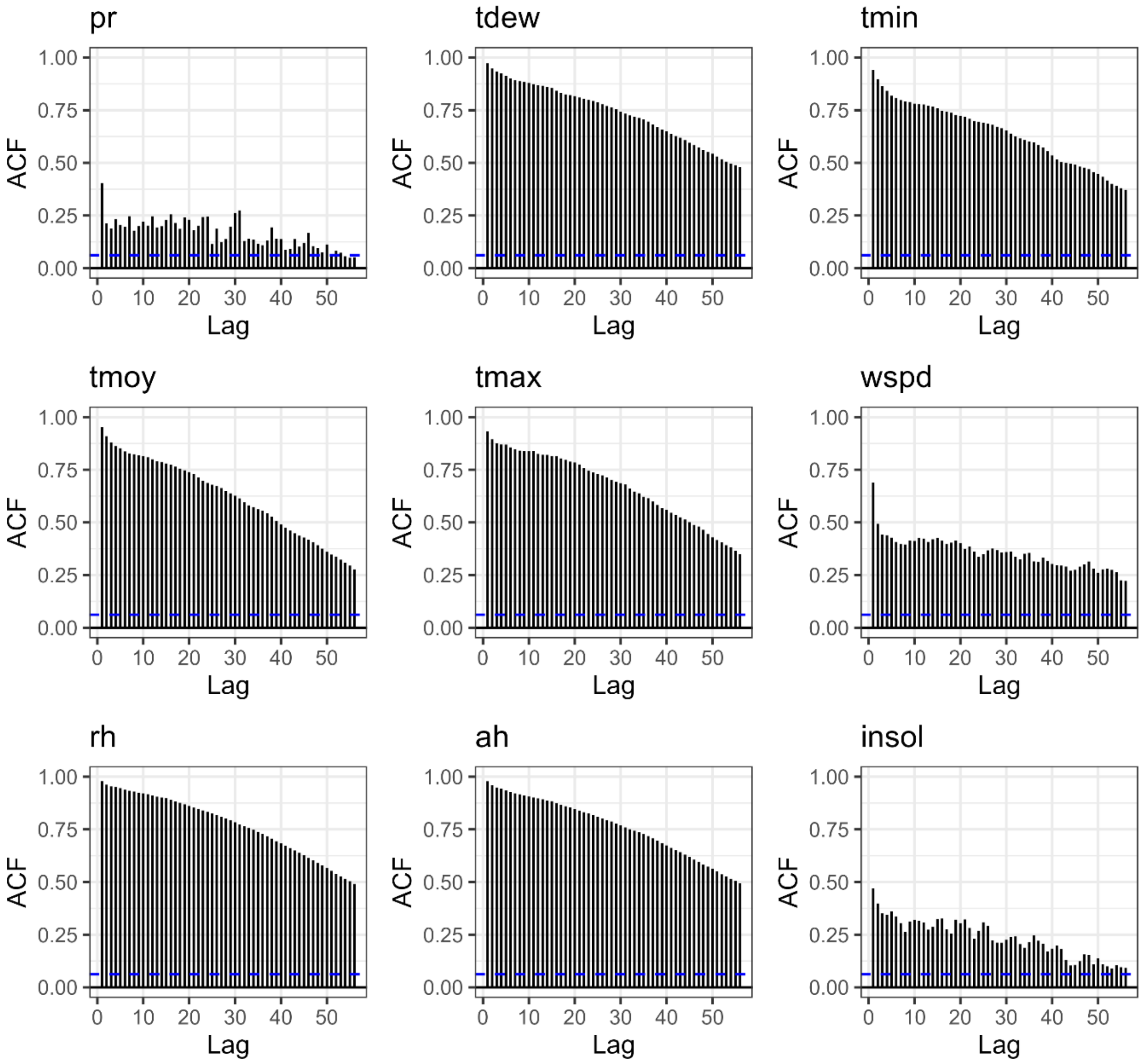

23) Ghana (GHA)

ACF plots for weather variables from January 1, 2020 to September 30, 2022

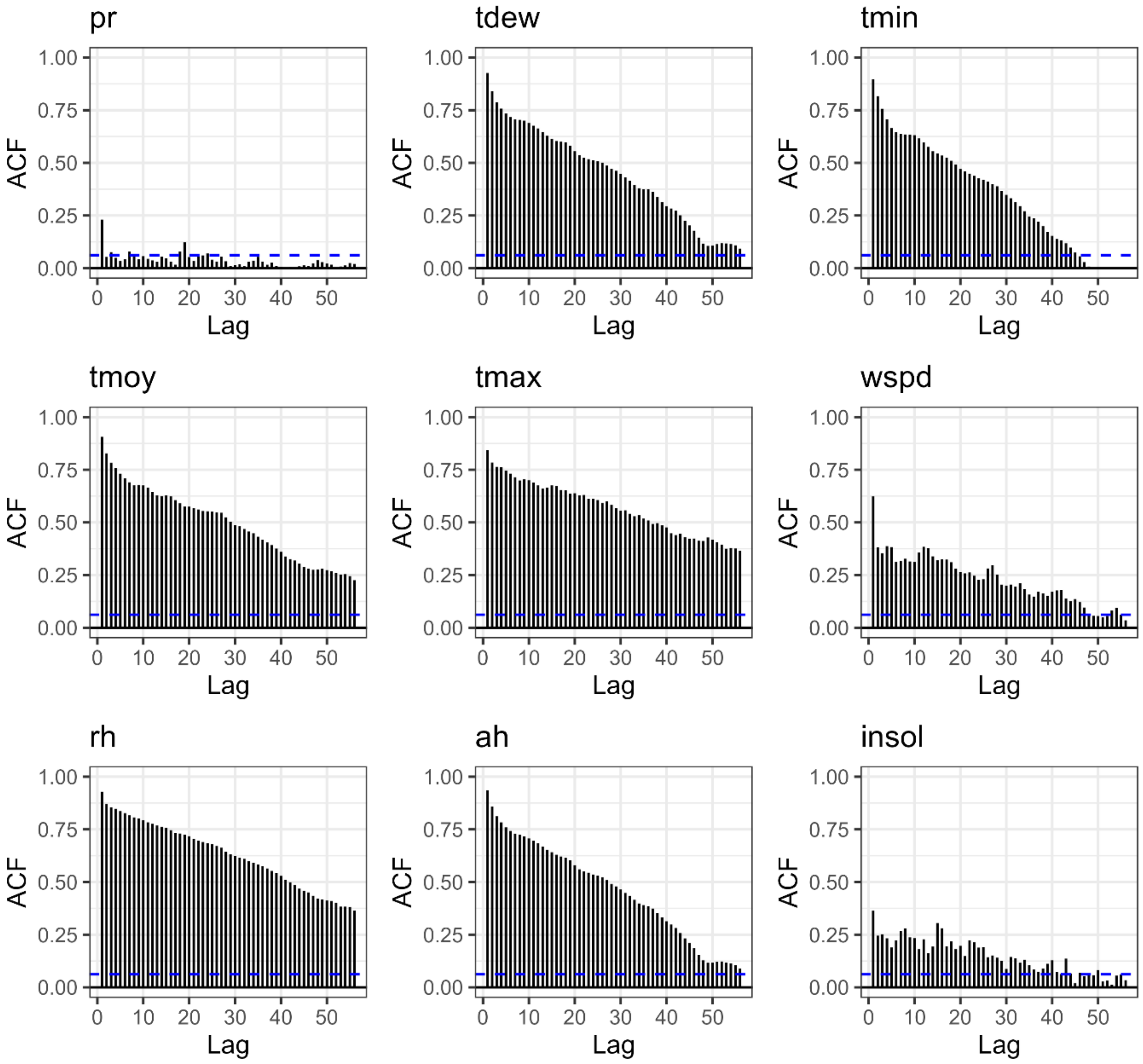

24) Guinea (GIN)

ACF plots for weather variables from January 1, 2020 to September 30, 2022

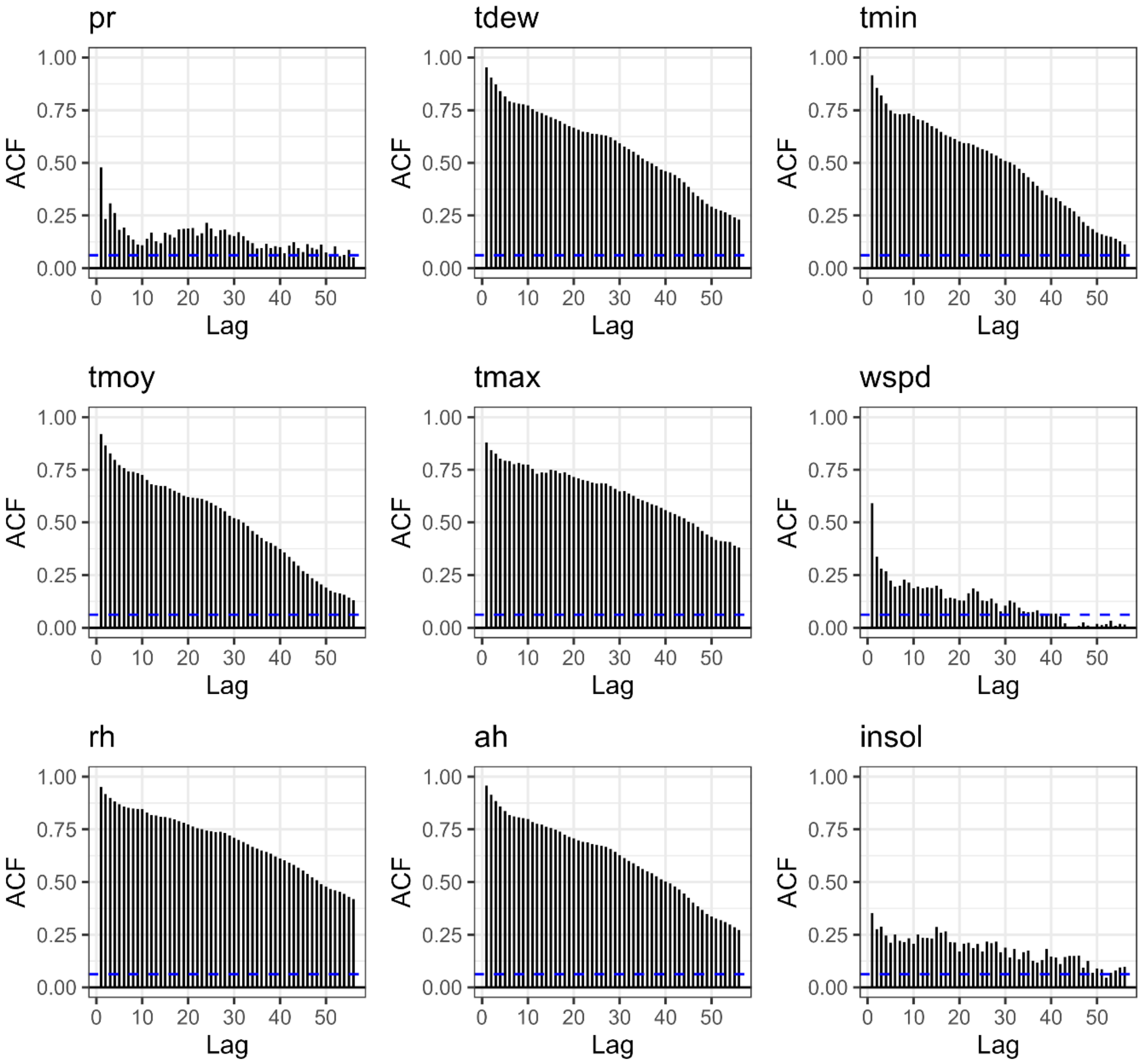

25) Guinea-Bissau (GNB)

ACF plots for weather variables from January 1, 2020 to September 30, 2022

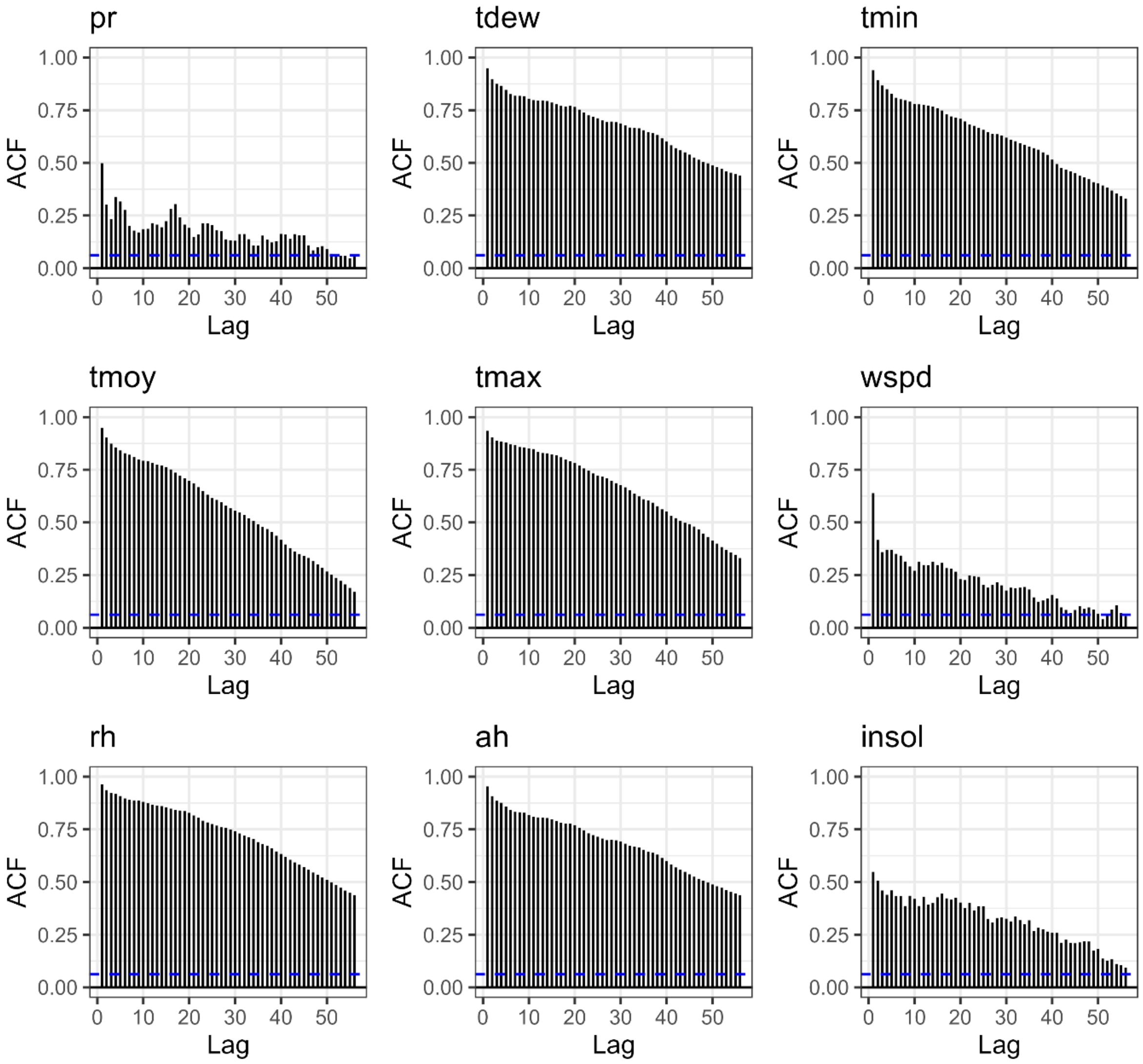

26) Kenya (KEN)

ACF plots for weather variables from January 1, 2020 to September 30, 2022

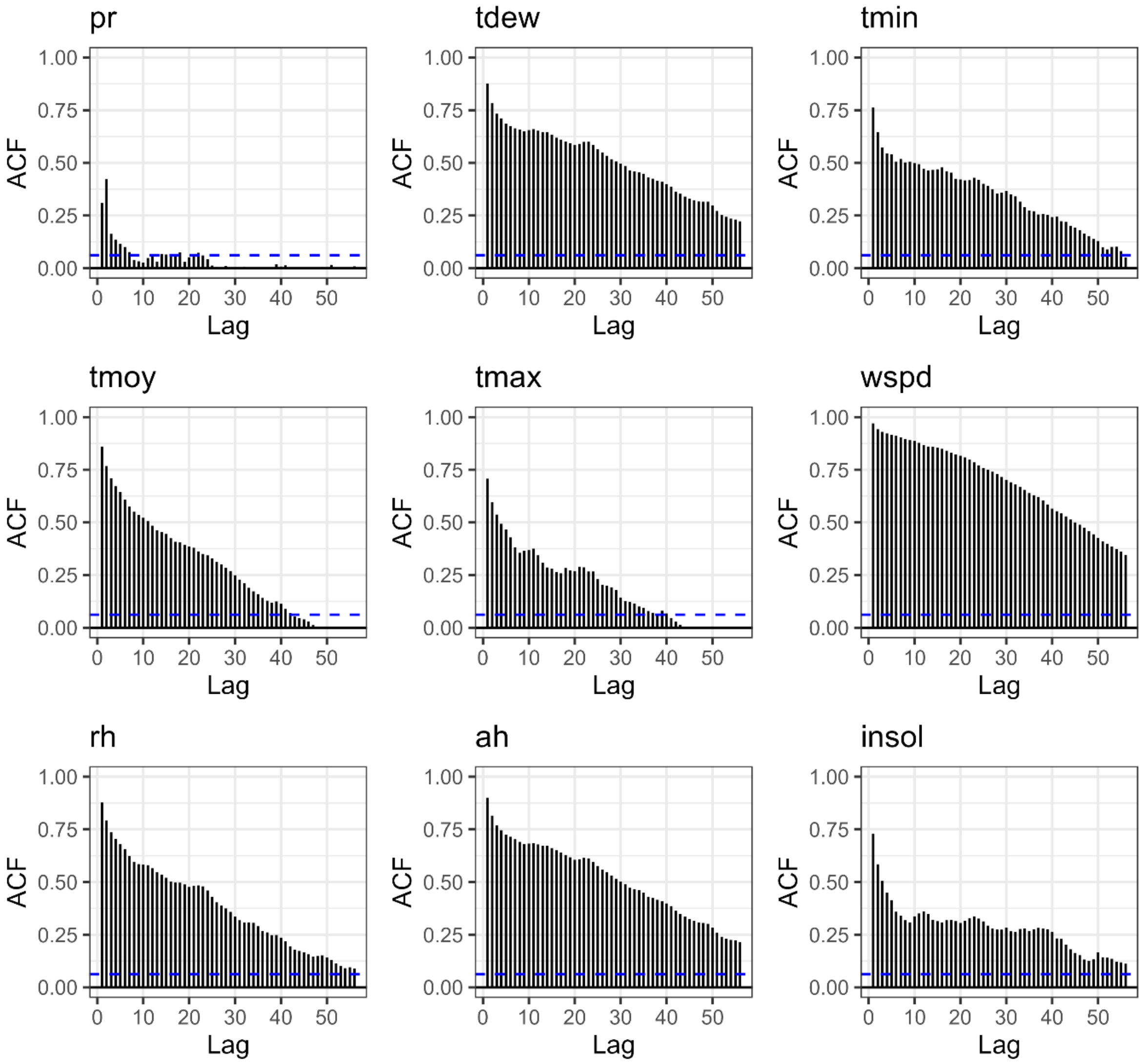

27) Lesotho (LSO)

ACF plots for weather variables from January 1, 2020 to September 30, 2022

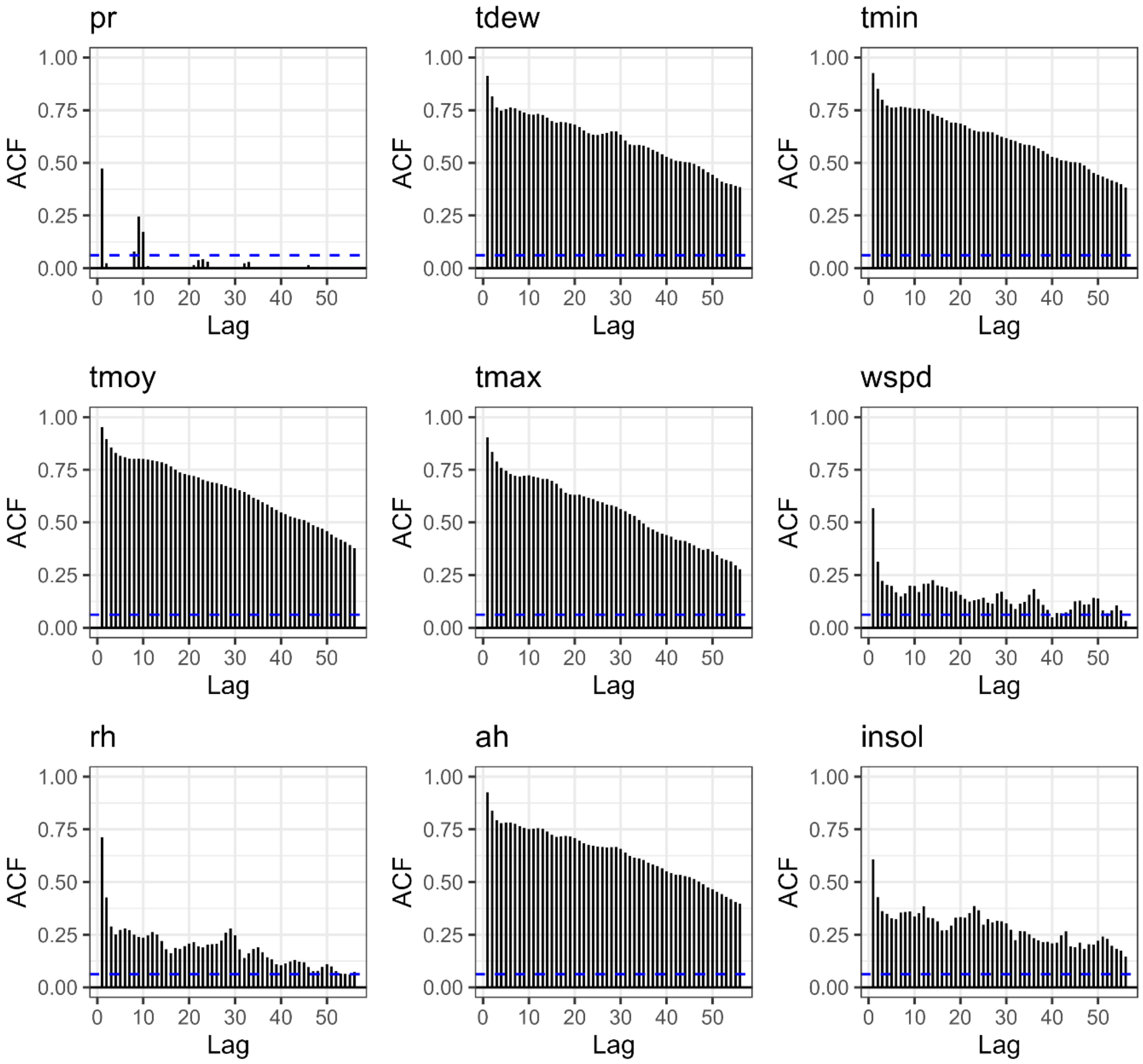

28) Liberia (LBR)

ACF plots for weather variables from January 1, 2020 to September 30, 2022

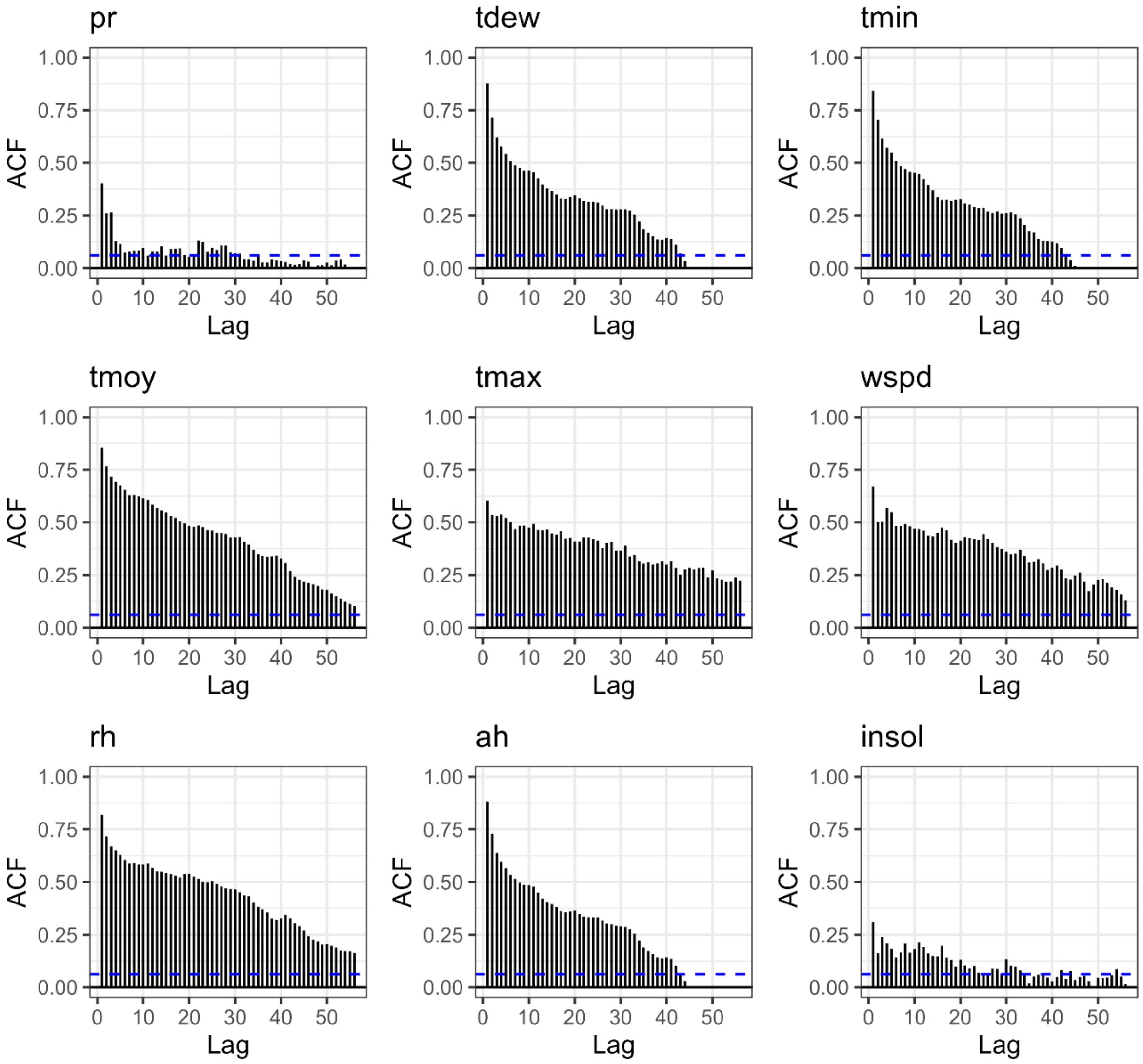

29) Libya (LBY)

ACF plots for weather variables from January 1, 2020 to September 30, 2022

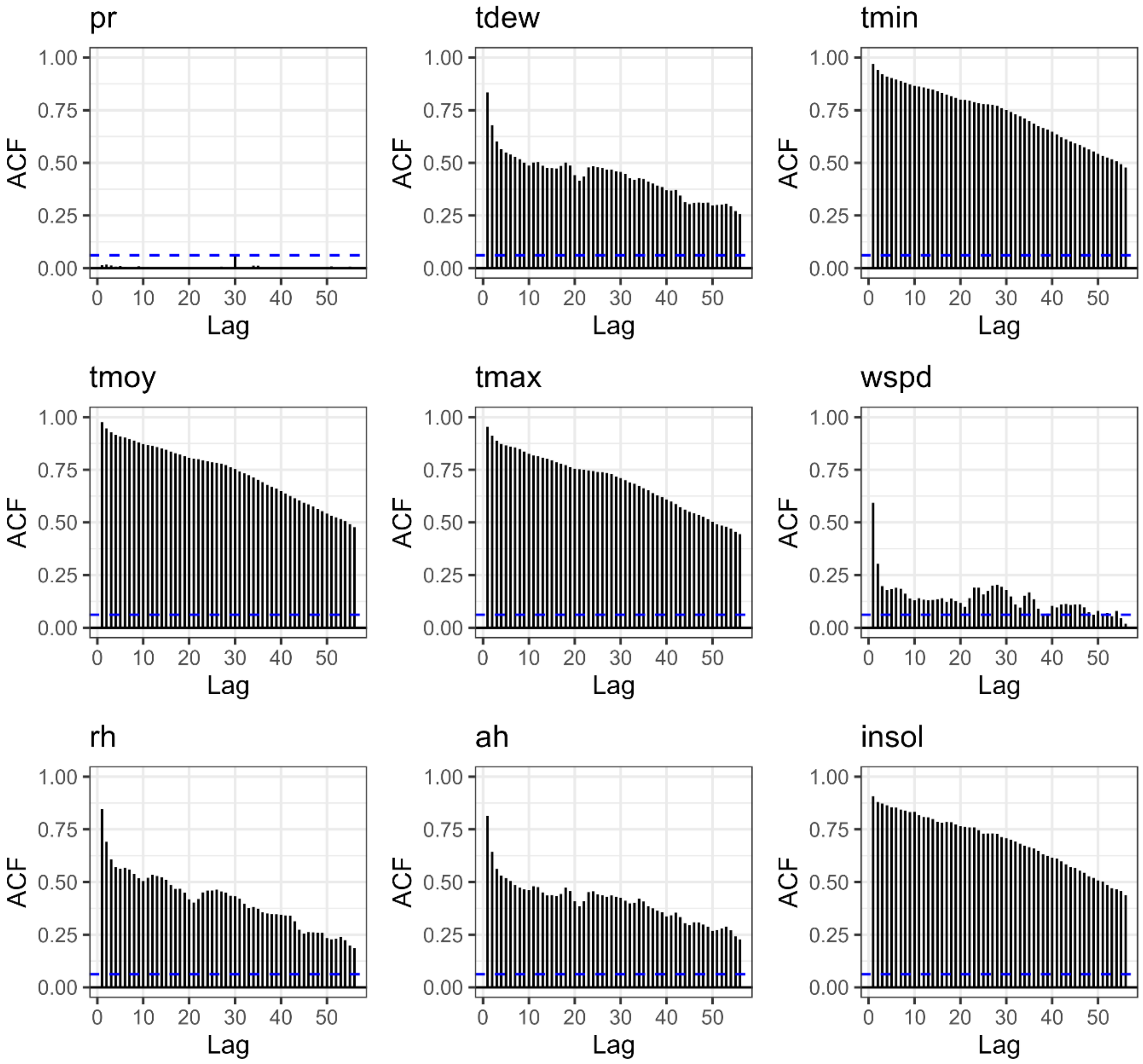

30) Madagascar (MDG)

ACF plots for weather variables from January 1, 2020 to September 30, 2022

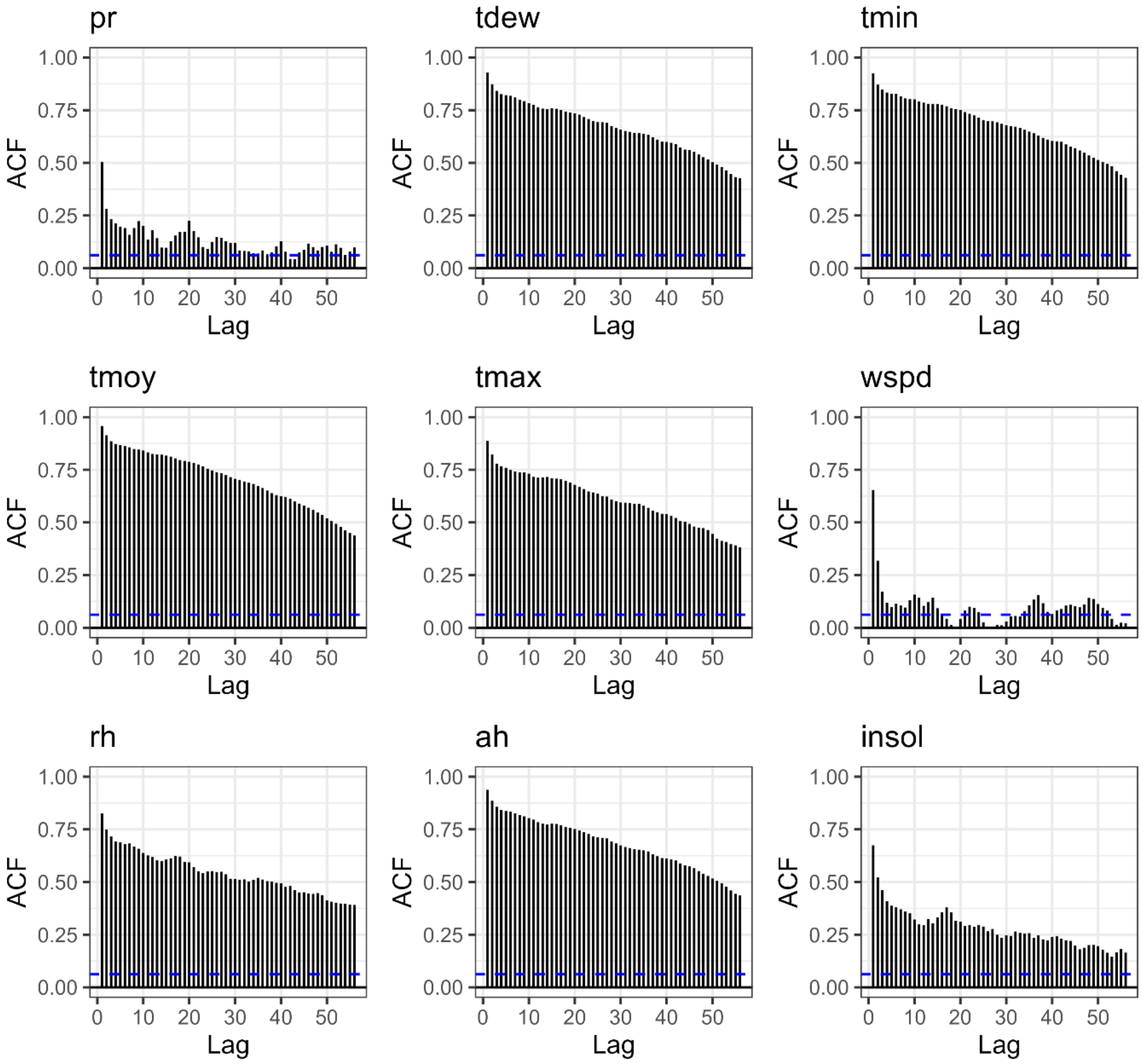

31) Malawi (MWI)

ACF plots for weather variables from January 1, 2020 to September 30, 2022

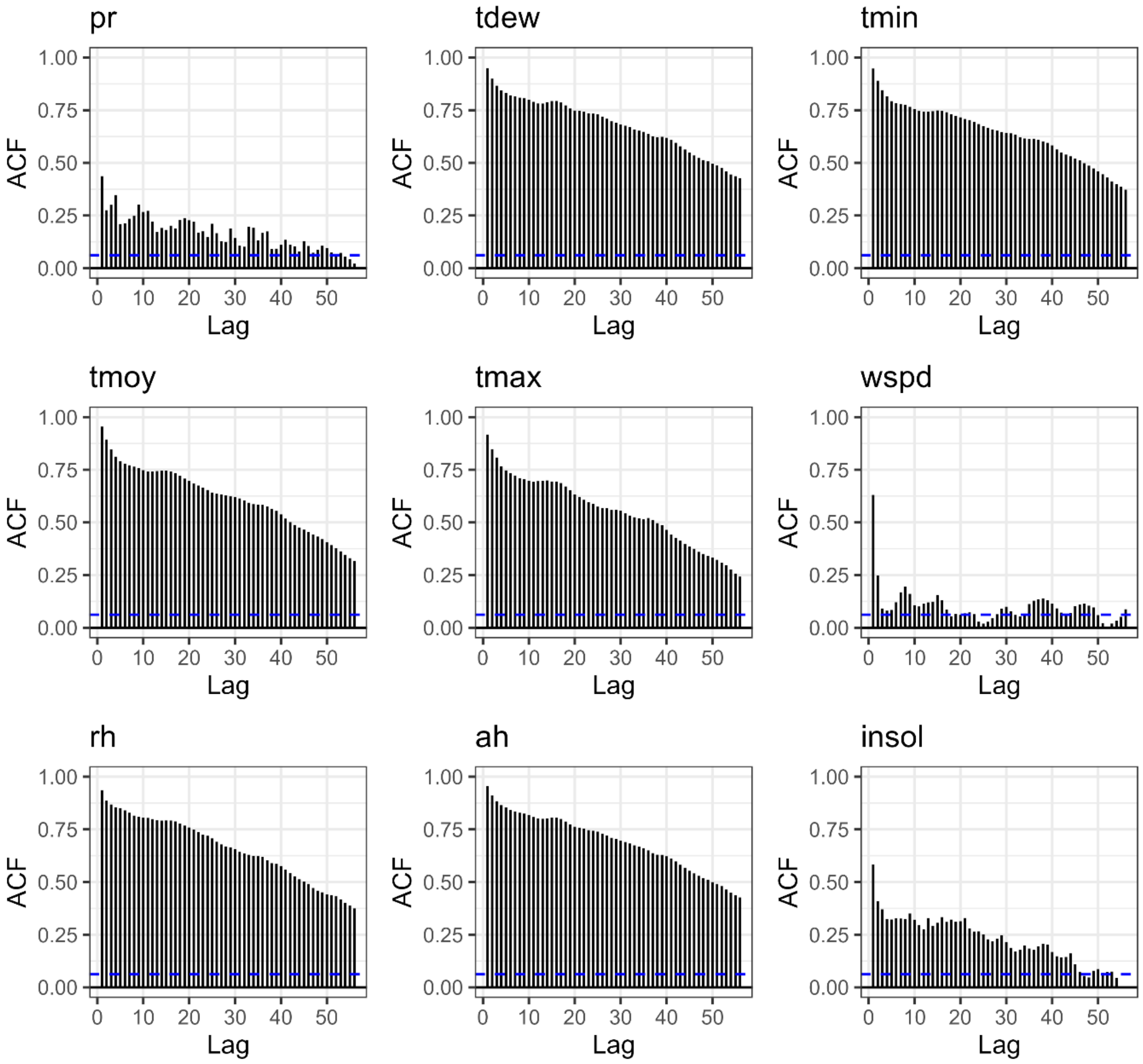

32) Mali (MLI)

ACF plots for weather variables from January 1, 2020 to September 30, 2022

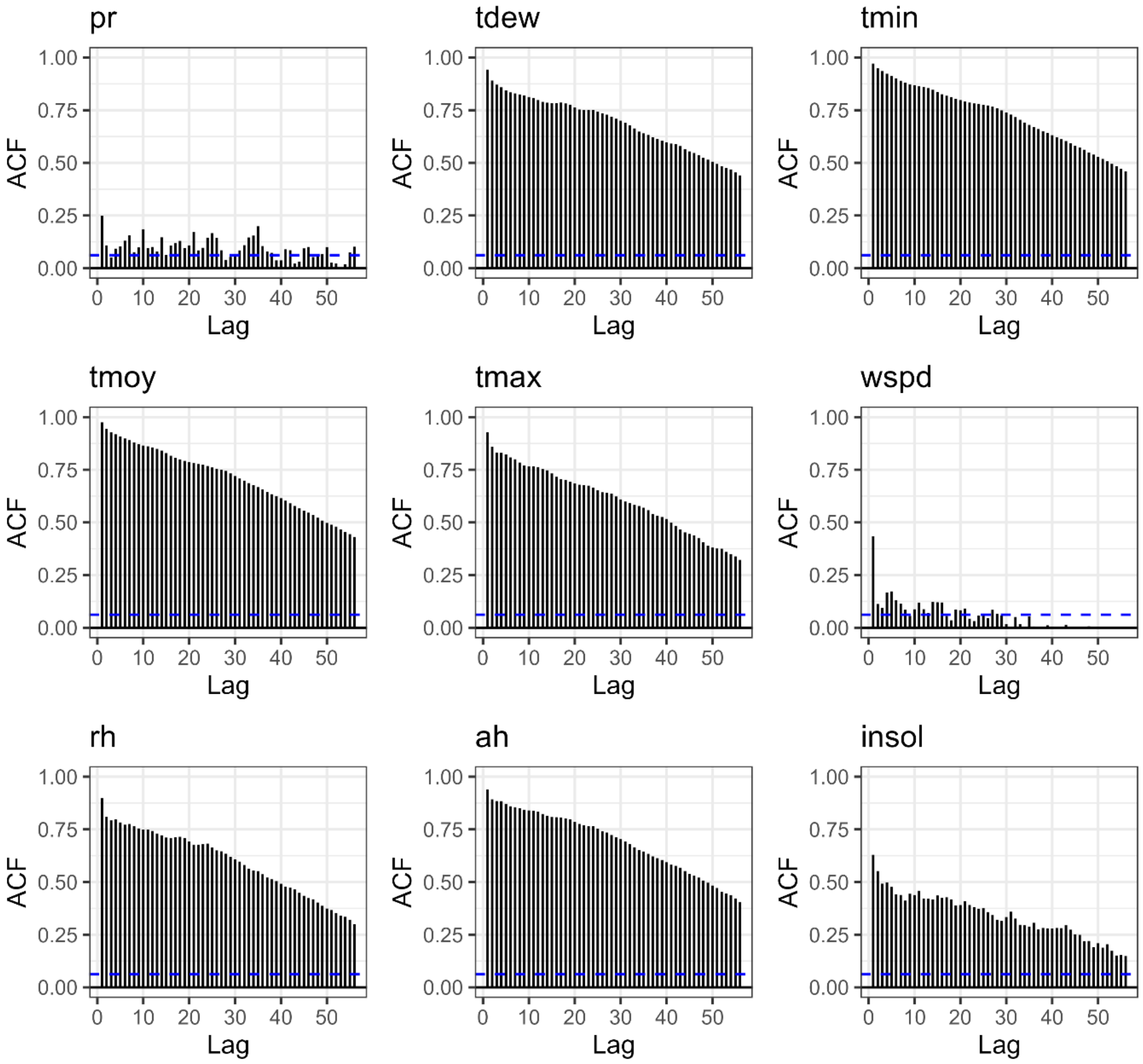

33) Mauritania (MRT)

ACF plots for weather variables from January 1, 2020 to September 30, 2022

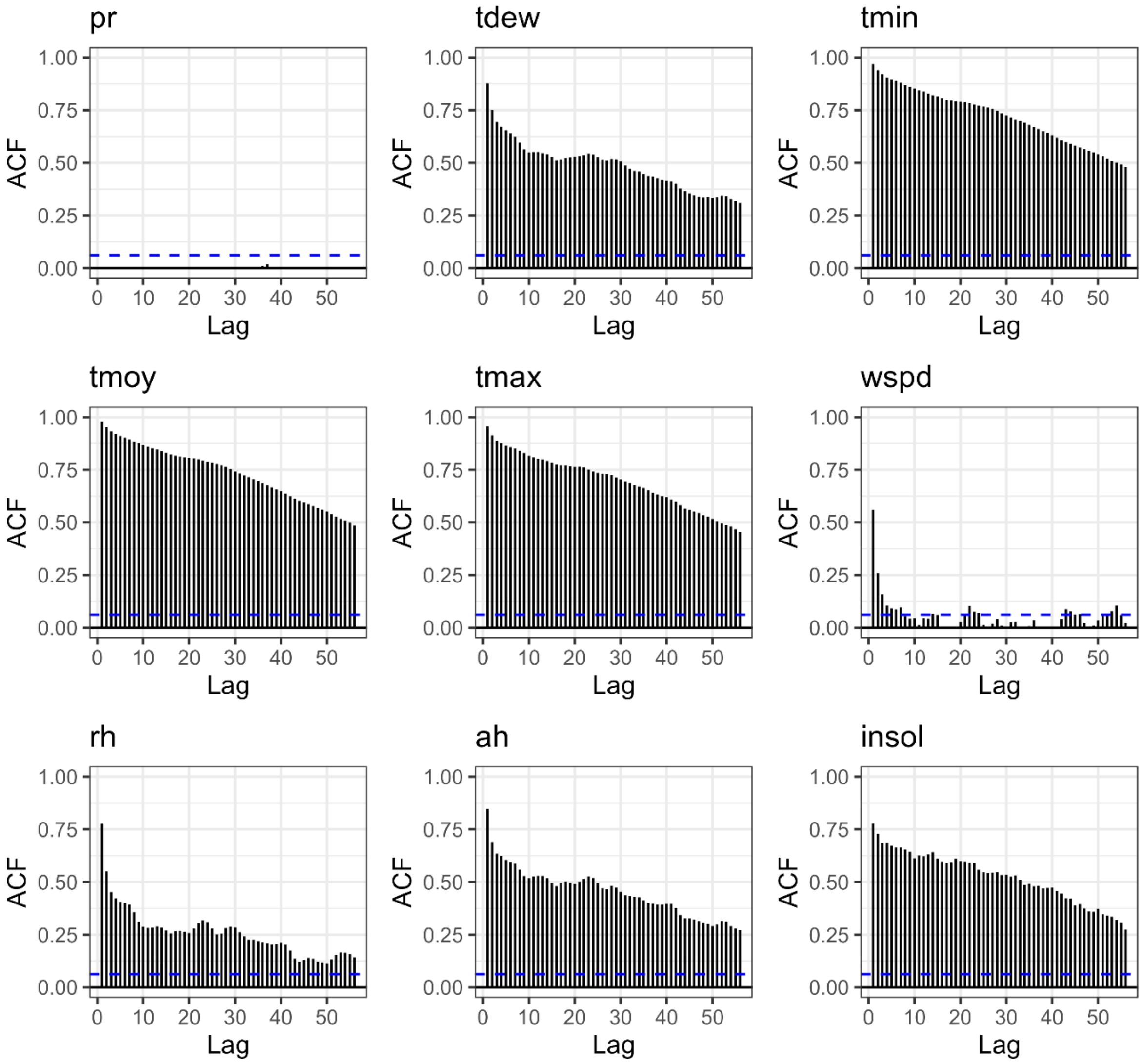

34) Mauritius (MUS)

ACF plots for weather variables from January 1, 2020 to September 30, 2022

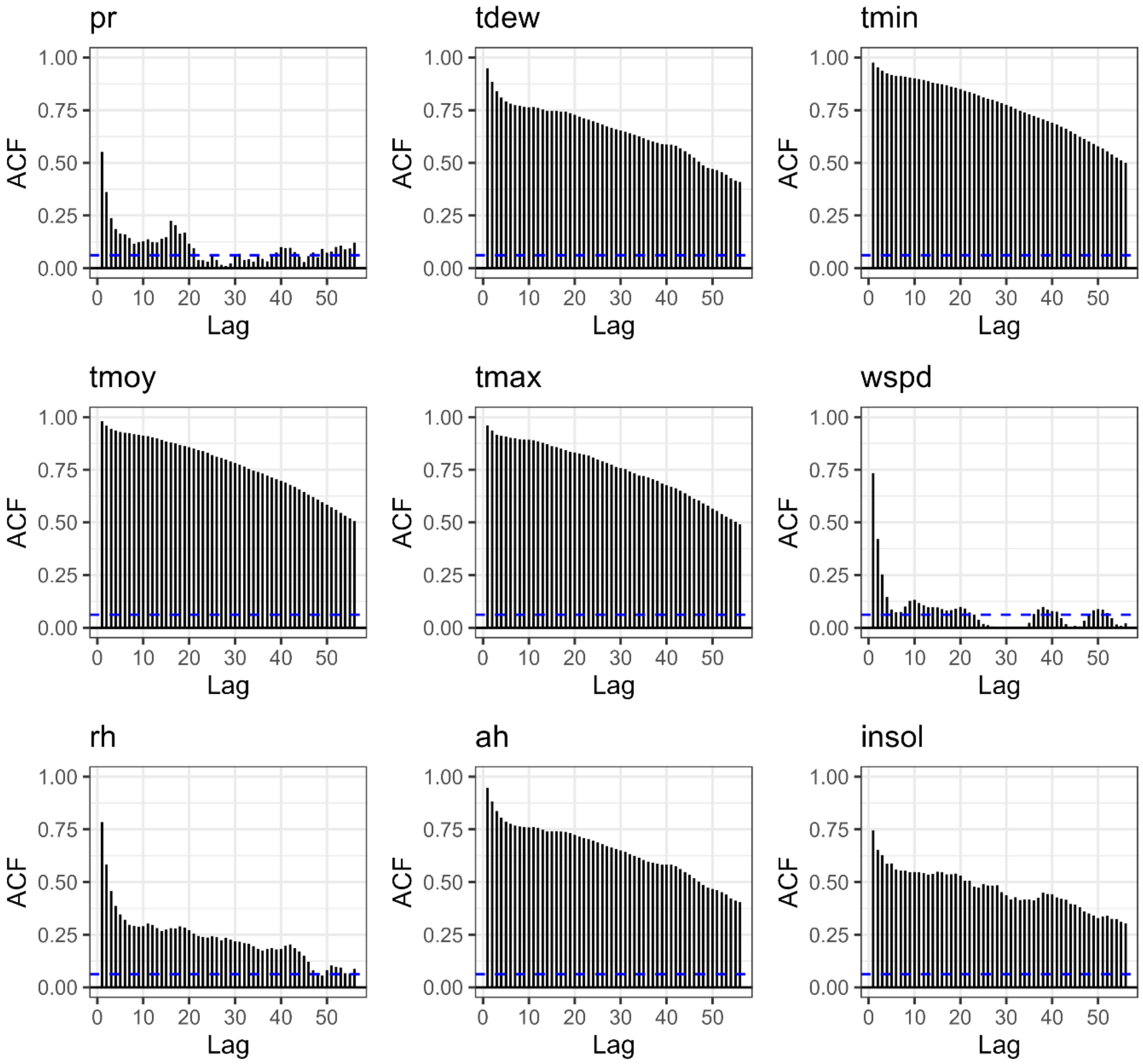

35) Morocco (MAR)

ACF plots for weather variables from January 1, 2020 to September 30, 2022

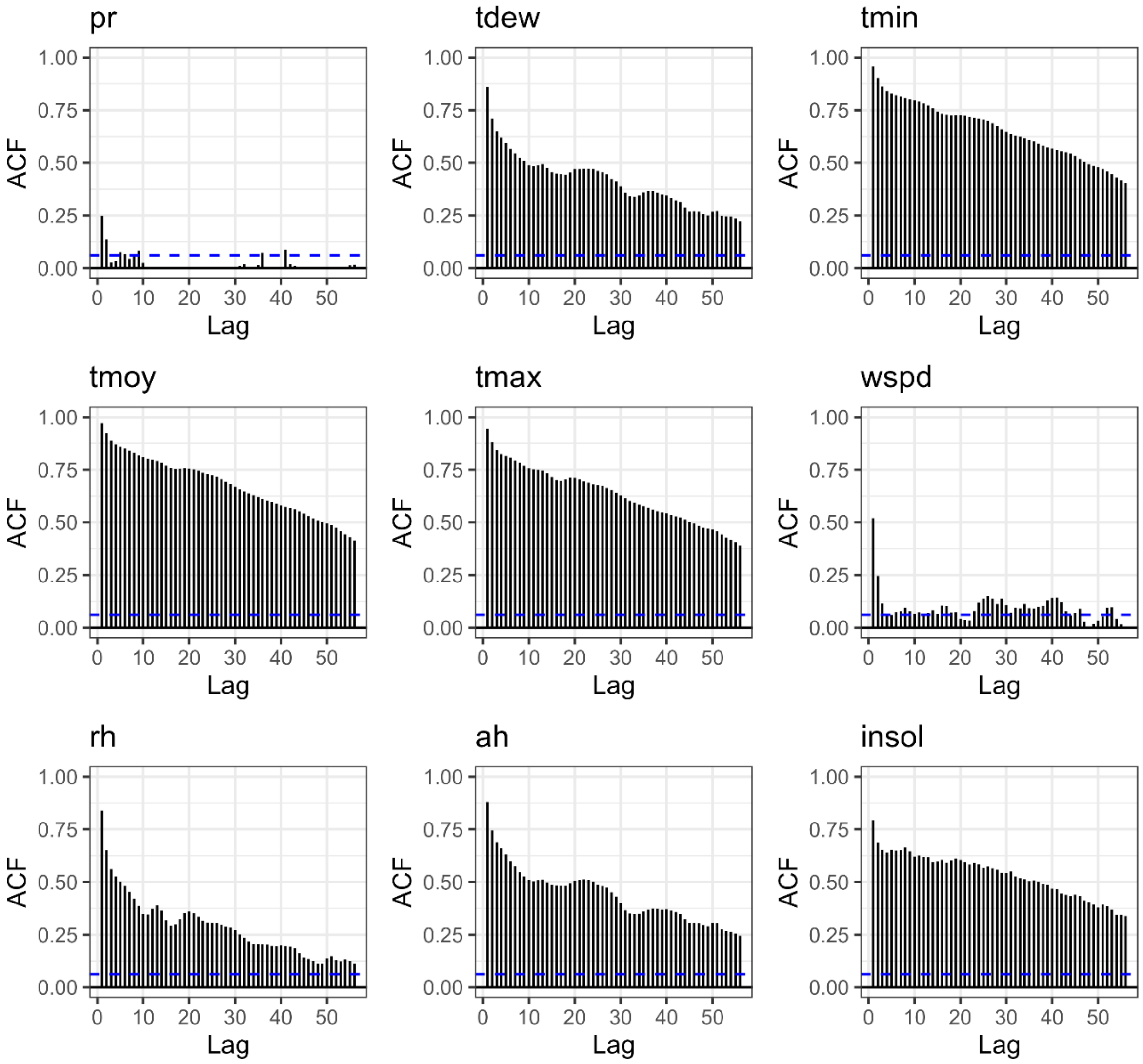

36) Mozambique (MOZ)

ACF plots for weather variables from January 1, 2020 to September 30, 2022

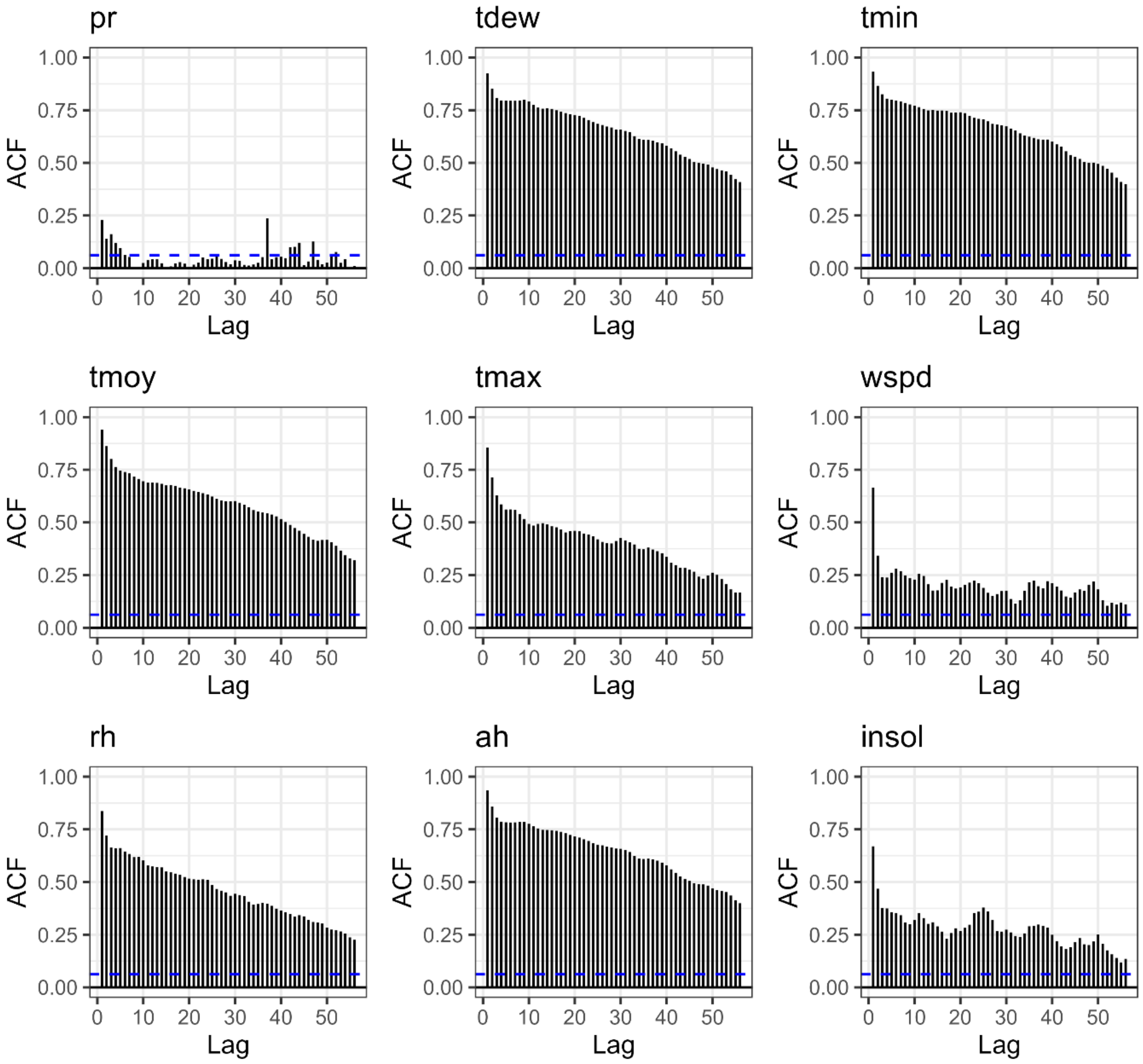

37) Namibia (NAM)

ACF plots for weather variables from January 1, 2020 to September 30, 2022

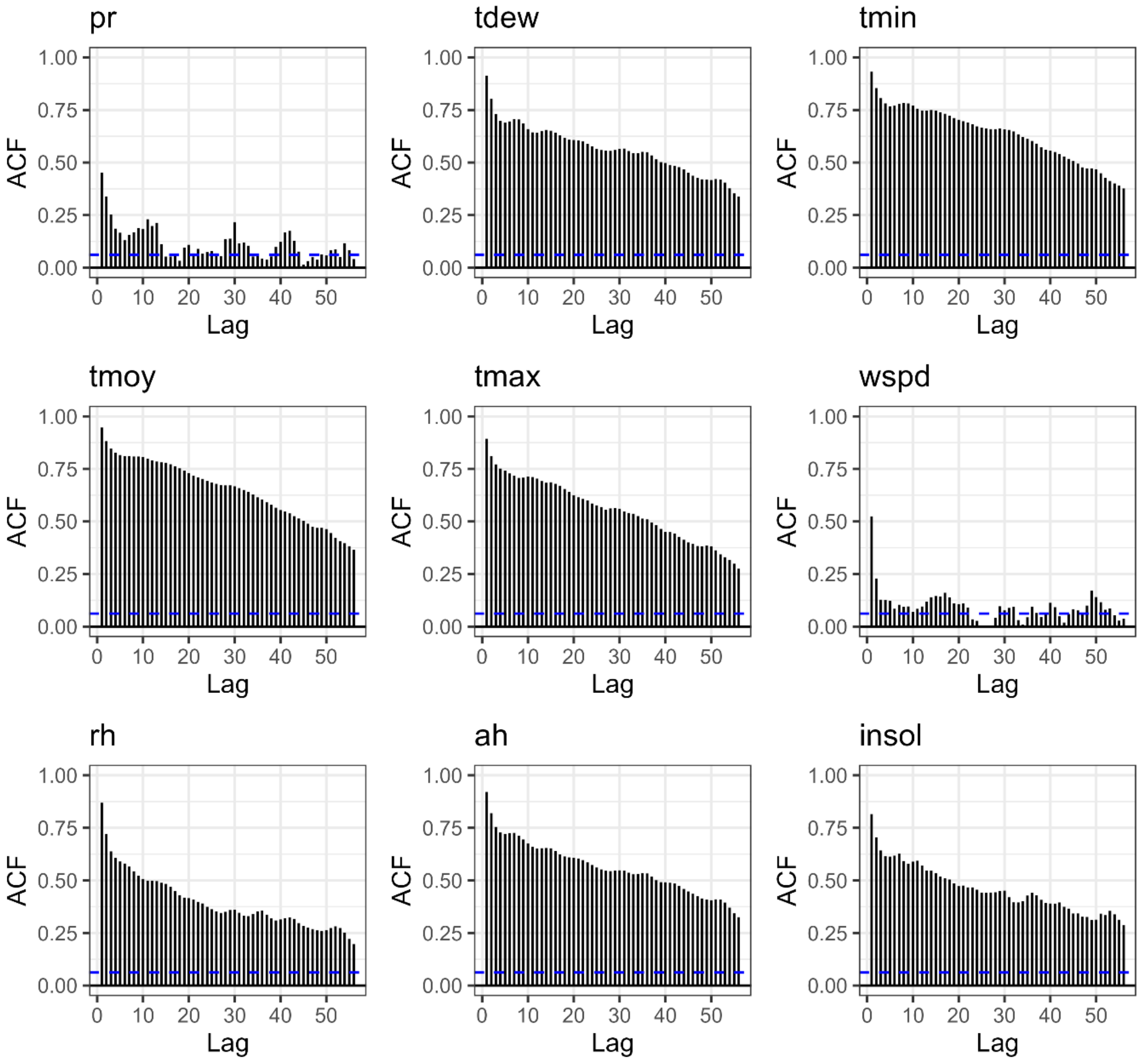

38) Niger (NER)

ACF plots for weather variables from January 1, 2020 to September 30, 2022

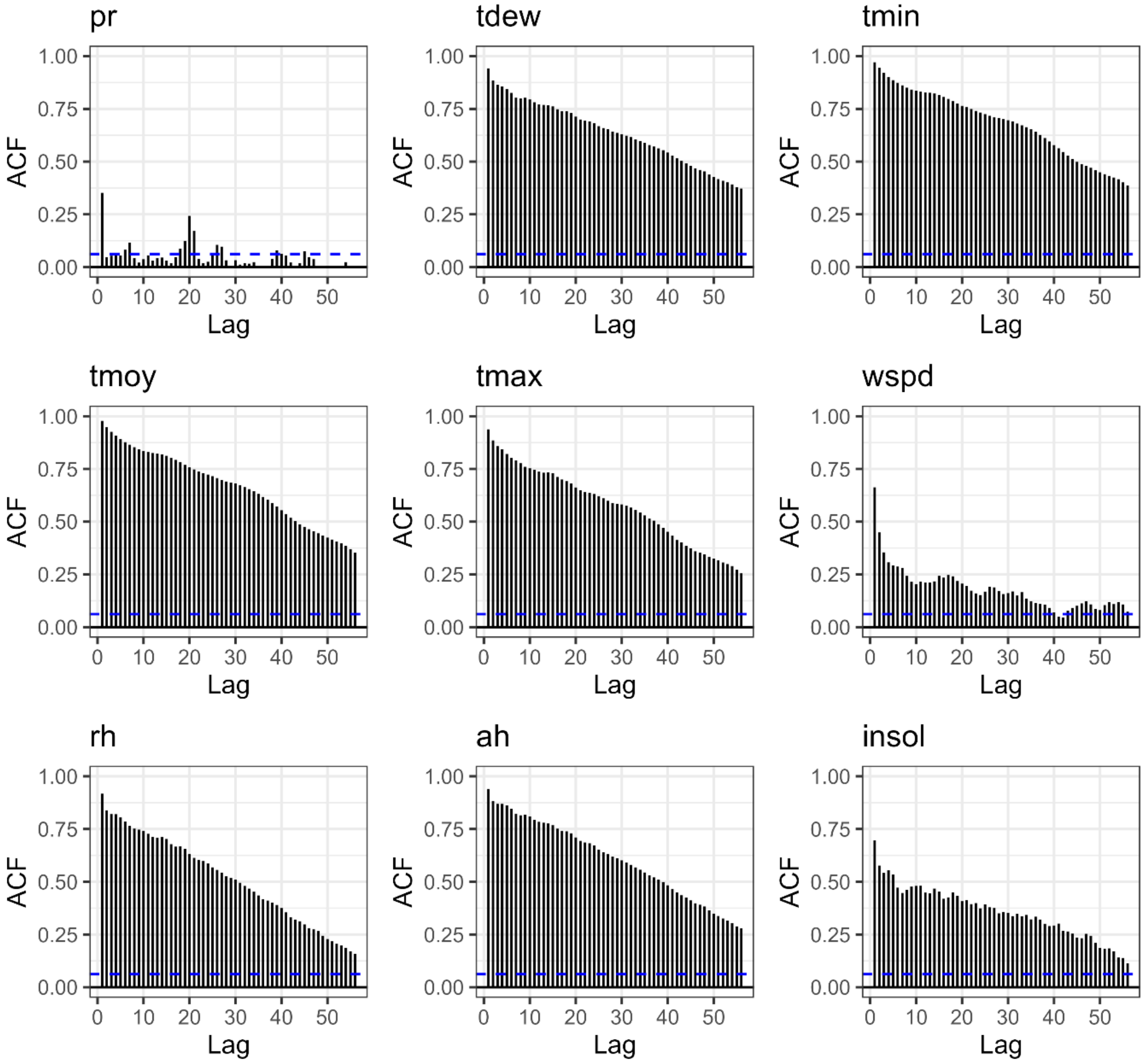

39) Nigeria (NGA)
ACF plots for weather variables from January 1, 2020 to September 30, 2022

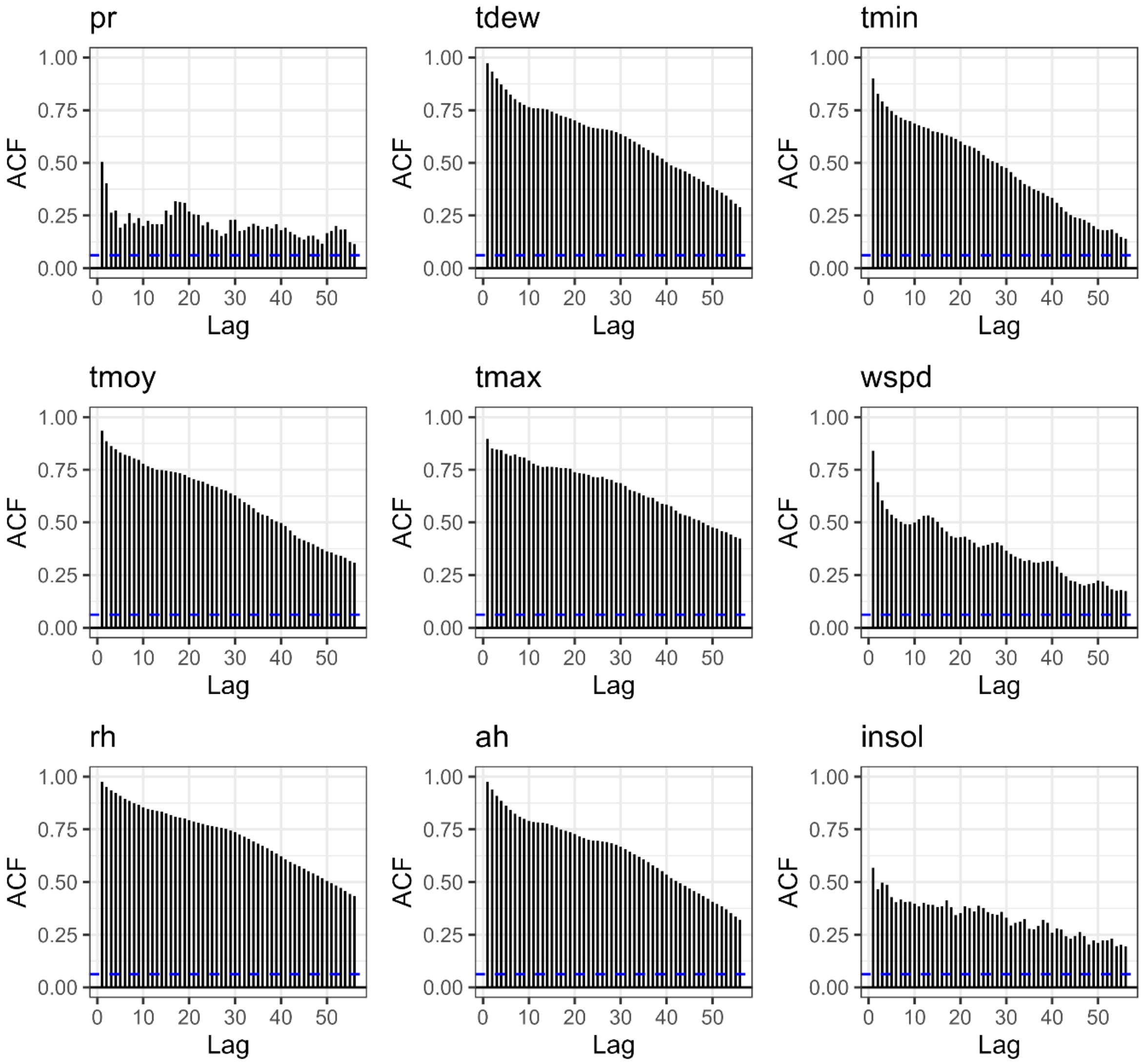

40) Rwanda (RWA)

ACF plots for weather variables from January 1, 2020 to September 30, 2022

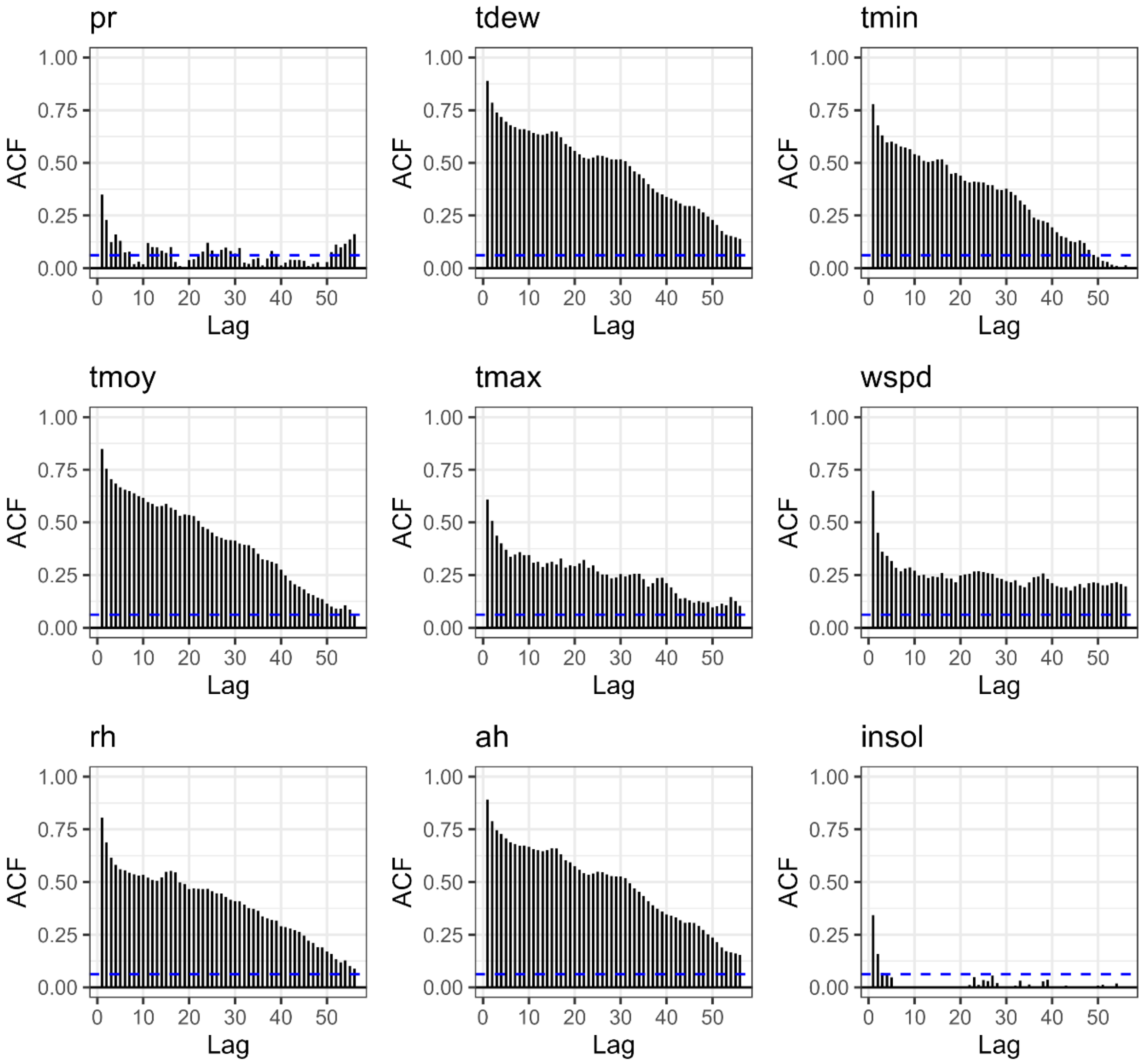

41) Sao Tome and Principe (STP)

ACF plots for weather variables from January 1, 2020 to September 30, 2022

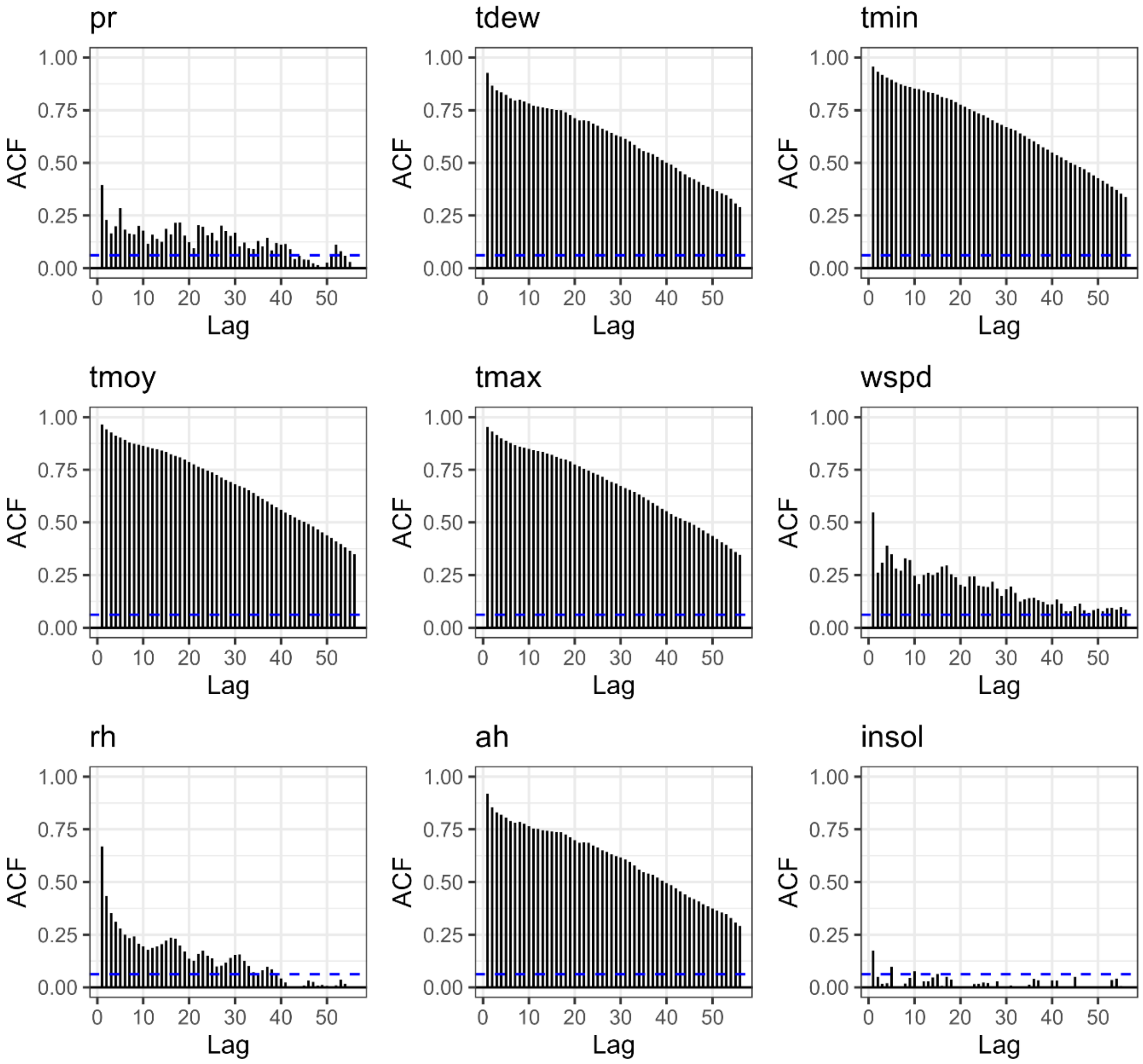

42) Senegal (SEN)

ACF plots for weather variables from January 1, 2020 to September 30, 2022

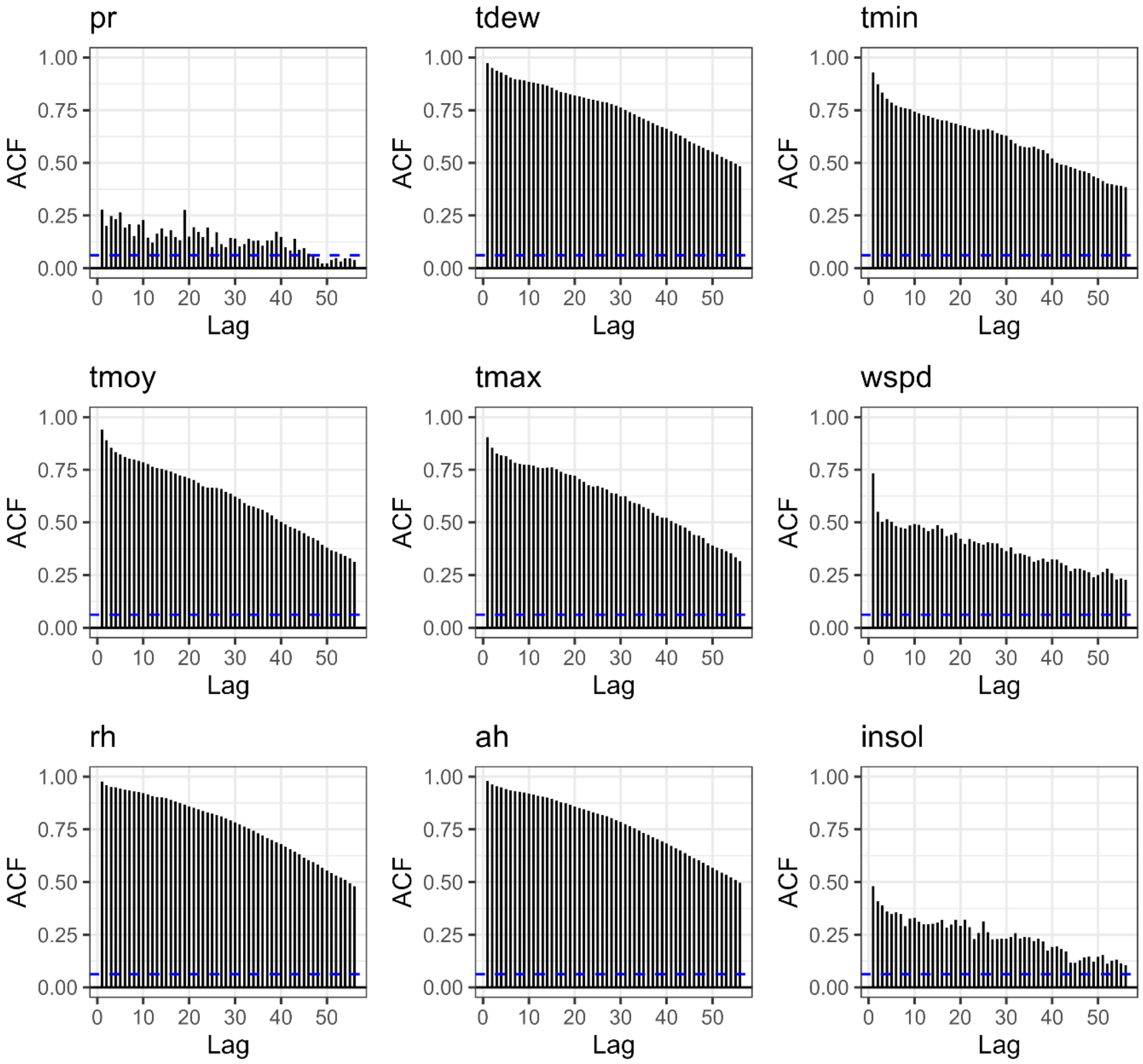

43) Seychelles (SYC)

ACF plots for weather variables from January 1, 2020 to September 30, 2022

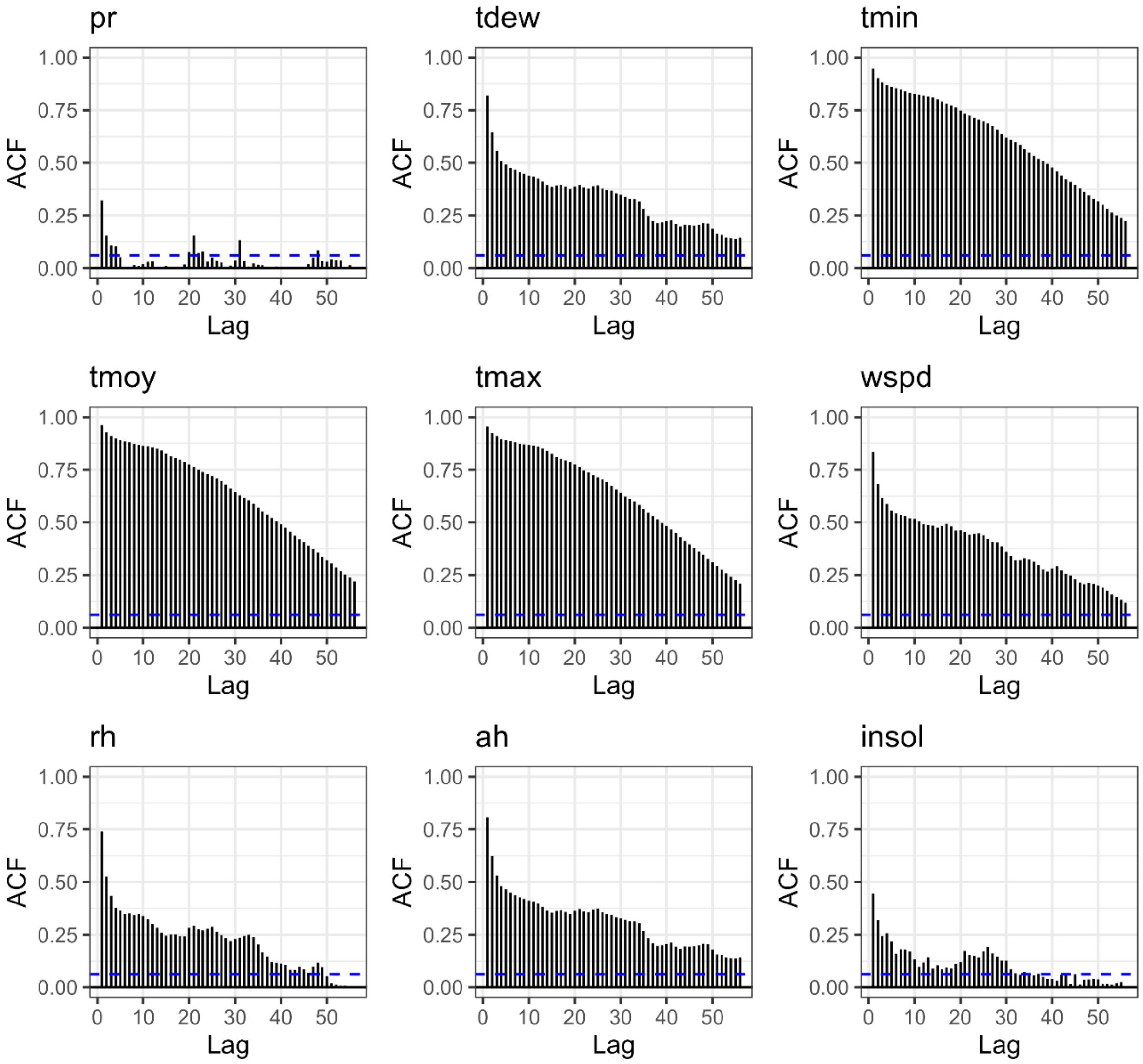

44) Sierra Leone (SLE)

ACF plots for weather variables from January 1, 2020 to September 30, 2022

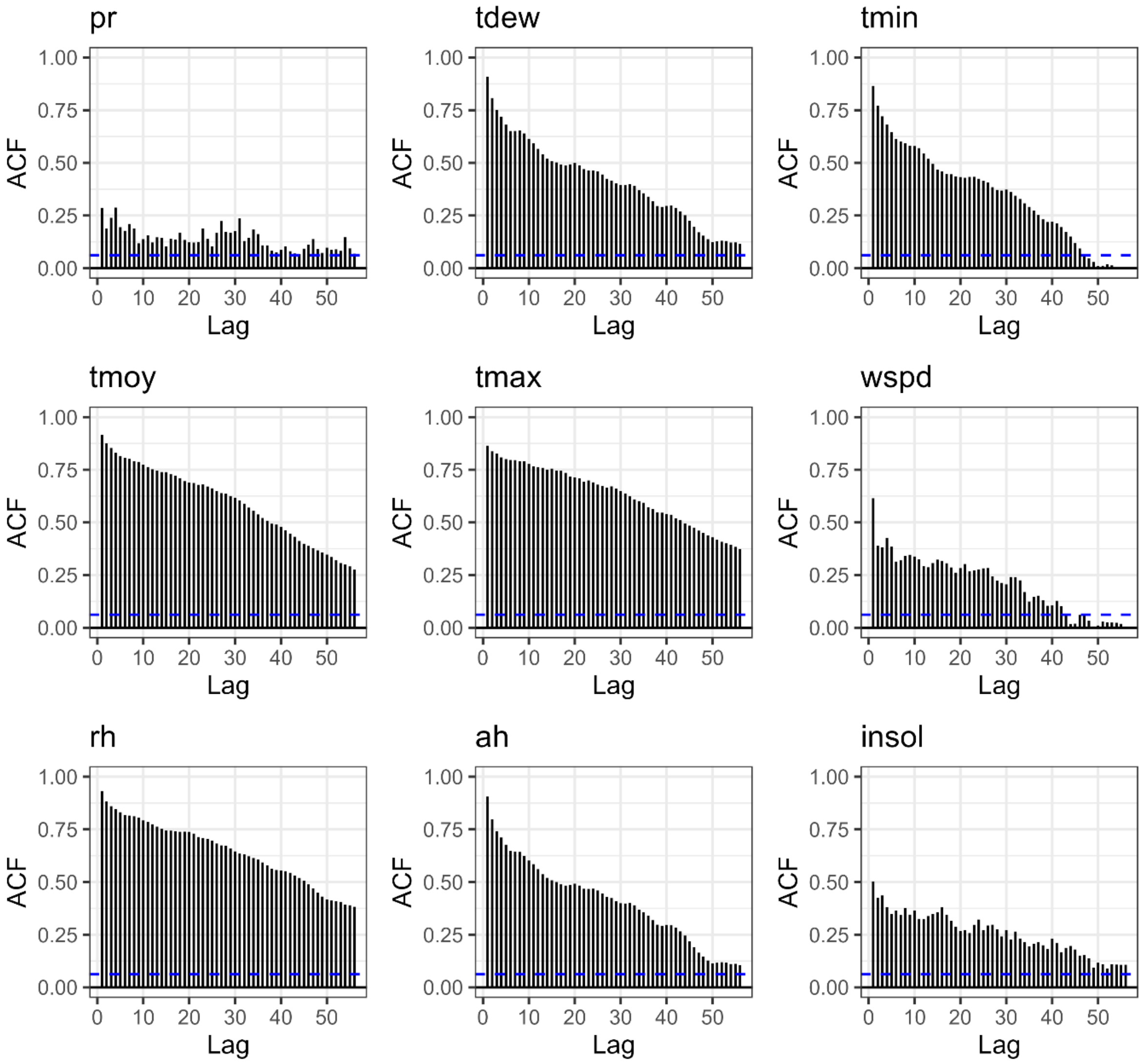

45) Somalia (SOM)

ACF plots for weather variables from January 1, 2020 to September 30, 2022

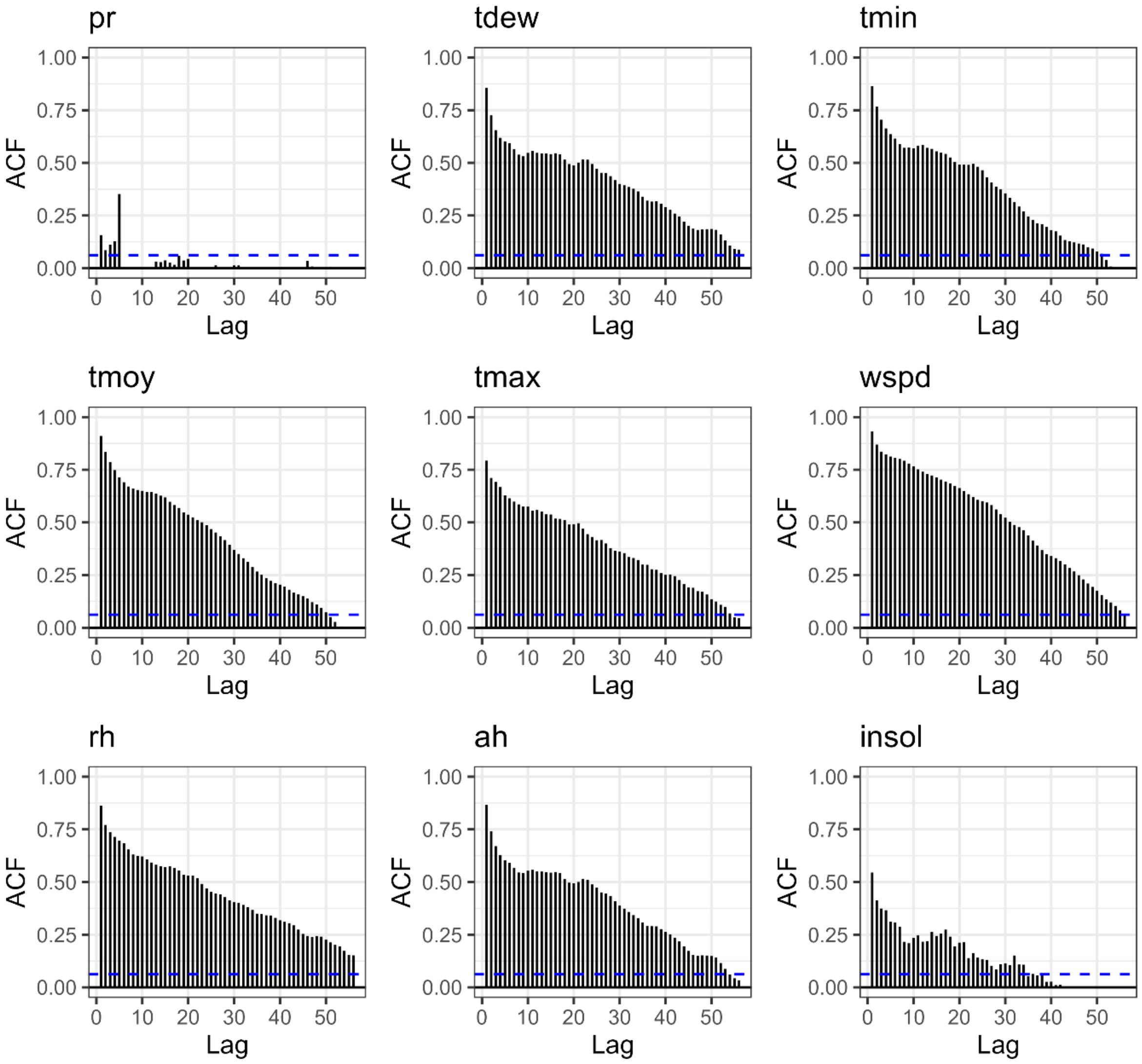

46) South Africa (ZAF)

ACF plots for weather variables from January 1, 2020 to September 30, 2022

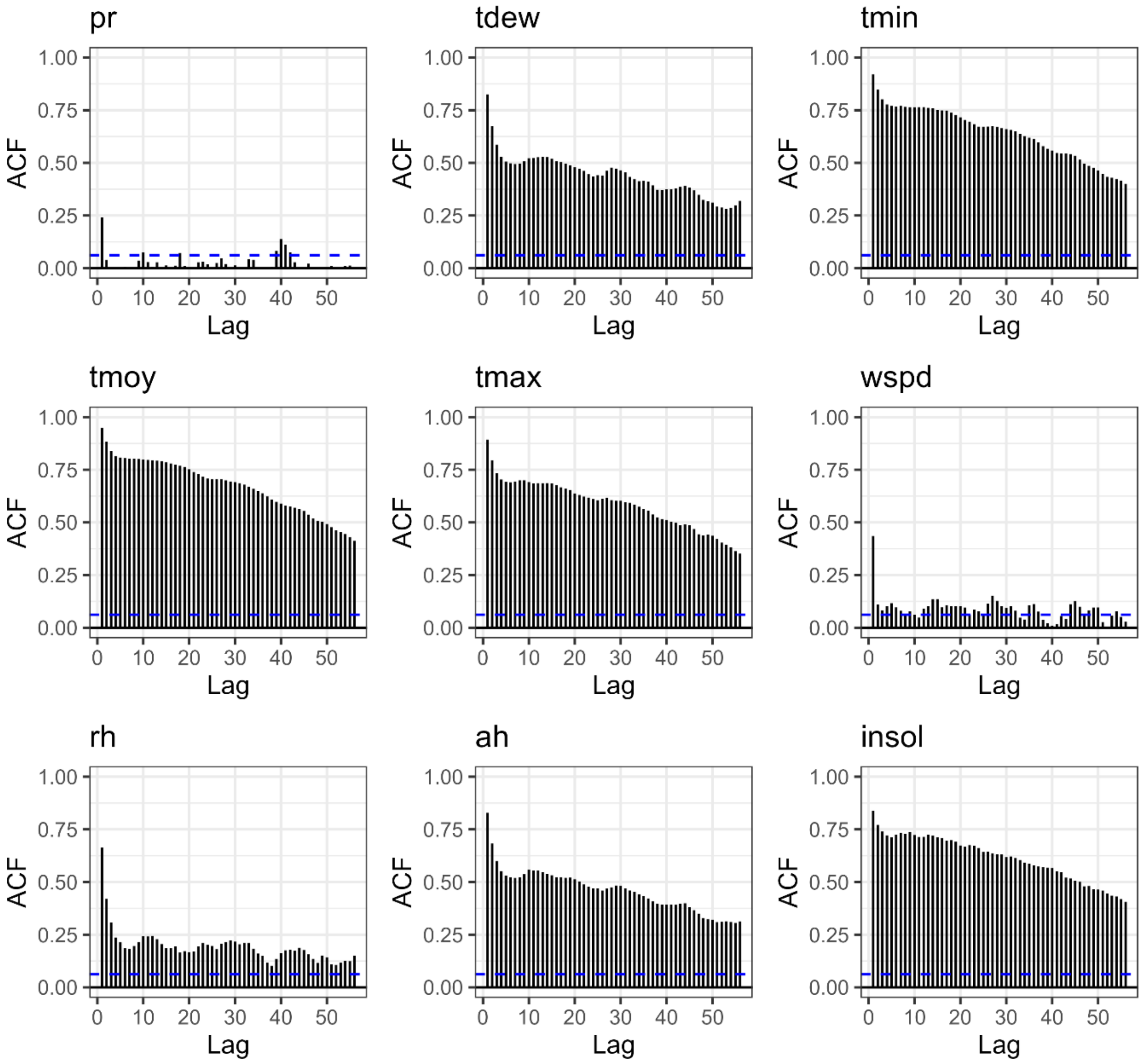

47) South Sudan (SSD)

ACF plots for weather variables from January 1, 2020 to September 30, 2022

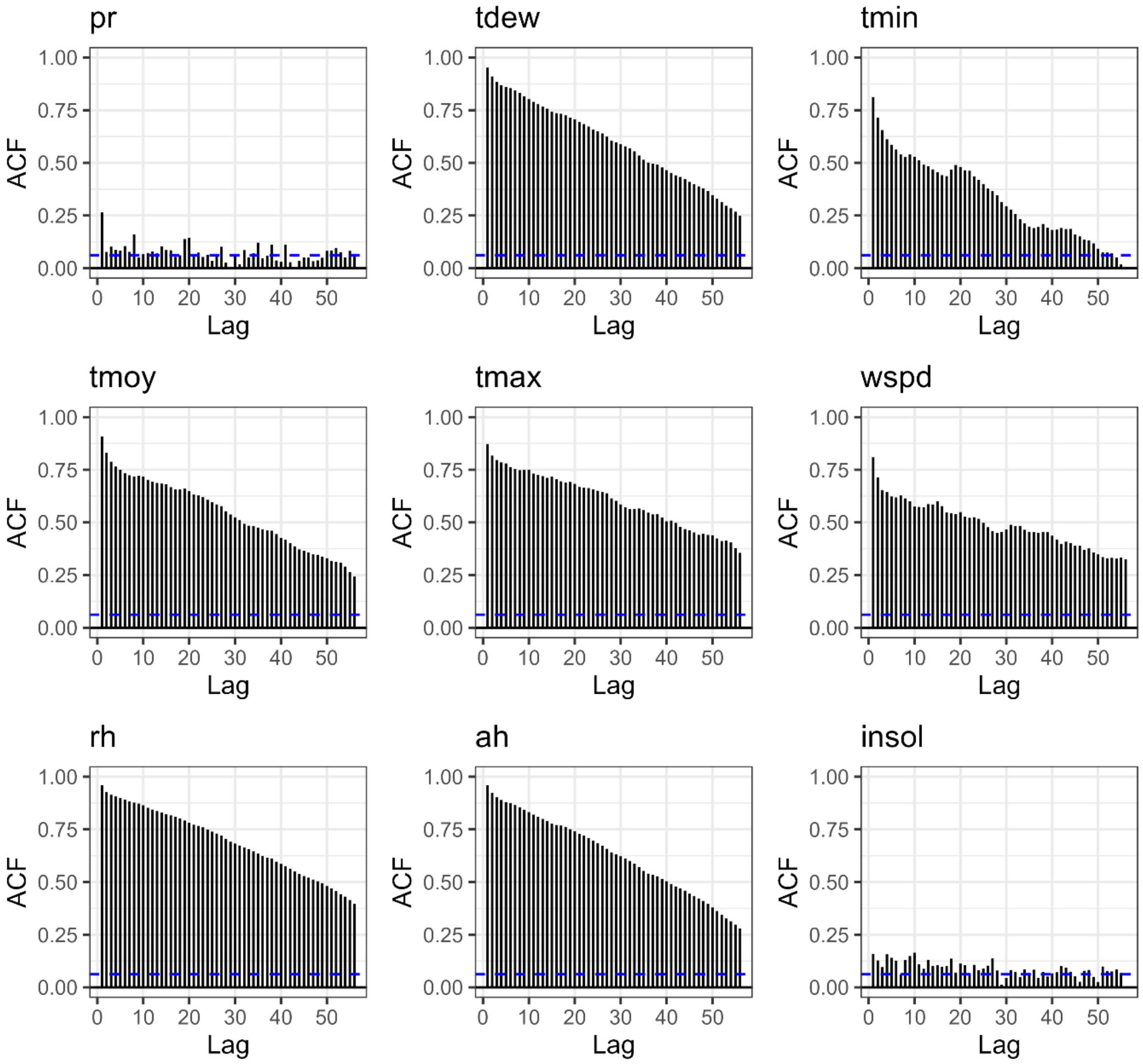

48) Sudan (SDN)

ACF plots for weather variables from January 1, 2020 to September 30, 2022

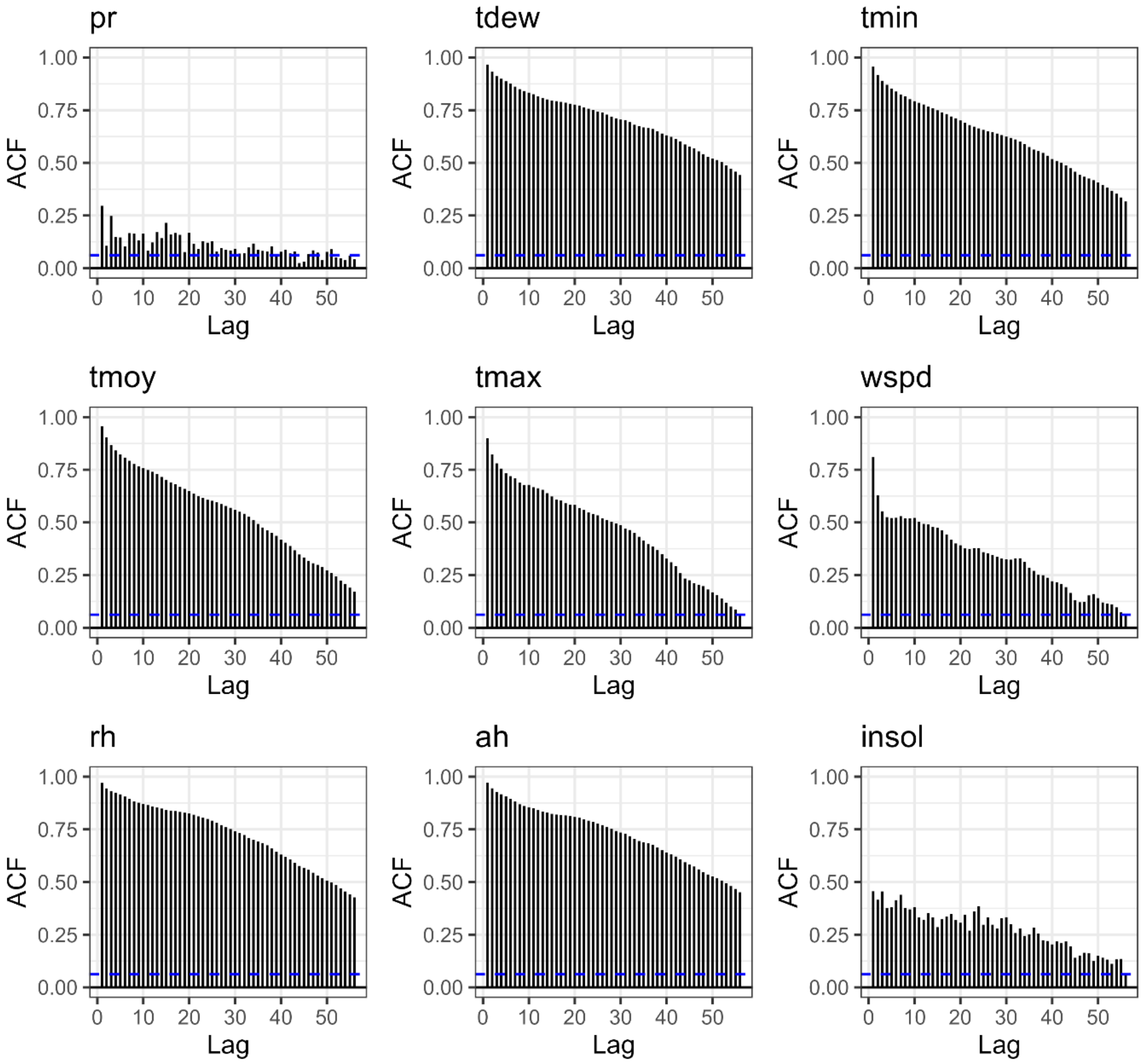

49) Tanzania (TZA)

ACF plots for weather variables from January 1, 2020 to September 30, 2022

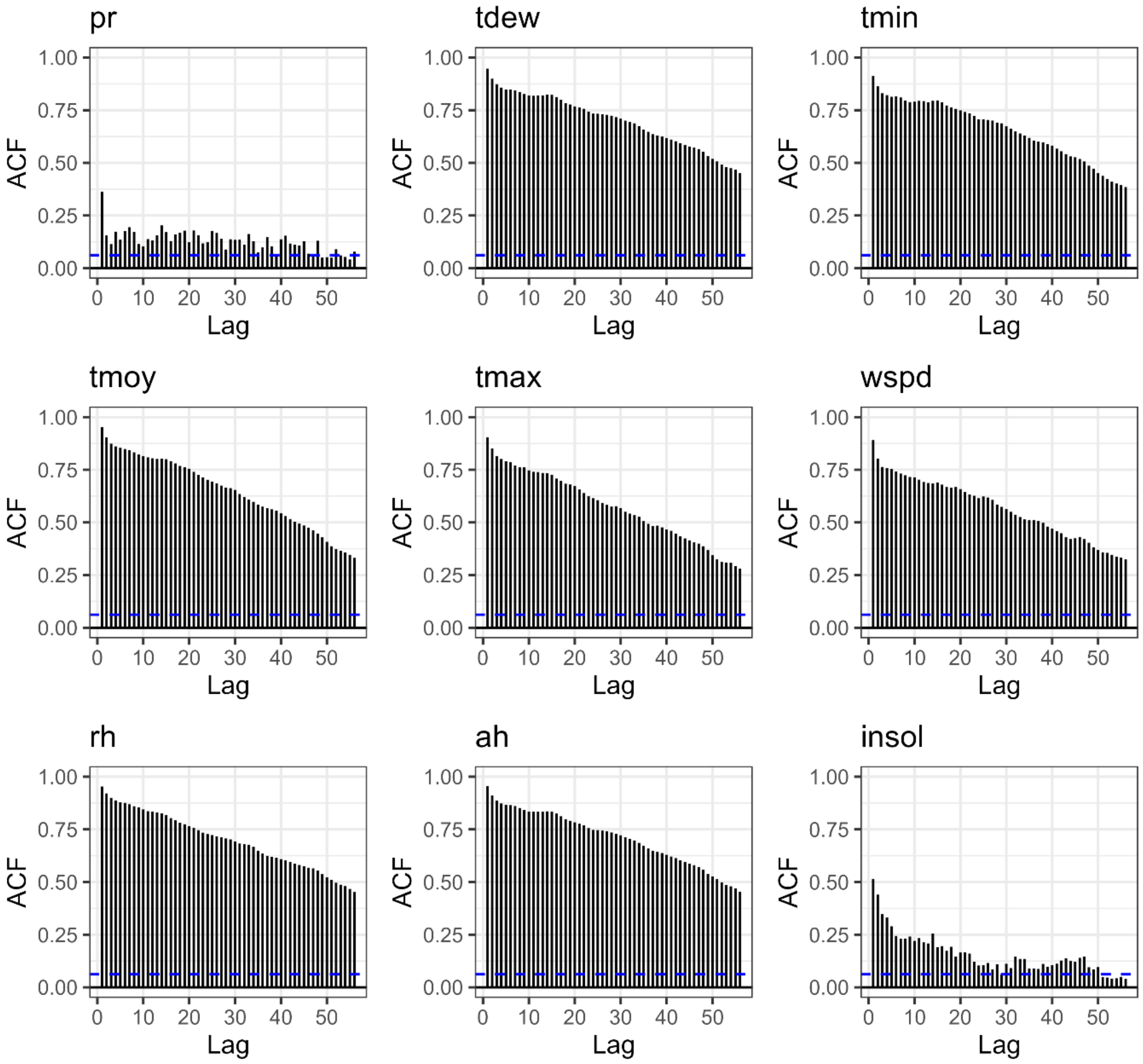

50) Togo (TGO)

ACF plots for weather variables from January 1, 2020 to September 30, 2022

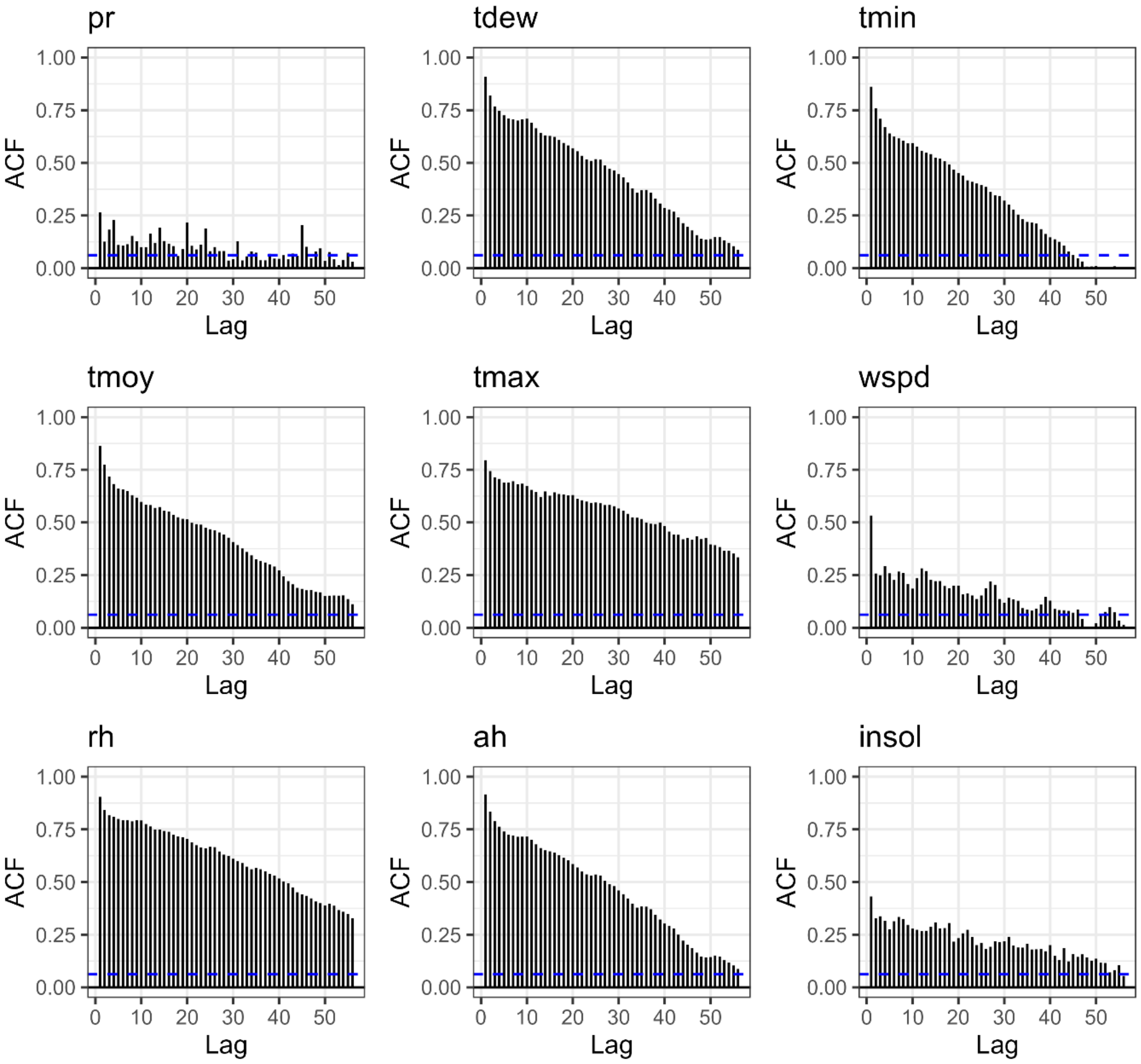

51) Tunisia (TUN)

ACF plots for weather variables from January 1, 2020 to September 30, 2022

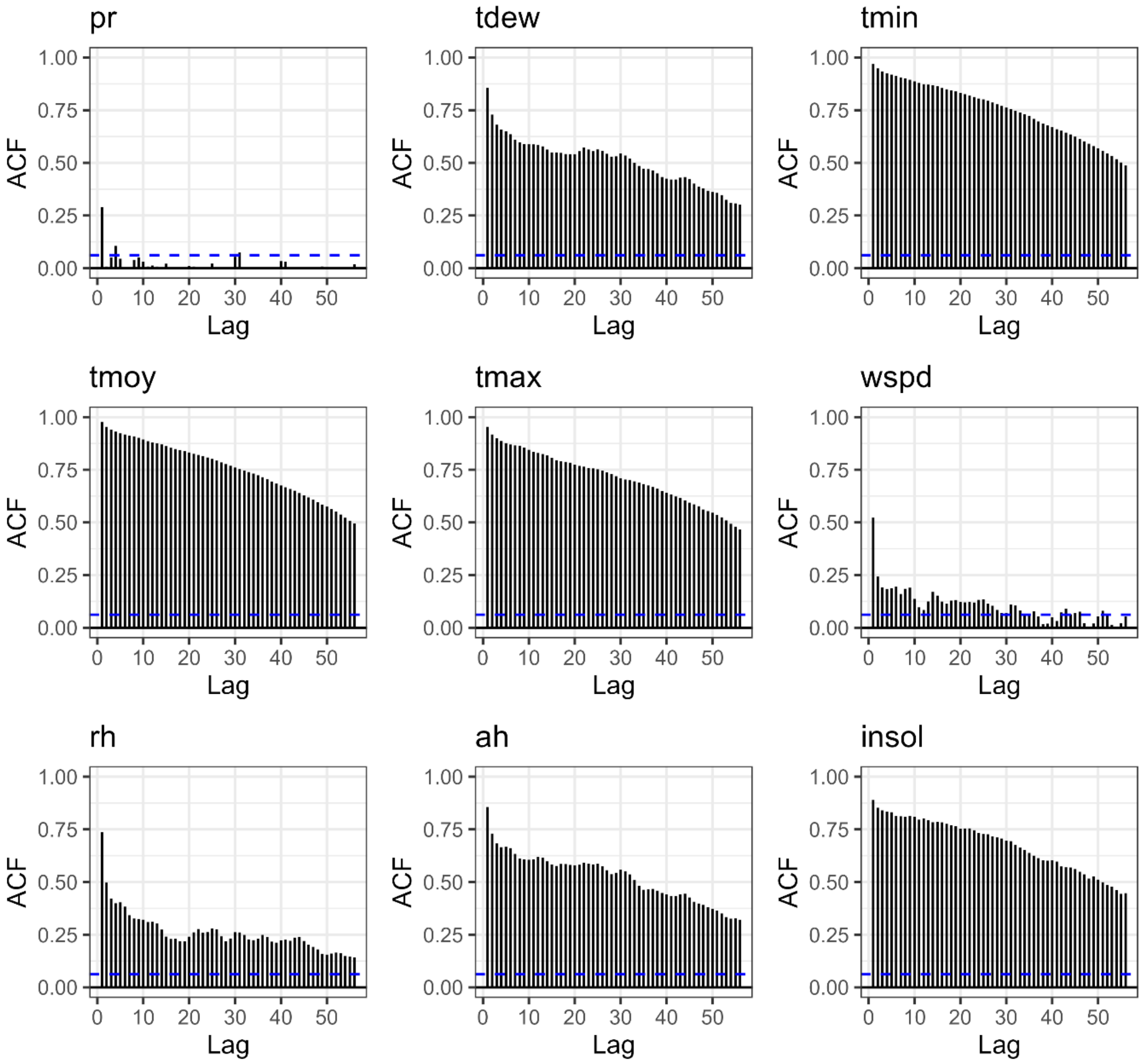

52) Uganda (UGA)

ACF plots for weather variables from January 1, 2020 to September 30, 2022

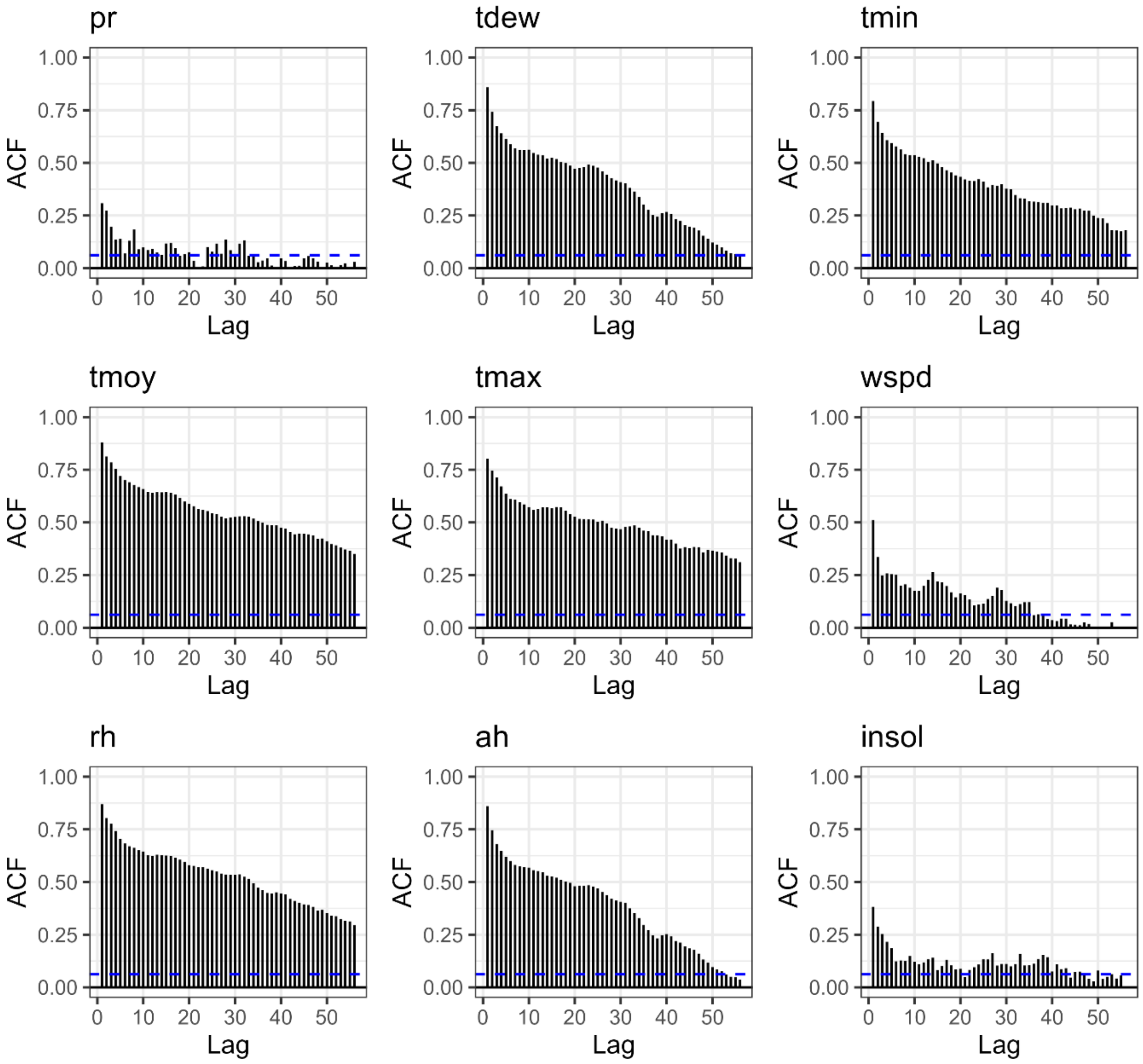

53) Zambia (ZMB)

ACF plots for weather variables from January 1, 2020 to September 30, 2022

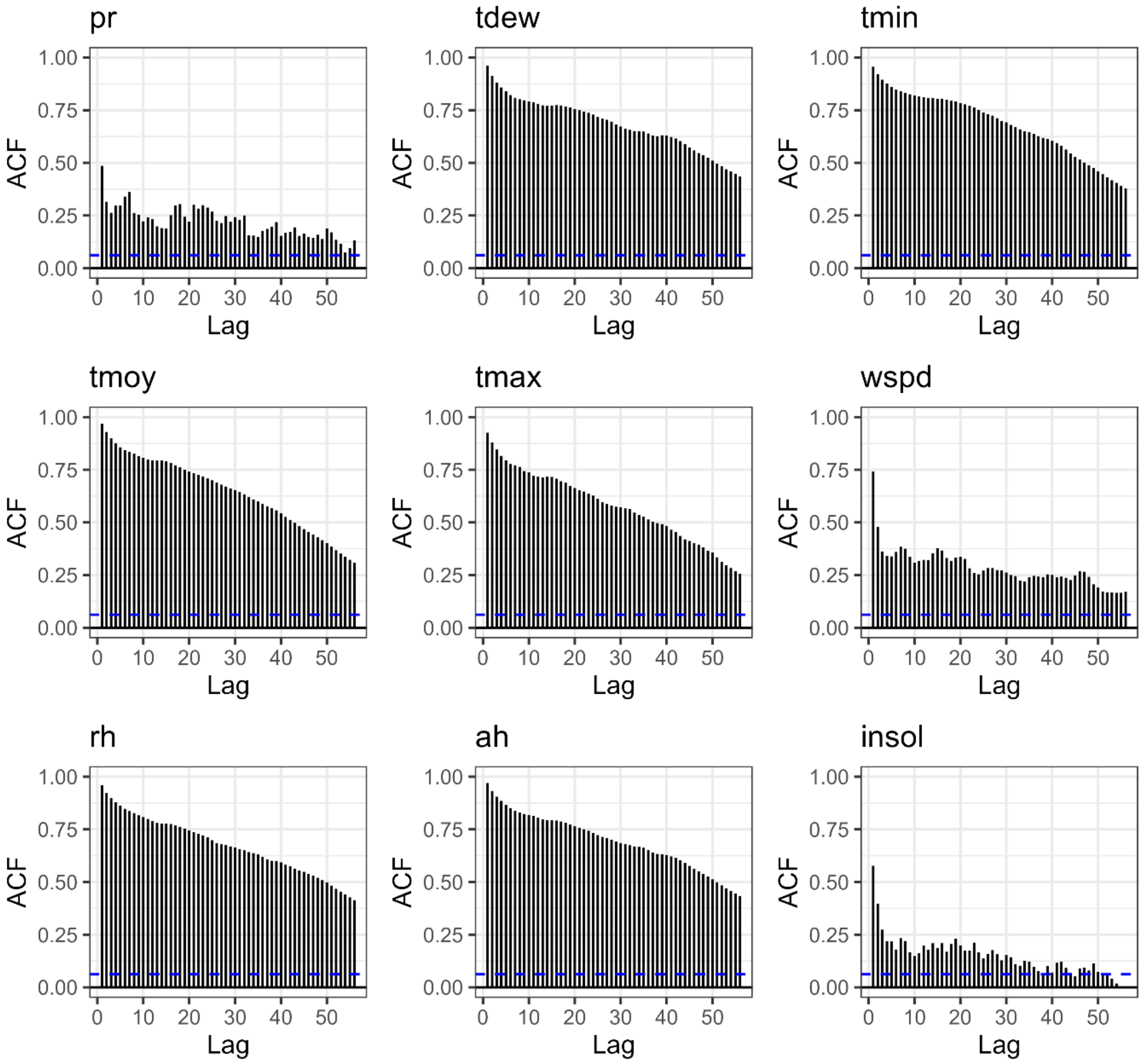

54) Zimbabwe (ZWE)

ACF plots for weather variables from January 1, 2020 to September 30, 2022

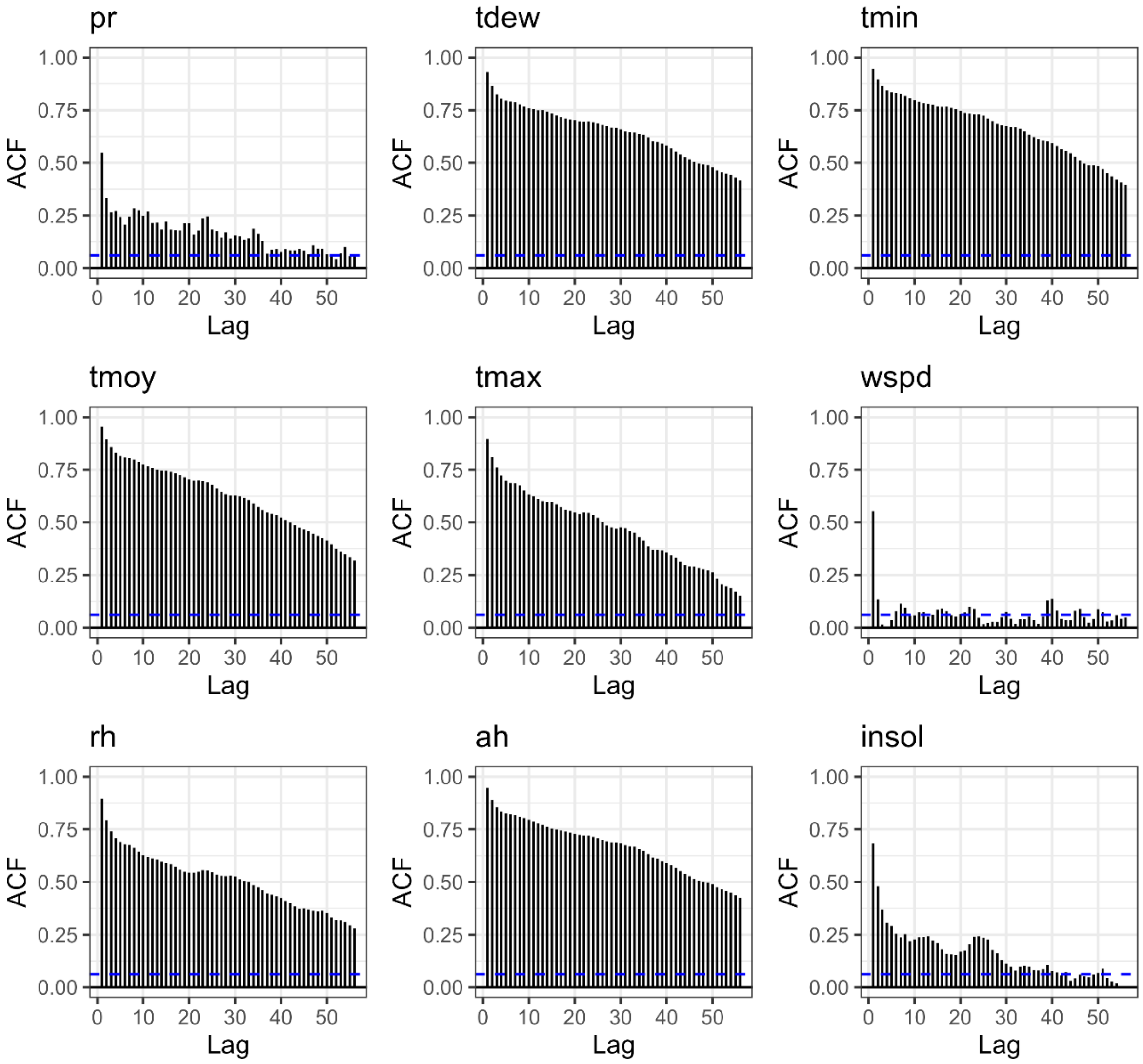

Supplement: Supplementary file 1 — Supplementary Information. [file 41598_2023_46007_MOESM1_ESM.pdf]
